# Statins suppress cell-to-cell propagation of α-synuclein by lowering cholesterol

**DOI:** 10.1038/s41419-023-05977-9

**Published:** 2023-07-27

**Authors:** Joo-Ok Min, Hoang-Anh Ho, Wonjae Lee, Byung Chul Jung, Sung Jun Park, Seokjoong Kim, Seung-Jae Lee

**Affiliations:** 1 Department of Biomedical Sciences, Neuroscience Research Institute, Convergence Research Center for Dementia, Seoul National University College of Medicine, Seoul, 03080 Republic of Korea; 2 Interdisciplinary Program in Neuroscience, College of Natural Sciences, Seoul National University, Seoul, Republic of Korea; 3 ToolGen, Inc, Seoul, Republic of Korea; 4 Neuramedy Co. Ltd, Seoul, Republic of Korea; 5 Present Address: Neuramedy Co. Ltd, Seoul, Republic of Korea; 6 Present Address: Nutritional Sciences and Toxicology Department, University of California Berkeley, Berkeley, CA USA

**Keywords:** Parkinson's disease, Parkinson's disease

## Abstract

Cell-to-cell propagation of protein aggregates has been implicated in the progression of neurodegenerative diseases. However, the underlying mechanism and modulators of this process are not fully understood. Here, we screened a small-molecule library in a search for agents that suppress the propagation of α-synuclein and mutant huntingtin (mHtt). These screens yielded several molecules, some of which were effective against both α-synuclein and mHtt. Among these molecules, we focused on simvastatin and pravastatin. Simvastatin administration in a transgenic model of synucleinopathy effectively ameliorated behavioral deficits and α-synuclein accumulation, whereas pravastatin had no effect. Because only simvastatin enters the brain effectively, these results suggest that inhibition of brain cholesterol synthesis is important in simvastatin effects. In cultured cells, accumulation of intracellular cholesterol, induced by genetic ablation of the *NPC1* gene or by pharmacological treatment with U18666A, increased α-synuclein aggregation and secretion. In contrast, lowering cholesterol using methyl-β-cyclodextrin or statins reversed α-synuclein aggregation and secretion in NPC1-knockout cells. Consistent with these observations, feeding a high-fat diet aggravated α-synuclein pathology and behavioral deficits in the preformed fibril-injected mouse model, an effect that was also reversed by simvastatin administration. These results suggest that statins suppress propagation of protein aggregates by lowering cholesterol in the brain.

## Introduction

The neurodegenerative diseases, Alzheimer’s disease (AD), Parkinson’s disease (PD) and Huntington’s disease (HD), are characterized by aggregation of specific proteins, namely tau, α-synuclein, and mutant huntingtin with expanded polyglutamine (mHtt) [1], respectively. These aggregates spread from a few discrete regions to wider brain regions through cell-to-cell propagation, and this spread of aggregates is considered to drive disease progression [2–5].

Notably, a growing body of evidence indicates that the propagation via exocytosis/endocytosis or by tunneling nanotubes is the underlying principle of the cell-to-cell propagation of α-synuclein, an abundant nerve terminal protein [6, 7]. For modeling the spreading of aggregate, α-synuclein preformed fibrils (PFFs), which were composed of short fragments of fibrils, were used to convert the endogenous α-synuclein into insoluble and hyperphosphorylated aggregates in both in vitro and in vivo [3, 8–12]. However, the mechanism of cell-to-cell propagation remains elusive, and agents that modulate this process have not been identified.

To solve these problems, in our previous study, we generated a dual-cell bimolecular fluorescence complementation (BiFC) assay system for quantitative assessment of α-synuclein propagation in living cells [13]. In the present study, we also developed a system for assaying mHtt propagation and adapted these two propagation assay systems for high-contents screening of drug libraries. From these drug library screens, we identified statins (pravastatin, simvastatin) as modulators in common of α-synuclein and mHtt propagation.

Statins, inhibitors of 3-hydroxy-3-methylglutaryl coenzyme A reductase (HMG-CoA reductase), reduce cholesterol levels through the inhibition of the biosynthesis of cholesterol [14]. Previous studies have shown that statins reduced α-synuclein accumulation by lowering cholesterol levels [15, 16]. Consistent with these findings, our further studies employing in vivo and in vitro models demonstrated the critical roles of brain cholesterol metabolism in the propagation of synucleinopathies. Our study provides that simvastatin can be an inhibitor of α-synuclein propagation and have a potential role as an agent for the treatment of synucleinopathies by lowering cholesterol levels.

## Materials and methods

### Animals

Adult (aged 6 months) male and female PrP-A53T mice (G2-3 line, C57BL6/J strain) and their wild-type (WT) littermates were used. C57BL/6 mice aged 10 weeks were used for the propagation model. All animals were housed under a 12/12-h light/dark cycle and had free access to food and water. All experimental procedures described below were performed in accordance with the Institutional guidelines for experimental animal care (SNU-190721-1-11) and use of the Seoul National University (Republic of Korea).

### Establishment of V1S and SV2 stable cell lines

The construction of stable V1S and SV2 cell lines has been previously described [13]. In brief, the V1S and SV2 cell lines were generated by electroporating SH-SY5Y cells with the Venus-α-syn (V1S) or α-syn (SV2) plasmid. The transfected cells were then isolated and selected using 600 μg/ml Geneticin (G418; Invitrogen 11811-031) until colonies were obtained. The stable V1S and SV2 cell lines were subsequently maintained in growth medium containing 200 μg/ml Geneticin.

### Generation of V1Q and QV2 stable cell lines

Vectors expressing human huntingtin exon 1 carrying the expanded polyQ repeats (Q97) were created by amplifying mHttQ97 products from a donor plasmid [17] and cloning them into pBiFC-VN173 (Addgene Plasmid #22010) and pBiFC-VC155 (Addgene Plasmid #22011) vectors. After transfection, positive clones were selected based on ampicillin resistance and confirmed by DNA sequencing of isolated colonies. Stable V1Q and QV2 cell lines were constructed by electroporating human neuroblastoma SH-SY5Y cells with N-terminal Venus-mHttQ97 (V1Q) or mHttQ97-Venus C-terminal (QV2) plasmid. After transfection, we isolated individual transfected cells and selected stably transfected cells by incubating with 300 μg/ml Geneticin to obtain colonies. Stable V1Q and QV2 cells were maintained in medium containing 200 μg/ml Geneticin.

### Drug screening

V1S and SV2 cells were prepared as described previously [13]. Briefly, V1S and SV2 cells co-cultured for 6 passages or V1Q and QV2 cells co-cultured for 3 passages were seeded onto a flat-bottom 96-well black plate (Greiner 655090) at 8 × 10^4^ cells per well. After 24 h, cells were incubated with drugs from the Prestwick library of FDA-approved drugs (final concentration, 10 μM) at 37 °C for 48 h. Nuclei were stained by incubating cells with 10 μg/ml Hoechst 33342 (Invitrogen H1399), and dead cells were detected by incubating with Topro-3 (1:1000) at 37 °C for 10 min prior to imaging. Cells were washed once in DMEM and then fresh medium without phenol red was added. Venus fluorescence in live cells was measured using an automated high-content screening reader (IN Cell Analyzer 2200; GE Healthcare). The resulting images were analyzed for inhibition of α-synuclein propagation compared to control DMSO-treated V1S and SV2 cells and inhibition of mHtt compared to control DMSO-treated V1Q and QV2 cells (normalized cutoff > 40%; *p*-value < 0.08, two-tailed unpaired Student’s *t*-test) using the IN Cell Developer Toolbox software and GraphPad 7.04. Control co-cultures were seeded separately on each plate and treated with DMSO. ‘Hit’ compounds identified in initial screens were subjected to a second screening using the same methods. Concentration-response relationships (1 nM, 10 nM, 0.1 μM, and 1 μM) were determined for selected drugs that modulated cell-to-cell propagation of α-synuclein and mHtt. A total of 3000 cells, incubated at 37 °C in a humidified CO_2_ incubator, were analyzed in each of three independent experiments.

### Establishment of NPC1-knockout cells

WT and NPC1-knockout cell lines were cultured and maintained in 200 μg/ml Geneticin. To establish WT cells expressing the LAMP1-GFP gene, we transfected SH-SY5Y cells with the LAMP1-GFP plasmid (Addgene Plasmid #34831) by electroporation and selected positive clones by incubating with 500 μg/ml of Geneticin. Selected LAMP1-GFP–positive cells were sorted by fluorescence-activated cell sorting (FACS) using a narrow gate (GFP RFU > 10^4^). NPC1-deficient cell lines were generated by transfecting stable LAMP1-GFP cells with single-guide RNA (sgRNA) and Cas9 protein. The target sequence in exon 1 used for NPC1 knockout was 5′-ACTAAGTCATATCCATCCTTTGG-3′. The gRNA was designed using an online tool (http://www.rgenome.net/cas-designer). Individual clones were isolated and confirmed by DNA sequencing and immunofluorescence staining.

### Dextran degradation assay

Cells were grown on poly-L-lysine–coated coverslips and incubated with 100 μg/ml of Alexa 568-conjugated dextran (MW, 10,000; Invitrogen D22912) for 2 h. After washing with DMEM, cells were incubated with fresh media for 1 h, then fixed with 4% paraformaldehyde (PFA; Sigma–Aldrich P6148). Fluorescence intensity was measured using a laser-scanning confocal microscope (Zeiss; LSM700).

### Amplex red cholesterol assay

Total cholesterol levels in cells were assessed in whole-cell lysates diluted 10 times in 1× Reaction buffer. The assay was performed by adding 50 μl of diluted samples or controls together with 50 μl of a working solution of 300 μM Amplex Red reagent containing 2 U/ml HRP, 2 U/ml cholesterol oxidase, and 0.2 U/ml cholesterol esterase into clear-bottomed black 96-well plates (Corning 3603). Reactions were performed by incubating plates for 30 min at 37 °C, protected from light, after which fluorescence was measured at excitation and emission wavelengths of 535 and 590 nm, respectively, using a Synergy Neo microplate reader (BioTek).

### Adenoviral overexpression and detergent extraction of cells

α-Synuclein was overexpressed in differentiated SH-SY5Y cells by infecting with adenoviral constructs, as described in a previous study [18]. The detailed procedures are described in Supplementary materials and methods.

### Western blotting

The efficiency of α-synuclein inhibition by statins in vitro was tested by Western blot analysis of whole-cell lysates. Western blotting was performed as previously described [18]. The detailed procedures are described in Supplementary materials and methods.

### Preparation of the mouse α-synuclein PFF (mα-synPFF) synucleinopathy model

The detailed procedures are described in Supplementary materials and methods.

### Animal treatment

Transgenic mice expressing A53T mutant α-synuclein under control of the PrP promoter were used. Briefly, adult (6-month old) transgenic mice and littermate WT mice were administered statins (pravastatin [Tocris 2318], simvastatin [Tocris 1965]; 1 mg/kg/d) daily for 3 months by oral gavage. Mice injected with PBS or mα-synPFF were fed either a normal chow diet (10% kcal from fat; Research Diets D12450H) or HFD (45% kcal from fat; Research Diets D12451). mα-synPFF–injected mice fed a HFD were orally administered statins or vehicle daily starting 1 week after surgery.

### Immunofluorescence staining

Cells seeded on poly-L-lysine–coated coverslips were incubated with 100 nM MitoTracker (Invitrogen M7512) for 30 min at 37 °C and fixed in 4% PFA in PBS for 30 min at room temperature. After fixation, cells were permeabilized in 0.1% Triton X-100 in PBS and incubated with blocking solution (5% bovine serum albumin, 3% goat serum in PBS). Then cells were incubated with the indicated primary antibodies: mouse anti-early endosome antigen 1 (EEA1) (BD Biosciences E41120, 1:1,000), rabbit anti-calnexin (Enzo life sciences ADI-SPA-860, 1:100), rabbit anti SREBP2 (Abcam ab30682, 1:100). After washing, cells were incubated with the corresponding fluorescent dye-conjugated secondary antibodies: Rhodamine Red-X (RRX)-conjugated goat anti mouse (Jackson ImmunoResearch 115-295-146, 1:500), RRX-conjugated goat antirabbit (Jackson ImmunoResearch 111-295-144, 1:500). Nuclei were stained with TOPRO-3 iodide (Invitrogen T3605, 1:1,000). Cells were co-stained for cholesterol by incubating with 50 μg/ml of Filipin (Sigma–Aldrich SAE0088) in PBS for 2 h at room temperature. Cells were mounted onto slide glasses with Prolong Gold Antifade Reagent (Invitrogen P36930).

Sections (40-μm thick) were cut with a vibratome, washed three times in PBS, and incubated for 1 h at room temperature with blocking solution (0.1% Triton X-100, 10% goat serum in PBS). Sections were incubated overnight at 4 °C on a shaker with rabbit anti-MAP2 primary antibody (Abcam AB5622, 1:500), diluted in PBST (0.1% Triton X-100 in PBS), followed by incubation of free-floating sections with Alexa 647-conjugated goat anti rabbit secondary antibody (Jackson ImmunoResearch 111-605-144, 1:200) for 90 min. After washing three times in PBST, sections were stained for cholesterol by incubating with 5 μM BODIPY-cholesterol (480/508; Cayman Chemical #24618) for 1 h. Sections were washed twice in PBS and then incubated in PBS containing DAPI (4′,6-diamidino-2-phenylindole dihydrochloride; Molecular Probe D1306), diluted 1:1000. Sections were rinsed three times in PBS and slide-mounted. Fluorescence images were obtained with a confocal microscope (Zeiss LSM700) and BODIPY-CHOL^+^ structures were quantified using ImageJ and ZEN 2012 software.

### Forelimb grip strength test

Mice were hung by their front paws on a metal grid, and the maximal force on their forelimbs was measured two times. Values were averaged and mean values were normalized against mouse body weights.

### Balance beam test

The beam apparatus consists of a 1-meter beam (2 cm diameter) with a height of 50 cm. Mice were habituated to the black box at the end point of the apparatus for 2 min, after which each mouse was trained to walk on the beam. Mice were then placed at one end of the beam, and the time it took the mouse to reach the black box at the other end was recorded. The total distance moved in crossing the beam and the number of falls and slips were recorded and analyzed.

### Immunohistochemistry

Immunohistochemistry was performed as previously described [19]. The detailed procedures for immunohistochemistry are described in Supplementary materials and methods.

### Statistical analysis

All statistical analyses were performed using GraphPad Prism version 7.04 and ImageJ software. All data are presented as means ± standard error of the mean (SEM). The significance of differences between two means was assessed using a two-tailed unpaired Student’s *t*-test; one-way analysis of variance (ANOVA) with Dunnett’s post-hoc test, one-way ANOVA with Tukey’s post-hoc test, or two-way ANOVA with Dunnett’s post-hoc test was used for analysis of differences among multiple means. *P*-values < 0.05 were considered statistically significant; individual *p*-values are shown in figure legends.

## Results

### Establishment of a high-content screening (HCS) system for identification of propagation modulators

In our previous study, we established a dual-cell BiFC system for measuring cell-to-cell propagation of α-synuclein. This system is composed of two stable cell lines, V1S and SV2, that express α-synuclein protein together with the N-terminus and C-terminus, respectively, of Venus fluorescence protein [13]. To identify propagation modulators common to different neurodegenerative diseases, we additionally generated stable cell lines expressing mHtt and V1Q or QV2 as a dual-cell BiFC system (Supplementary Fig. 1A). Western blot analyses confirmed that both mHtt cell lines expressed the appropriate fusion proteins (Supplementary Fig. 1A). Co-culture of these cells resulted in the appearance of intracellular fluorescent puncta under live-cell conditions (Supplementary Fig. 1B), indicating cell-to-cell propagation of mHtt proteins. We then validated the HCS protocols for propagation of both α-synuclein and mHtt and confirmed that only co-cultures generated BiFC signals (Supplementary Fig. 1C, D). Using this HSC system, we also confirmed that bafilomycin A1, a lysosomal acidification blocker known to increase cell-to-cell propagation of protein aggregates [20, 21], increased the propagation of both α-synuclein and mHtt (Supplementary Fig. 1C, D).

### Statins are inhibitors in common of α-synuclein and mHtt cell-to-cell propagations

**Fig. 1:**
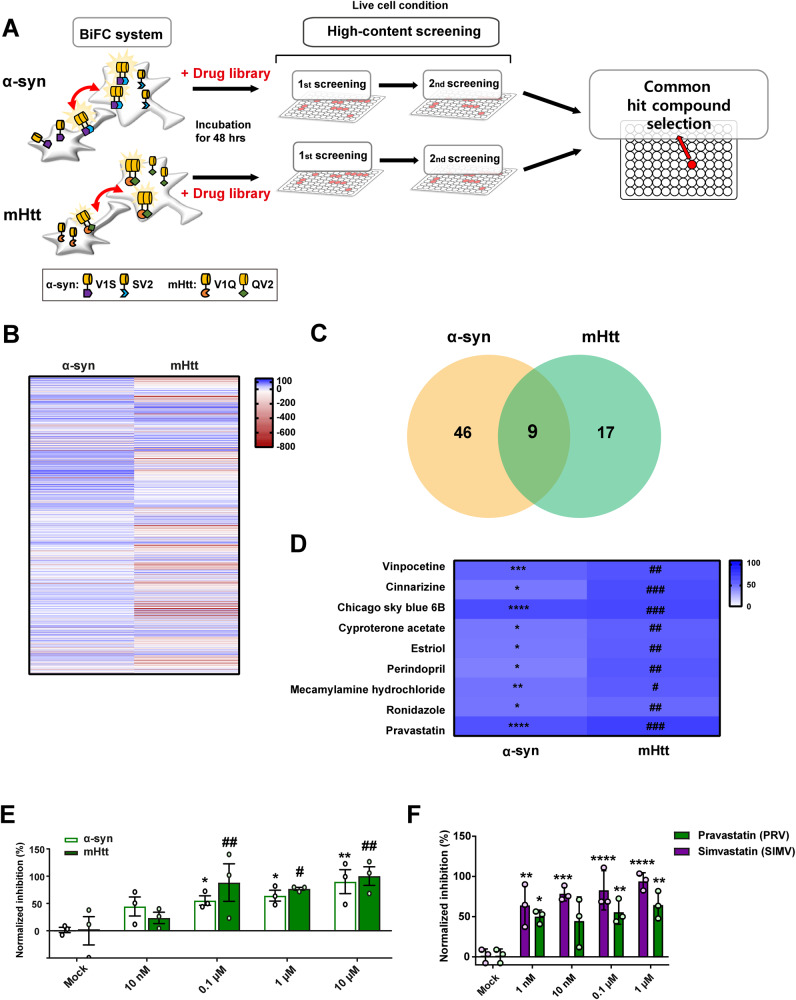
High-content drug screening for chemical modifiers of α-synuclein and mHtt propagation. **A** Schematic workflow of drug screening in BiFC cell models. **B** Heat map representing the mean percentage changes in aggregate propagation. Details are presented in Supplementary Table 1. **C, D** Comparison of secondary screening results for α-synuclein and mHtt. Compounds with shared inhibitory effects on α-synuclein and mHtt propagation are presented as a Venn diagram (**C**) and heat map (**D**). Details are presented in Supplementary Tables 2 and 3. **E** Concentration-response relationship for pravastatin (PRV). **F** Summary data showing that both PRV and simvastatin (SIMV) significantly reduced α-synuclein propagation in a concentration-dependent manner. Data are presented as means ± SEM (*p < 0.05, ^#^*p* < 0.05, **p < 0.005, ^##^*p* < 0.005, ***p < 0.0005, ^###^*p* < 0.0005, ****p < 0.0001, ^####^*p* < 0.0001; one-way ANOVA with Dunnett’s post-hoc test).

Using the HSC systems described above, we screened an FDA-approved drug library. After primary screening for modifiers of α-synuclein propagation, we subjected 142 primary ‘hits’ to a secondary screening (Fig. 1A, B; Supplementary Table 1). Ultimately, we selected 55 drugs that significantly inhibited the propagation of α-synuclein aggregates (Fig. 1C, Supplementary Table 2). The initial screen for modifiers of mHtt propagation resulted in 44 hit compounds, a number that was reduced to 26 after the secondary screening (Fig. 1C; Supplementary Table 3). Of these 26 modifier compounds, 9 were modifiers in common that significantly decreased the propagation of both α-synuclein and mHtt (Fig. 1D).

Among these common modifiers, we chose pravastatin (normalized inhibition percentage > 60% for both α-synuclein and mHtt), a competitive inhibitor of 3-hydroxy-3-methylglutaryl coenzyme A reductase (HMG-CoA reductase), for further characterization (Fig. 1D). We confirmed that pravastatin significantly inhibited propagation of both α-synuclein and mHtt in a concentration-dependent manner (Fig. 1E). To confirm that these effects are generalizable to other statins, we also examined the effects of another commonly used statin, simvastatin. Like pravastatin, simvastatin also exerted concentration-dependent inhibitory effects on α-synuclein propagation (Fig. 1F). These results indicate that the cholesterol-lowering agents, pravastatin and simvastatin, effectively inhibit cell-to-cell propagation of α-synuclein in an in vitro cell model.

### Simvastatin, but not pravastatin, ameliorates motor impairments and neuropathological features of synucleinopathy

**Fig. 2:**
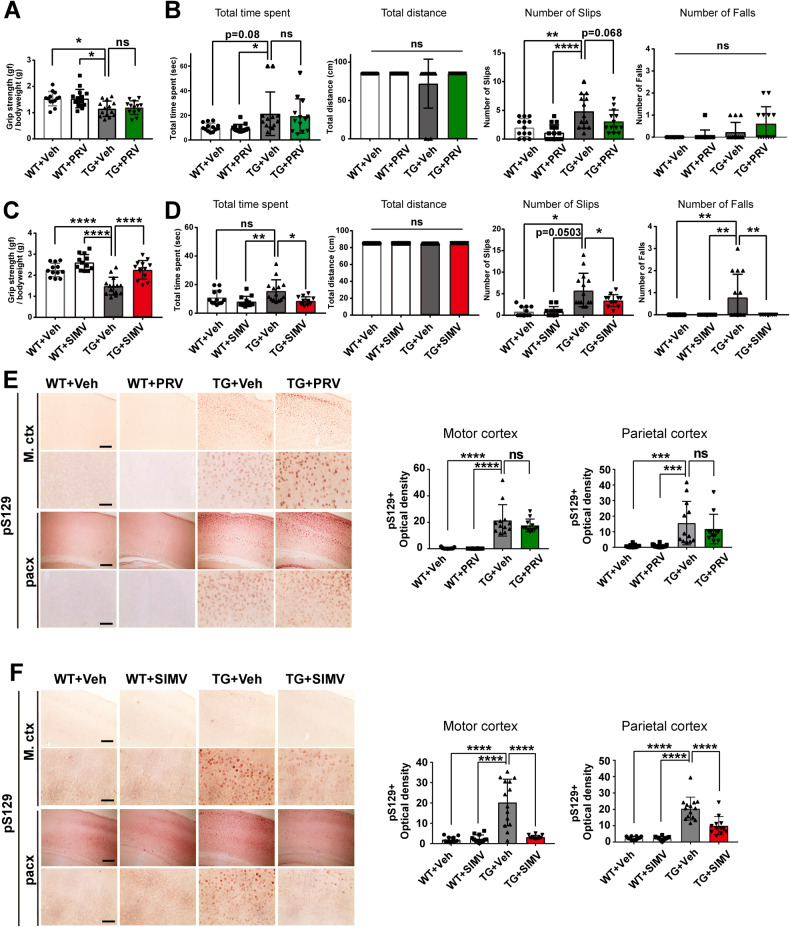
Simvastatin significantly alleviates motor deficits and synucleinopathy lesions, whereas pravastatin does not. **A, C** Forelimb grip strength test. **B, D** Balance beam test. **A, B** Pravastatin administration. **C, D** Simvastatin administration. **E, F** Immunohistochemical analysis of pS129 in the motor cortex (M.ctx) and parietal cortex (pacx) of A53T mice treated with pravastatin (**E**) or simvastatin (**F**). Scale bar: 50 μm. **A**–**F** *n* = 11–15 mice per group. All data are presented as means ± SEM (*p < 0.05, **p < 0.005, ***p < 0.0005, ****p < 0.0001; one-way ANOVA with Dunnett’s post-hoc test).

Because it is hydrophilic, pravastatin crosses the blood–brain barrier (BBB) poorly, whereas simvastatin, which is lipophilic, has a greater capacity to penetrate the BBB [22, 23]. To determine the effects of statins in vivo, we administrated these agents daily (1 mg/kg/d) for 3 months by oral gavage in a transgenic mouse model overexpressing the A53T mutant form of α-synuclein [24]. Mice were assessed for muscle strength with a forelimb grip strength test (Fig. 2A, C) and for motor coordination with a balance beam test (Fig. 2B, D). Simvastatin administration significantly alleviated behavioral deficits in both motor tests, whereas pravastatin was not effective in either test (Fig. 2C, D). Next, we examined phosphorylated-α-synuclein (pS129)–positive inclusions by immunohistochemistry. Consistent with behavioral test results, simvastatin-treated transgenic mice showed significant reductions in pS129- immunoreactivity in the motor cortex (85%) and parietal cortex (49%) compared with vehicle-treated mice (Fig. 2E, F). In contrast, pravastatin administration showed no effect on pS129-α-synuclein immunoreactivity (Fig. 2E). To determine cholesterol accumulation in the brains, we measured the BODIPY-Cholesterol^+^ (BODIPY-CHOL^+^) structures in the motor cortex (M.ctx) and parietal cortex (pacx). While there were no significant differences in the number of BODIPY-CHOL^+^ structures between the TG mice treated pravastatin (PRV) group and the TG mice treated with vehicle (Supplementary Fig. 2A), the number of BODIPY-CHOL^+^ structures in TG mice administered with simvastatin (SIMV) was significantly lower than TG treated with vehicle (Supplementary Fig. 2B). Therefore, only simvastatin, not pravastatin, affected the cholesterol in the brain. These results suggest that simvastatin effectively rescues both behavioral and neuropathological deficits in a synucleinopathy mouse model. Moreover, the fact that only simvastatin, and not pravastatin, showed these effects suggests that regulation of cholesterol within the brain is important in modifying these phenotypes in mice.

### Modifying cholesterol metabolism regulates α-synuclein secretion

**Fig. 3:**
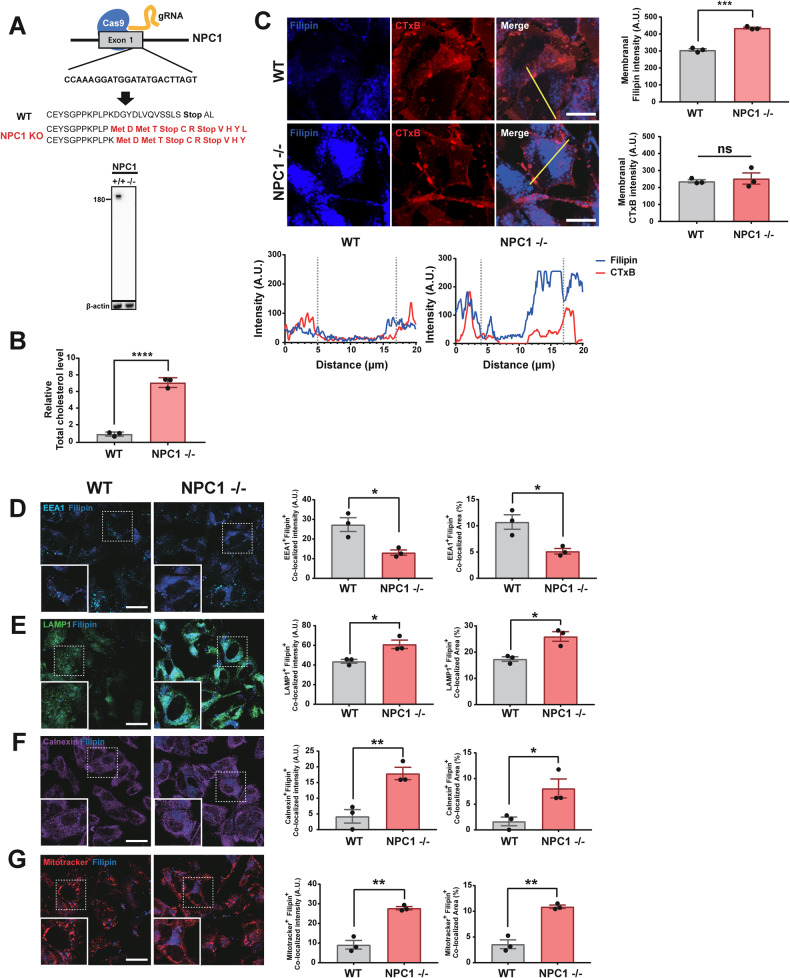
Characterization and analysis of cholesterol levels in NPC1-KO cells. **A** Construction of the NPC1-KO cell line using the CRISPR-Cas9 system. Scheme for NPC1 knockout (top) and representative Western blot (bottom). **B** Intracellular total cholesterol levels in NPC1-KO cells and WT (LAMP1-GFP) cells. **C** Analysis of the plasma membranal cholesterol levels in WT and NPC1-KO cells. Filipin marks cholesterol, and cholera toxin B (CTxB) binds GM1 and marks the lipid rafts mostly in the plasma membrane. Yellow lines in the confocal images indicate the distance analyzed for the graphs below. Graphs on the right show the levels of filipin and CTxB binding in the plasma membrane. **D**–**G** Analysis of cholesterol distribution in intracellular organelles, such as early endosome (**D**), late endosome/lysosome (**E**), endoplasmic reticulum (**F**), and mitochondria (**G**). Colocalization of filipin staining with EEA1 (early endosome antigen 1) (**D**), LAMP1 (Lysosomal-associated membrane protein 1) (**E**), calnexin (**F**), and mitotracker (**G**) were analyzed. Colocalization was expressed as either colocalized fluorescence intensity (left graphs) or colocalized area (right graphs). All data are presented as means ± SEM (*p < 0.05, **p < 0.005, ***p < 0.0005, ****p < 0.0001; two-tailed unpaired Student’s *t*-test).

Because statins are inhibitors of cholesterol biosynthesis, we asked whether the observed inhibition of α-synuclein propagation by statins reflects modification of cholesterol metabolism by these agents or off-target effects. To resolve this, we modified cholesterol metabolism in a neuronal cell model using both genetic and pharmacological approaches. To examine the effects of intracellular cholesterol accumulation on α-synuclein secretion, we established homozygous NPC1-knockout (KO) cells using the CRISPR-Cas9 system. To make this cell line, SH-SY5Y cells stably expressing LAMP1-GFP were transfected with sgRNA and Cas9 protein, which introduced stop codons in target sequences on both alleles to generate a homozygous knockout mutant (Fig. 3A). Mutations in the *NPC1* gene are responsible for Niemann-Pick disease type C1, a lysosomal storage disease that results in intracellular cholesterol accumulation and synucleinopathy lesions [25, 26]. Cholesterol levels were significantly increased in NPC1-KO cells compared with WT cells (Fig. 3B). Increasing cholesterol levels in the plasma membrane causes an increase in membrane rigidity [27], which could affect the rate of endocytosis and exocytosis. To determine the levels of the plasma membranal cholesterol in WT and NPC1-knockout cells, we performed co-staining with filipin and Cholera Toxin B subunit (CTxB). CTxB binds strongly to GM1, an established constituent of the lipid rafts [28], and also marks the plasma membrane in the cross-sectional analysis. Image analysis showed a significant increase in cholesterol accumulation in the plasma membrane in NPC1-knockout cells, while the levels of CTxB-positive lipid rafts were not significantly changed (Fig. 3C).

In addition, we have determined the intracellular locations where cholesterol is accumulated in NPC1-knockout cells. Intracellular cholesterol was stained with Filipin, and the extent of cholesterol accumulation in each organelle was analyzed with colocalization of filipin staining with organelle markers, such as EEA1 (early endosomes), LAMP1 (late endosomes/lysosomes), calnexin (endoplasmic reticulum), and mitotracker (mitochondria). The degree of colocalization was expressed as either colocalized fluorescence intensity or colocalized area. NPC1 is localized in the late endosomes/lysosomes as a transmembrane protein that plays a key role in modulating cellular cholesterol trafficking [29]. As expected, cholesterol was significantly increased in late endosomes/lysosomes in NPC1-knockout cells (Fig. 3E). Cholesterol was also increased in the ER of NPC1-knockout cells (Fig. 3F). The oxysterol-binding protein homolog, ORP1L, located in the late endosome/lysosome can interact with ER-localized vesicle associated membrane protein (VAMP)-associated proteins (VAPs) under sterol depletion conditions [30]. These proteins can transport cholesterol in the opposing direction from ER to endosome. Therefore, cholesterol accumulation in lysosomes due to NPC1 deficiency may affect cholesterol trafficking and accumulation in the ER. We next investigated mitochondrial cholesterol in WT and NPC1-knockout cells. Recent studies have shown that cholesterol accumulation in mitochondria occurs through the regulation of STARD1 expression. STARD1 plays a crucial role in trafficking of cholesterol to mitochondrial inner membrane and is regulated by lysosomal acid ceramidase (ACDase). In NPC1 -/- mice, ACDase is downregulated, leading to the upregulation of STARD1, and subsequent mitochondrial cholesterol accumulation [31–34]. Furthermore, a recent study found that the lysosome-mitochondria contact sites were expanded through the late endosomal sterol-binding protein STARD3 in the absence of NPC1 [35]. Consistent with these previous studies, we found a significant increase in mitochondrial cholesterol accumulation in NPC1-knockout cells compared to WT cells (Fig. 3G). In contrast to these findings, we observed a significant decline in cholesterol localization in the early endosomes in NPC1-knockout cells (Fig. 3D). The reason for this decrease is not clear.

**Fig. 4:**
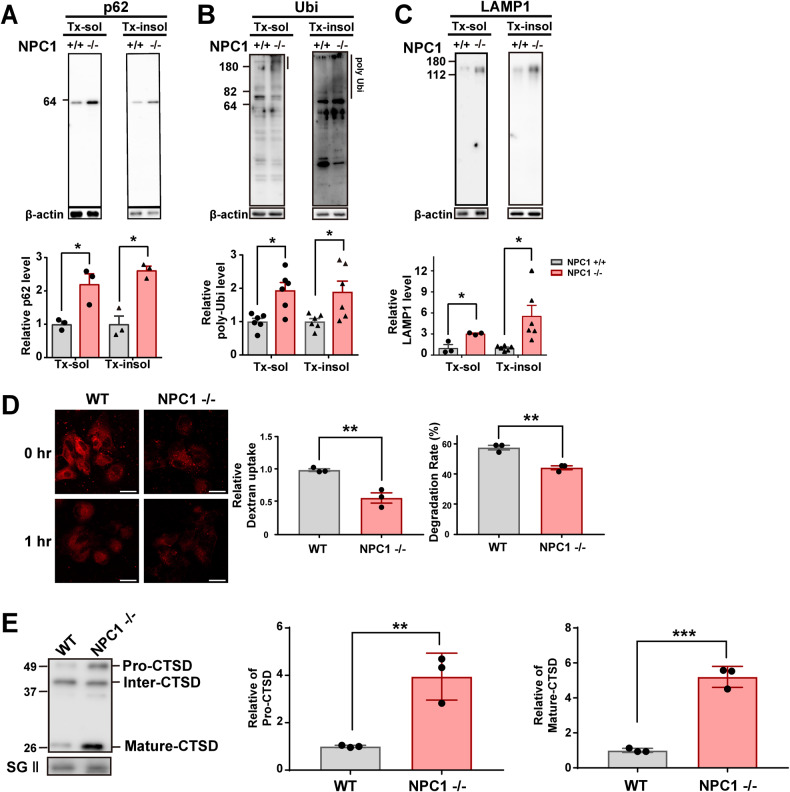
Characterization of NPC1-KO cells. **A**–**C** Western blot analysis of p62 (**A**), ubiquitin (Ubi) (**B**) and LAMP1 (**C**). **D** Dextran uptake and degradation rate in WT and NPC1-KO cells incubated with fluorescence-labeled dextran for 2 h and chased for 1 h. **E** Levels of secreted pro-CTSD and mature-CSTD in the media. All cell experiments were repeated triplicate determination of each sample. All data are presented as means ± SEM (*p < 0.05, **p < 0.005, ***p < 0.0005, ****p < 0.0001; two-tailed unpaired Student’s *t-*test).

We then verified that an NPC1 deficiency induces lysosomal defects, as demonstrated by accumulation of the lysosomal substrates p62 and poly-ubiquitinated proteins, increased expression of the lysosomal marker protein LAMP1, and a reduction in the dextran degradation rate (Fig. 4A–D). We also found that the levels of both pro-cathepsin D (CTSD) and mature-CTSD significantly increased in the media of NPC1-knockout cells compared with that of WT cells (Fig. 4E). Collectively, these data indicate that NPC1 knockout results in increased intracellular cholesterol, lysosomal defects, and increased lysosomal exocytosis.

**Fig. 5:**
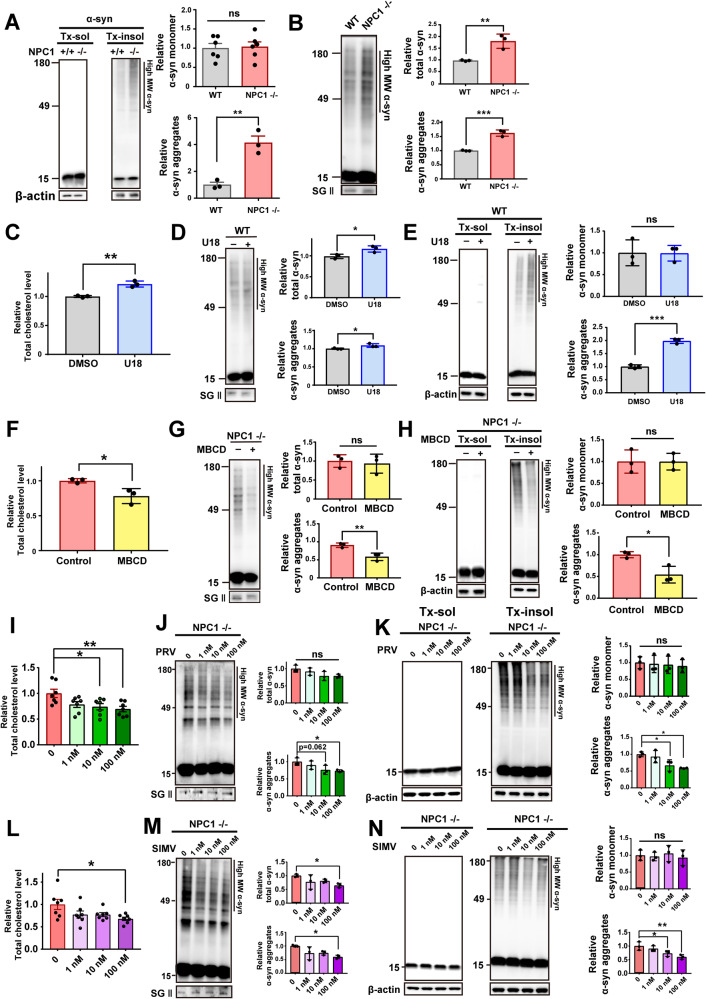
Cholesterol levels regulate α-synuclein aggregation and secretion. **A, B** Accumulation of α-synuclein aggregates (**A**) and secretion α-synuclein (**B**) in NPC1-KO and WT cells. **C** Total intracellular cholesterol levels in WT cells treated with or without U18. **D, E** Secretion (**D**) and intracellular accumulation (**E**) of α-synuclein aggregates in WT cells treated with and without U18. **F**–**H** Total cholesterol levels (**F**), intracellular accumulation of α-synuclein aggregates (**H**), and secretion of α-synuclein aggregates (**G**) in NPC1-KO cells treated with or without MBCD. **I**–**K** Intracellular total cholesterol (**I**), α-synuclein secretion (**J**), and intracellular α-synuclein aggregates (**K**) in NPC1-KO cells treated with or without pravastatin. **L**–**N** Intracellular total cholesterol (**L**), α-synuclein secretion (**M**), and intracellular α-synuclein aggregates (**N**) in NPC1-KO cells treated with or without simvastatin. All cell experiments were repeated triplicate determination of each sample. All data are presented as means ± SEM (*p < 0.05, **p < 0.005, ***p < 0.0005, ****p < 0.0001; two-tailed unpaired Student’s *t-*test [**A**–**H**], one-way ANOVA with Dunnett’s post-hoc test [**I**–**N]**).

The previous studies showed that α-synuclein aggregates could be degraded by lysosome and secreted via lysosomal exocytosis [19, 36]. Therefore, we examined α-synuclein aggregation and secretion in NPC1-KO cells. Upon expression of α-synuclein in NPC1-KO cells, both the levels of intracellular Triton X-100–insoluble α-synuclein and secreted α-synuclein were increased (Fig. 5A, B). To verify the effects of NPC1 knockout, we expressed human NPC1 back to the knockout cells using an adenoviral vector. When the expression of NPC1 was restored in NPC1-knockout cells (Supplementary Fig. 3A), the levels of α-synuclein aggregation and secretion were reduced to the levels of WT cells, confirming that the α-synuclein phenotypes observed in the NPC1-knockout cells were indeed due to the NPC1 deficiency (Supplementary Fig. 3B–D). We then examined the effects of U18666A (U18), which is known to increase cholesterol levels in cells by causing cholesterol trafficking defects [37]. Treatment of naive SH-SY5Y cells with U18 increased the levels of intracellular total cholesterol (Fig. 5C) and also increased α-synuclein aggregates in both media and cell lysates compared with vehicle-treated cells (Fig. 5D, E). We then asked whether the effects of NPC1-KO are reversed by lowering cellular cholesterol levels using a pharmacological strategy employing methyl-β-cyclodextrin (MBCD) and statins. MBCD extracts cholesterol from the plasma membrane, whereas statins inhibit biosynthesis of cholesterol. Although the initial sits of actions are different for these agents, intracellular cholesterol flow eventually influences the levels of cholesterol in many intracellular organelles. We confirmed that treatment of NPC1-KO cells with MBCD or statin (simvastatin or pravastatin) lowered the levels of total cholesterol as well as levels of intracellular and secreted α-synuclein aggregates (Fig. 5F–N). These data suggest that intracellular cholesterol accumulation caused by either NPC1-KO or U18 treatment increases α-synuclein aggregation and secretion, and that the effects of NPC1 knockout are reversed by the cholesterol-lowering agents, MBCD and statins.

### Downregulation of SREBP2 expression decreased α-synuclein aggregation and secretion

**Fig. 6:**
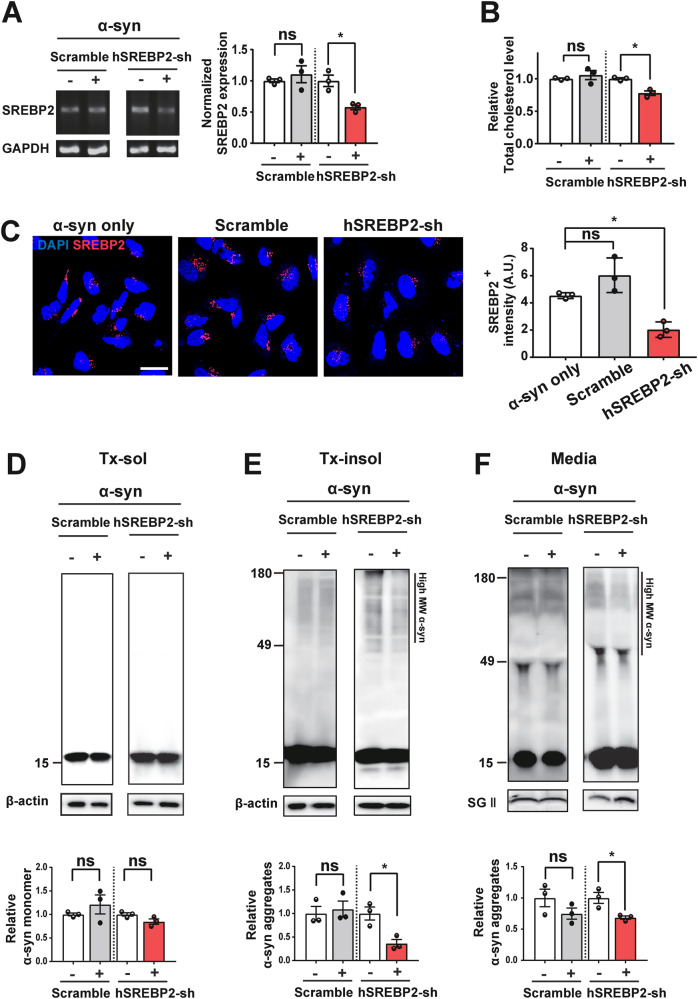
Knockdown of SREBP2 expression decreases α-synuclein aggregation and secretion. **A** RT-PCR gel images and the relative expression of SREBP2 normalized to the GAPDH. **B** The total cholesterol levels in differentiated SH-SY5Y cells. The cells were either infected with α-synuclein adenovirus alone (left image) or co-infected with α-synuclein and scramble RNA or SREBP2 shRNA adenovirus. **C** Immunofluorescence analysis of SREBP2 expression in differentiated SH-SY5Y cells. **D**–**F** Western blot analysis of the Triton X soluble (**D**), Triton X insoluble (**E**), and media (**F**) fractions. All data are presented as means ± SEM (*p < 0.05, **p < 0.005, ***p < 0.0005, ****p < 0.0001; two-tailed unpaired Student’s *t*-test [**A**, **B**, **D**–**F**], one-way ANOVA with Tukey’s post-hoc test [**C**]). Scale bars: 20 μm.

To confirm the role of cholesterol dyshomeostasis in α-synuclein aggregation and secretion, we downregulated the SREBP2 (Sterol regulatory element-binding protein 2) expression by using shRNA. SREBP2 regulates transcription of many genes encoding enzymes that are required for cholesterol biosynthesis, such as HMG-CoA reductase (HMGCR) [38, 39]. Expression of SREBP2 shRNA effectively reduced the mRNA and protein levels of SREBP2 as well as the levels of total cholesterol (Fig. 6A–C). Downregulation of SREBP2 expression, hence the reduction in the total cellular cholesterol synthesis, significantly decreased the Triton-insoluble α-synuclein aggregates and secretion of these aggregates (Fig. 6D–F). These results further validate our conclusion that intracellular cholesterol homeostasis is an important factor in regulation of α-synuclein aggregation and secretion.

### High-fat diet feeding worsens synucleinopathy phenotypes, an effect reversed by simvastatin treatment

**Fig. 7:**
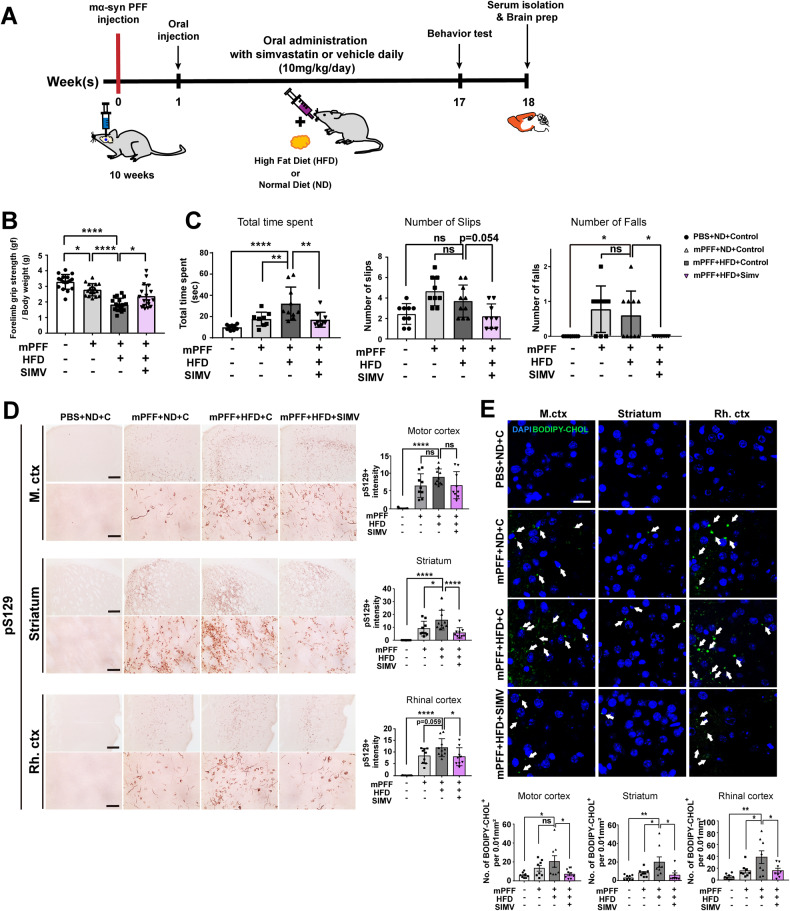
Effects of HFD feeding and simvastatin on motor functions and synucleinopathy lesions. **A** Experimental timeline of simvastatin administration and behavioral tests. **B** Forelimb grip strength test. **C** Balance beam test. **D** Immunohistochemical analyses of pS129 in the striatum, motor cortex (M.ctx), and rhinal cortex (Rh.ctx). **E** HFD-induced cholesterol accumulation in different brain regions, with and without simvastatin treatment. Scale bar: 20 μm. All data are presented as means ± SEM (*p < 0.05, **p < 0.005, ***p < 0.0005, ****p < 0.0001; two-way ANOVA with Tukey’s post-hoc test [**B**, **C**], one-way ANOVA with Dunnett’s post-hoc test [**D**, **E**]). All mice and brain tissues from mice were run individually with groups of 9 to 10 mice.

To further clarify the role of cholesterol in the progression of synucleinopathy, we examined the effects of a high-fat diet (HFD) in the mouse PFF (preformed fibril) model in which injection of preformed mouse α-synuclein fibrils (mα-synPFF) into the striatum leads to a synucleinopathy phenotype. One week after PFF injection, mice were placed on a normal diet or HFD regimen with daily administration of either simvastatin or vehicle for 4 months (Fig. 7A). Weekly monitoring of mouse body weight confirmed a significant increase in weight in mice fed a HFD (Supplementary Fig. 4A). HFD feeding also increased serum levels of total cholesterol and the number of lipid droplets in adipocytes; both effects were reversed by simvastatin treatment (Supplementary Fig. 4B–D). We next examined motor behaviors in these mice using a forelimb grip strength test and balance beam test (Fig. 7B, C). Both muscle strength and motor coordination were impaired following PFF injection and were further aggravated in mice fed a HFD. These detrimental effects of a HFD were rescued by simvastatin administration.

mα-synPFF injection caused development of pS129-positive inclusions in various brain regions, including the motor cortex, rhinal cortex, and striatum (Fig. 7D). Feeding a HFD exacerbated pS129 lesions in these regions, whereas administration of simvastatin significantly ameliorated these synucleinopathy lesions. Finally, we measured the accumulation of BODIPY-cholesterol^+^ (BODIPY-CHOL^+^) structures in brain tissues under these conditions (Fig. 7E). We confirmed elevated accumulation of cholesterol in each of these brain regions in HFD-fed mice compared with mice fed normal chow. Notably, simvastatin administration significantly reduced intracerebral BODIPY-CHOL^+^ structures. Taken together, these results indicate that higher BODIPY-CHOL^+^ structures in the brain accelerate propagation of α-synuclein aggregates, and that simvastatin ameliorates behavioral deficits and lesions associated with synucleinopathy.

## Discussion

In the current study, we screened a drug library to discover small-molecule modifiers of cell-to-cell propagation of α-synuclein and mHtt protein aggregates. One of the hit compounds in common identified during screening was pravastatin, an inhibitor of cholesterol biosynthesis. Another cholesterol-lowering agent, simvastatin, also showed inhibitory effects on propagation of α-synuclein. To verify that cholesterol regulates α-synuclein aggregation and propagation, we manipulated cellular cholesterol levels using both genetic (NPC1 mutation) and pharmacological strategies. Both approaches demonstrated that elevation of cellular cholesterol levels led to an increase in α-synuclein aggregation and secretion, and that these increases were reversed by treatment with cholesterol-lowering drugs. Also, simvastatin administration alleviated motor behavioral deficits and synucleinopathy lesions, whereas feeding PFF model mice a HFD increased the levels of brain cholesterol and worsened synucleinopathy phenotypes in two independent mouse models. Only the BBB-penetrating simvastatin, and not the hydrophilic pravastatin, was effective in alleviating synucleinopathy phenotypes.

How does cholesterol regulate α-synuclein aggregation and propagation? One way might be through the regulation of the lipid rafts. Cholesterol is a key component of the lipid rafts of the plasma membrane and other internal membranes and contributes to the membrane fusion in the process of endocytosis/exocytosis [40, 41]. For example, during synaptic transmission, the activities of pre- and post-synaptic proteins can be regulated by cholesterol [40, 42–44]. The previous studies have shown that α-synuclein can be propagated via exosomes enriched in cholesterol [45, 46]. Given these roles of cholesterol and lipid rafts in endocytosis and exocytosis, it would be tempting to suggest the role of the lipid rafts in α-synuclein propagation. Although our current study did not pursue the role of the lipid rafts per se in α-synuclein propagation, the effects of changes in cholesterol levels, especially in the plasma membranes, on a-synuclein propagation suggest possible roles of lipid rafts in this process. Future investigations into the lipid rafts could unveil the relationship between cholesterol metabolism and α-synuclein propagation.

Epidemiological studies have established an inverse correlation between the risk of neurodegenerative diseases, such as AD and PD, and statin intake in humans [47–50]. However, how cholesterol/statins regulate neurodegenerative diseases is not fully understood. In the case of AD, different isoforms of apolipoprotein E (apoE), which is important in cholesterol homeostasis and transport, differentially affect Aβ deposition, with the E4 isoform being the most effective in facilitating Aβ deposition and plaque formation [51]. Acute cholesterol exposure in neurons accelerates amyloid precursor protein (APP) and BACE1 (β-secretase) clustering in lipid rafts, resulting in enhanced Aβ production [52]. Moreover, upregulation of neuronal cholesterol through inhibition of CYP46A1, cholesterol 24-hydroxylase which converts cholesterol to 24S-hydroxycholesterol, exacerbated AD-related pathology by inducing recruitment of APP into lipid rafts, resulting in enhanced Aβ production and cognitive deficits in an AD mouse model [53]. A number of studies have also reported a link between synucleinopathies and cholesterol, with some suggesting that cholesterol or oxidized cholesterol metabolites are responsible for increased levels of α-synuclein [54, 55], and another reporting functional impacts of cholesterol on α-synuclein, such as interactions between α-synuclein oligomers and lipid membranes [56]. Cholesterol supplementation increases α-synuclein aggregation in the detergent-insoluble fractions of cells, and this is reversed by statins [15]. These studies provide evidence that elevated cholesterol is a risk factor for neurodegenerative diseases. Our findings are consistent with these previous conclusions and further extend our knowledge by demonstrating that brain cholesterol can increase cell-to-cell propagation of aggregation-prone proteins, such as α-synuclein, and that statins alleviate these effects of cholesterol.

In conclusion, we demonstrate that statins interfere with cell-to-cell propagation of protein aggregates, alleviating behavioral and pathological deficits in animal models of synucleinopathy. We confirmed that these effects of statins are attributable to lowering cholesterol levels in the brain. Our study collectively indicates that effective delivery of statins, and perhaps other cholesterol-lowering agents, to the brain is a promising therapeutic option for treatment of PD and other neurodegenerative diseases. Our study also suggests that any therapeutic approach for neurodegenerative diseases targeting cholesterol metabolism/transport should lower cholesterol levels in the brain; systemic control of cholesterol might not be effective.

## Supplementary information

Supplementary Figure 1.

Supplementary Figure 2.

Supplementary Figure 3.

Supplementary Figure 4.

Supplementary Figure legends

Supplementary materials and methods

Supplementary Table

Original Data File

check list

## Data Availability

All data presented in the manuscript are publicly accessible at *Cell Death and Disease* website.
