# Supplementary figures and images for: Statins suppress cell-to-cell propagation of α-synuclein by lowering cholesterol
Source: Cell Death Dis. 2023 Jul 27;14(7):474. doi: 10.1038/s41419-023-05977-9 (PMC10374525; doi:10.1038/s41419-023-05977-9)

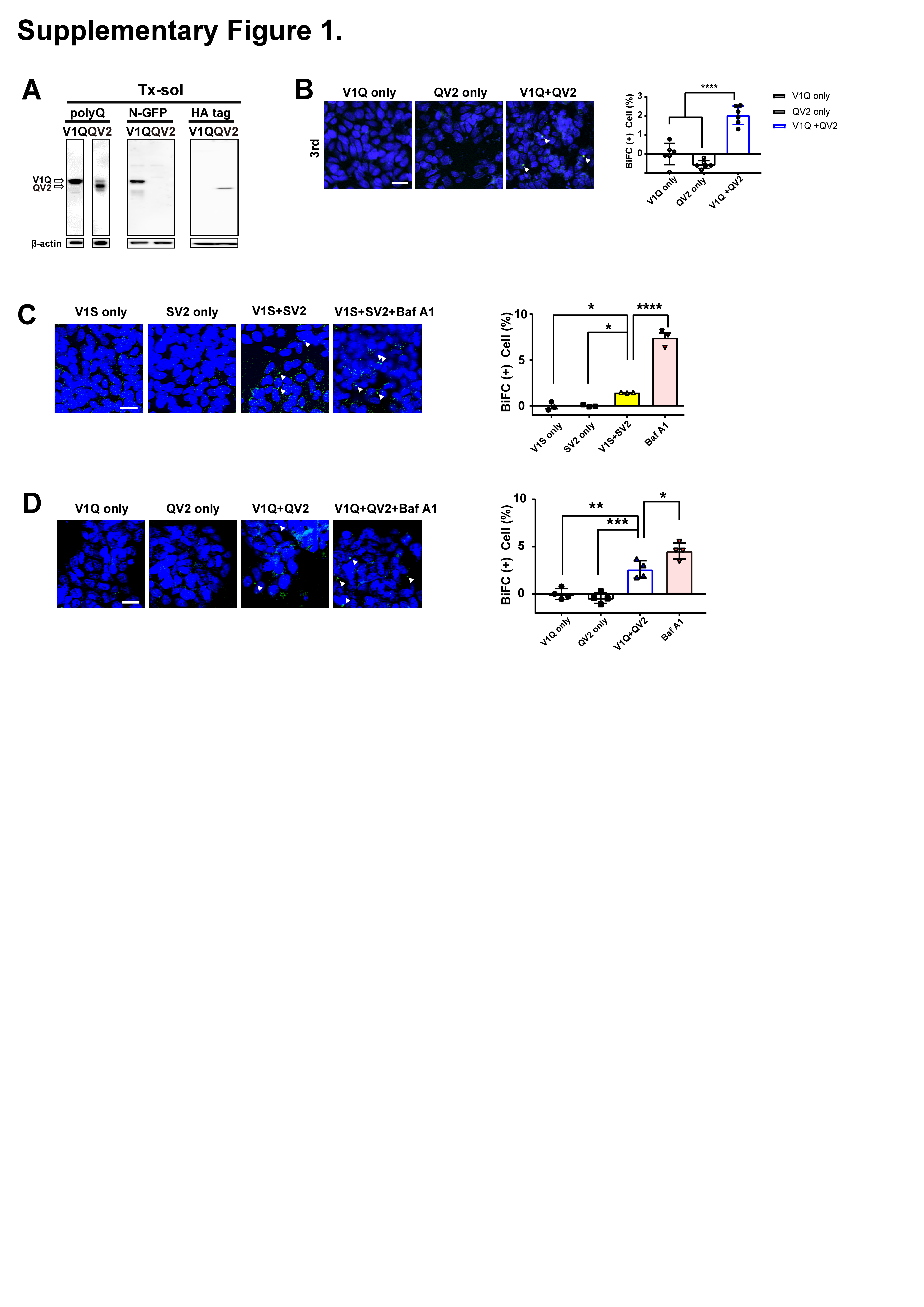

Supplement: Supplementary file 1 — Supplementary Figure 1. [file 41419_2023_5977_MOESM1_ESM.tif]

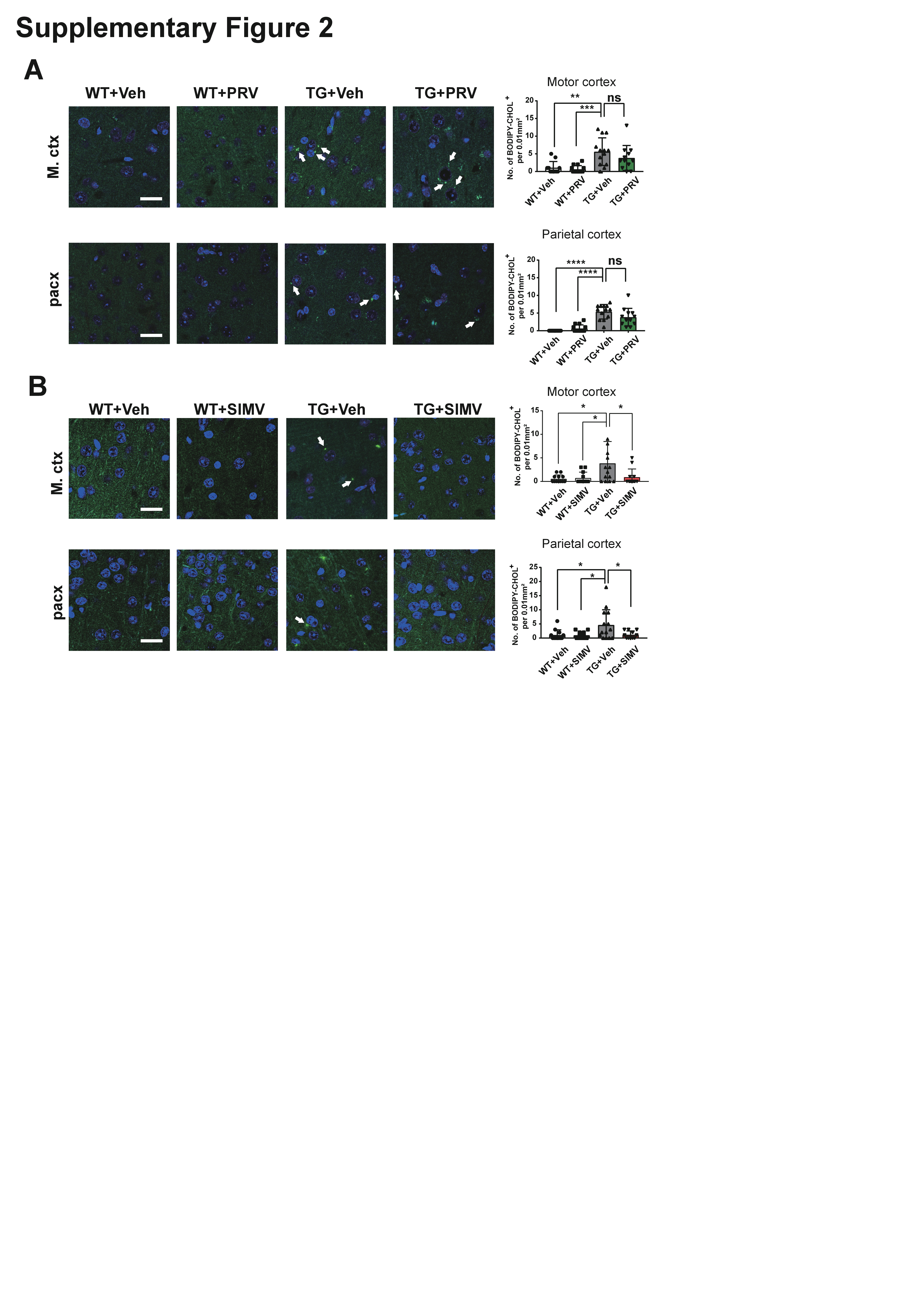

Supplement: Supplementary file 2 — Supplementary Figure 2. [file 41419_2023_5977_MOESM2_ESM.tif]

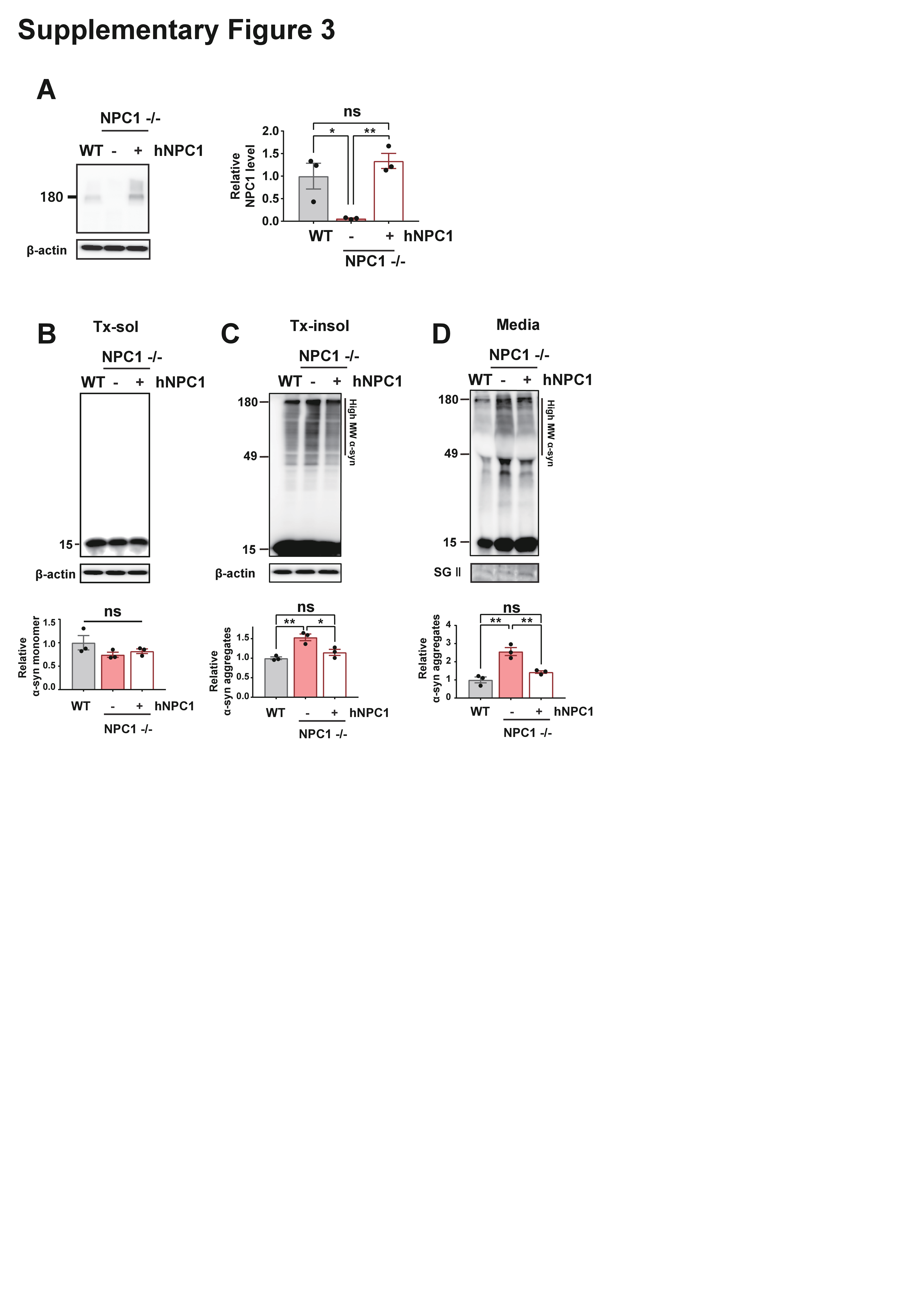

Supplement: Supplementary file 3 — Supplementary Figure 3. [file 41419_2023_5977_MOESM3_ESM.tif]

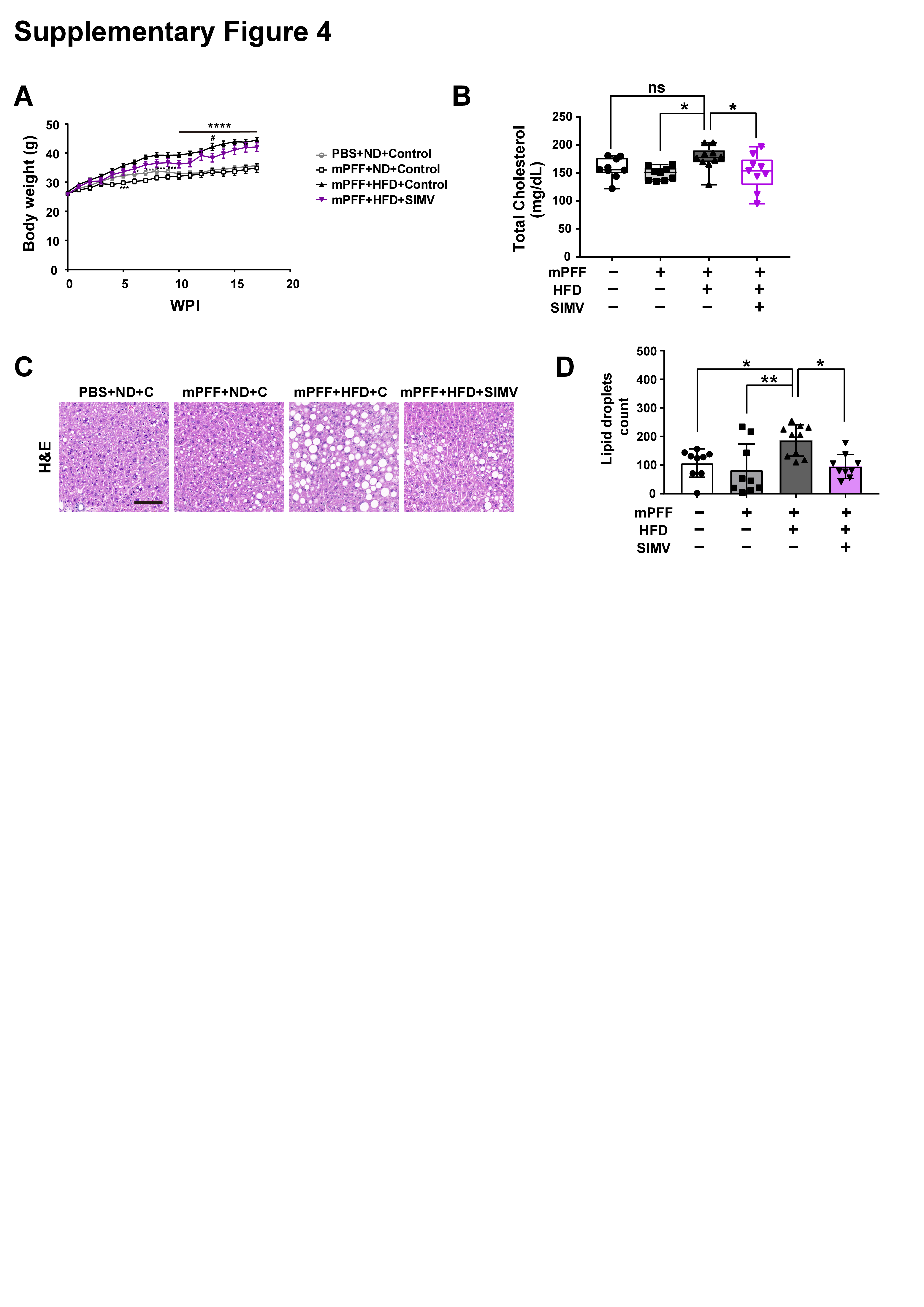

Supplement: Supplementary file 4 — Supplementary Figure 4. [file 41419_2023_5977_MOESM4_ESM.tif]
