# Supplementary material for: Statins suppress cell-to-cell propagation of α-synuclein by lowering cholesterol
Source: Cell Death Dis. 2023 Jul 27;14(7):474. doi: 10.1038/s41419-023-05977-9 (PMC10374525; doi:10.1038/s41419-023-05977-9)
Supplement: Supplementary file 5 — Supplementary Figure legends [file 41419_2023_5977_MOESM5_ESM.docx]

**Statins suppress cell-to-cell propagation of α-synuclein by lowering cholesterol**

**Joo-Ok Min^1^, Hoang-Anh Ho^2^, Wonjae Lee^1,5^, Byung Chul Jung^1,6^, Sung Jun Park^1^, Seokjoong Kim^4^, Seung-Jae Lee^1,3, *^**

^1^ Department of Biomedical Sciences, Neuroscience Research Institute, Convergence Research Center for Dementia, Seoul National University College of Medicine, Seoul 03080, Republic of Korea.

^2^ Interdisciplinary Program in Neuroscience, College of Natural Sciences, Seoul National University, Seoul, Republic of Korea.

^3^ Neuramedy Co. Ltd, Seoul, Republic of Korea.

^4^ ToolGen, Inc. Seoul, Republic of Korea.

^5^ Present address: Neuramedy Co. Ltd, Seoul, Republic of Korea.

^6^ Present address: Nutritional Sciences and Toxicology Department, University of California Berkeley, Berkeley, USA.

***Correspondence**

Seung-Jae Lee: Department of Biomedical science, Seoul National University College of Medicine, 103 Daehak-ro, Jongro-gu, Seoul 03080, Republic of Korea, Phone: 82-2-3668-7037, E-mail: sjlee66@snu.ac.kr

**Running title: Statins suppress α-synuclein propagation**

**Supplementary Figure legends**

**Supplementary Figure 1. Generation of the mHtt BiFC cell model and establishment of the HCS protocol.**

(A) Western blot analysis of mHtt-Q97 expression in V1Q and QV2 stable cell lines. The N-terminal fragment of the Venus fluorescent protein conjugated with mHtt-Q97 was confirmed using an anti-N-terminal GFP antibody (middle). Analysis of the expression of HA-tagged QV2 proteins using an anti-HA antibody (right). (B) Quantification of BiFC-positive cells in co-cultured V1Q and QV2 cells. BiFC-positive puncta (inclusions) are indicated with arrowheads. (C) BiFC-positive puncta formation in live, co-cultured cells, with or without bafilomycin A1 treatment. A total of 10^3^ cells were analyzed per experiment. (D) Summary data showing differences in the percentage of cells with puncta between co-cultured V1Q and QV2 cells and V1Q and QV2 cells alone, with and without bafilomycin A1 treatment. All data are presented as means ± SEM (*p < 0.05, **p < 0.005, ***p < 0.0005, ****p < 0.0001; one-way ANOVA test with Tukey’s post-hoc test). Scale bars: 20 μm.

**Supplementary Figure 2. Accumulation of BODIPY-Cholesterol^+^ structures in A53T TG mice and rescue by simvastatin, but not by pravastatin.**

(A) Measurement of BODIPY-Cholesterol^+^ structures in WT and TG mice administrated either vehicle or pravastatin. (B) Measurement of BODIPY-Cholesterol^+^ structures in WT and TG mice treated with vehicle or simvastatin. All data are presented as means ± SEM (*p < 0.05, **p < 0.005, ***p < 0.0005, ****p < 0.0001; one-way ANOVA with Dunnett’s post-hoc test).

**Supplementary Figure 3. Adding back human NPC1 in NPC1-KO cells reduces α-synuclein aggregation and secretion.**

(A) Western blot analysis of human NPC1 expression. (B-D) Western blot analysis of the intracellular and secreted α-synuclein. All cell experiments were repeated triplicate determination of each sample. All data are presented as means ± SEM (*p < 0.05, **p < 0.005, ***p < 0.0005, ****p < 0.0001; one-way ANOVA with Tukey’s post-hoc test). Scale bars: 20 μm.

**Supplementary Figure 4. HFD feeding increases total cholesterol levels in serum and adipocytes.**

(A) Body weights of mice injected with PBS or mα-synPFF (mPFF) and fed a normal chow diet or HFD, with or without simvastatin (SIMV) treatment. Data are presented as means ± SEM (*p < 0.05, **p < 0.005, ***p < 0.0005, ****p < 0.0001 vs. mPFF+HFD+C; ^#^p < 0.05 mPFF+HFD+C vs. mPFF+HFD+SIMV). (B) Serum levels of total cholesterol at 17 weeks post injection. (C) Hematoxylin and eosin (H&E)-stained liver tissue showing increased lipid droplets in livers of mPFF+HFD+Veh mice compared with mice on a normal chow diet and mPFF+HFD+SIMV mice. (D) Quantitative analysis of lipid droplets in the liver. Scale bar: 100 μm. All data are presented as means ± SEM (*p < 0.05, **p < 0.005, ***p < 0.0005, ****p < 0.0001; two-way ANOVA with Tukey’s post-hoc test [A], one-way ANOVA with Dunnett’s post-hoc test [B, D]).
