# Supplementary material for: Statins suppress cell-to-cell propagation of α-synuclein by lowering cholesterol
Source: Cell Death Dis. 2023 Jul 27;14(7):474. doi: 10.1038/s41419-023-05977-9 (PMC10374525; doi:10.1038/s41419-023-05977-9)
Supplement: Supplementary file 6 — Supplementary materials and methods [file 41419_2023_5977_MOESM6_ESM.docx]

**Statins suppress cell-to-cell propagation of α-synuclein by lowering cholesterol**

**Joo-Ok Min^1^, Hoang-Anh Ho^2^, Wonjae Lee^1,5^, Byung Chul Jung^1,6^, Sung Jun Park^1^, Seokjoong Kim^4^, Seung-Jae Lee^1,3, *^**

^1^ Department of Biomedical Sciences, Neuroscience Research Institute, Convergence Research Center for Dementia, Seoul National University College of Medicine, Seoul 03080, Republic of Korea.

^2^ Interdisciplinary Program in Neuroscience, College of Natural Sciences, Seoul National University, Seoul, Republic of Korea.

^3^ Neuramedy Co. Ltd, Seoul, Republic of Korea.

^4^ ToolGen, Inc. Seoul, Republic of Korea.

^5^ Present address: Neuramedy Co. Ltd, Seoul, Republic of Korea.

^6^ Present address: Nutritional Sciences and Toxicology Department, University of California Berkeley, Berkeley, USA.

***Correspondence**

Seung-Jae Lee: Department of Biomedical science, Seoul National University College of Medicine, 103 Daehak-ro, Jongro-gu, Seoul 03080, Republic of Korea, Phone: 82-2-3668-7037, E-mail: sjlee66@snu.ac.kr

**Running title: Statins suppress α-synuclein propagation**

**Supplementary Materials and Methods**

**Cell culture**

Stable cell lines were subcultured in high-glucose Dulbecco’s modified Eagle medium (DMEM; Hyclone SH30243.01) supplemented with 10% fetal bovine serum (FBS; Hyclone SH30919.03), 100 units/ml penicillin/streptomycin (Gibco GIB-15070-063) and containing 200 μg/ml G418 (Invitrogen 11811-031). Cultures were maintained at 37°C in a humidified atmosphere containing 5% CO_2_, and media were changed every 2 days. Human neuroblastoma SH-SY5Y cells (ATCC CRL-2266) were cultured in DMEM supplemented with 10% FBS (Hyclone SH30919.03) and 100 units/ml penicillin/streptomycin (Gibco GIB-15070-063) at 37°C in a humidified atmosphere containing 5% CO_2_, with media changes every 2 days. SH-SY5Y cells were differentiated by incubating with growth media (DMEM + 10% FBS + 100 units/ml penicillin/streptomycin) containing 50 μM retinoic acid (Sigma-Aldrich R2625).

**Transduction with adenovirus vectors**

Differentiated cells were co-transduced with α-synuclein adenovirus (30 M.O.I) and various adenoviruses. Adenoviral vectors were designed for overexpression of human NPC1 (0.1 M.O.I; VectorBuilder) under the control of CMV promoter and for knockdown of human SREBP2 (1 M.O.I; VectorBuilder) under the control of U6 promoter. As a control for shRNA expression experiments, a scrambled RNA vector (1 M.O.I; VectorBuilder) was used.

**Adenoviral overexpression and detergent extraction of cells**

α-Synuclein was overexpressed in differentiated SH-SY5Y cells by infecting with adenoviral constructs. After 24 hours, cells were seeded in 6-well plates and incubated with statins for 48 hours. Cells were then washed three times with cold phosphate-buffered saline (PBS) and lysed with lysis buffer containing 1% Triton X-100 (Bio-Rad; 161-0407) and 1% protease inhibitor (Sigma Aldrich P8340). Cell lysates were incubated on ice for 10 minutes and centrifuged at 16,000 × g for 10 minutes. After separating the Triton-X–soluble fraction (supernatant), the Triton-X–insoluble fraction (pellet) was resuspended in 1X Laemmli sample buffer and sonicated briefly.

**Complementary DNA (cDNA) synthesis and Reverse transcriptase polymerase chain reaction (RT-PCR)**

The total RNA from the differentiated SH-SY5Y cells (1X10^6^ cells) expressing either scramble RNA (scRNA)or human SREBP2-shRNA under the control of U6 promotor was extracted by using RNeasy Mini kit (Quiagen 74106). The RNA concentration and purity were analyzed with a NanoDrop2000 spectrophotometer at wavelengths of 260 nm/280 nm and 260 nm/230 nm (Thermo Fisher Scientific). One μg of total RNA from cells was synthesized to cDNA using iScript cDNA synthesis kit (Bio-Rad 170-8891). The cDNA was amplified by the polymerase chain reaction using ExTaq DNA polymerase (Takara RR001B). We used the following primers for RT-PCR: 5’-CGC TCC TCC ATC AAT GAC AAA-3’ forward and 5’-TCA ATG GAG TAG GGA GAG AAG C-3’ reverse primer for SREBP2; 5’-TCG TGG AAG GAC TCA TGA CC-3’ forward and 5’-CCT GCT TCA CCA CCT TCT TG-3’ reverse for GAPDH.

**Western blotting**

The efficiency of α-synuclein inhibition by statins in vitro was tested by Western blot analysis of whole-cell lysates. Briefly, 10 μg of cell-derived protein was separated by sodium dodecyl sulfate-polyacrylamide gel electrophoresis (SDS-PAGE) on 12% gels and blotted onto nitrocellulose membranes. After blocking with skim milk, membranes were incubated overnight at 4°C with the indicated primary antibodies: mouse anti-α-synuclein (Syn-1; BD Biosciences 610787, 1:1,500), mouse anti-Cathepsin D antibody (CTD-19; Abcam ab6313, 1:2,000), mouse anti-p62 antibody (BD Transduction 610832, 1:1,000), mouse anti-Lysosome-associated membrane protein 1 (LAMP1) antibody (Abcam ab25630, 1:10,000), rabbit anti-ubiquitin antibody (Chemicon ab1690, 1:1,000), rabbit anti-Niemann Pick C1 (NPC1) antibody (Abcam ab134113, 1:2,000), rabbit anti-Chromogranin C (SGⅡ) antibody (Abcam ab192824, 1:250) and β-actin (AC-15; Sigma Aldrich A5441, 1:10,000). The membranes were then washed and incubated with horseradish peroxidase-conjugated goat anti-mouse (1:3,000) and goat anti-rabbit secondary antibodies (1:3,000) (all from Bio-Rad Laboratories), after which immunoreactive proteins were detected by enhanced chemiluminescence (ECL) using Amersham ECL Prime Western Blotting substrate (Amersham RPN2232). Images were acquired using an Amersham Imager 600 (GE Healthcare Life Sciences) and analyzed with ImageJ software.

**Preparation of the mouse α-synuclein PFF (mα-synPFF) synucleinopathy model**

Recombinant α-synuclein monomer proteins were purified as previously described [1]. Fibrillated mouse α-synuclein (mα-synPFF) was collected after incubation for 5 days at 37°C and diluted into sterile Dulbecco’s phosphate-buffered saline without MgCl2 and CaCl2 (Gibco A1285601) and sonicated briefly (20% amplitude for 30 seconds, 1 second on/1 second off). Adult mice (10 weeks of age) were anesthetized with ketamine:Rompun (3.5:1; 2.5 μl/g body weight) and held in a stereotaxic instrument. After exposing and drilling a hole in the skull, 5 μg of mα-synPFF or PBS (total volume, 2.5 μl) was injected into the right striatum (AP, +1.0 mm; ML, +1.5 mm from bregma; DV, -3.0 mm) at a rate of 0.5 μl/min via a needle attached to a Hamilton syringe. Mice were monitored regularly after recovery from anesthesia and then administered simvastatin (1 mg/kg/d or 10 mg/kg/d) or vehicle (0.5% methyl cellulose) daily by oral gavage.

**Hematoxylin and eosin staining**

Paraffin-embedded liver sections (5-μm thick) were cut with a microtome and stained with hematoxylin and eosin (H&E). Sections were deparaffinized using xylene, then rehydrated with a descending graded ethanol series (100%, 95%, 80%, and 70%). Sections were subsequently incubated with hematoxylin (Vector Laboratories H-3404) and eosin (BBC Biochemical 3610) solutions, then washed in distilled water and dehydrated with a series of increasing ethanol concentrations. Dehydrated sections were mounted using a permanent mounting medium (Vector Laboratories H-5000). Lipid droplets (diameters, 20–100 μm) in adipocytes were quantified in H&E-stained sections of each group (three replicates per group) using FIJI ImageJ.

**Serum preparation and measurement of total cholesterol**

Whole blood was collected in serum-separating tubes (BD Microtainer; BD Biosciences 395967) and incubated at room temperature for 30 minutes. Serum was obtained by centrifuging samples at 1,500 × g for 10 minutes at 4°C. After centrifugation, serum was transferred to a new tube and stored at -20°C before analysis. The concentration of total cholesterol in serum was determined using a Hitachi 7180 clinical analyzer (Hitachi).

**Immunohistochemistry**

Mice were deeply anesthetized with ketamine:Rompun (3.5:1; 2.5 μl/g body weight) and perfused first with PBS and then with 4% PFA. Excised brains were post-fixed overnight in 4% PFA at 4°C. Coronal sections (40 μm) were cut with a vibratome and rinsed in PBS, after which endogenous peroxidase was quenched by incubating with 3% H_2_O_2_ (Sigma Aldrich H1009). Sections were washed with PBST and incubated in blocking solution (4% bovine serum albumin in PBST) for 1 hour at room temperature. Sections were incubated overnight at 4°C on a shaker with an rabbit anti phospho-synuclein (pS129) antibody (EP1536Y; Abcam ab51253), diluted 1:1,000. Sections were subsequently incubated with HRP-conjugated goat anti-rabbit secondary antibody (Bio-Rad #1706515, 1:200) for 90 minutes at room temperature and washed three times with PBST. Immunocomplexes were visualized using the substrate 3,3’-diaminobenzidine (DAB; Sigma Aldrich D5637) with H_2_O_2_. Sections were mounted on gelatin-coated slides using Canada balsam (Sigma Aldrich C1795). Mounted sections were imaged using a Zeiss AX10 microscope (Carl Zeiss), and optical density was analyzed using Image J software.

**Reference**

1. Jung BC, Lim YJ, Bae EJ, Lee JS, Choi MS, Lee MK*, et al.* Amplification of distinct alpha-synuclein fibril conformers through protein misfolding cyclic amplification. *Exp Mol Med*. 2017; **49**(4)**:** e314.
