# Supplementary material for: Statins suppress cell-to-cell propagation of α-synuclein by lowering cholesterol
Source: Cell Death Dis. 2023 Jul 27;14(7):474. doi: 10.1038/s41419-023-05977-9 (PMC10374525; doi:10.1038/s41419-023-05977-9)
Supplement: Supplementary file 7 — Supplementary Table [file 41419_2023_5977_MOESM7_ESM.pdf]

Statins suppress cell-to-cell propagation of α-synuclein by lowering cholesterol

Joo-Ok Min[1], Hoang-Anh Ho[2], Wonjae Lee[1,5], Byung Chul Jung[1,6], Sung Jun Park[1], Seokjoong Kim[4], Seung-Jae Lee[1,3, *]

[1] Department of Biomedical Sciences, Neuroscience Research Institute, Convergence Research Center for Dementia, Seoul National University College of Medicine, Seoul 03080, Republic of Korea.

[2] Interdisciplinary Program in Neuroscience, College of Natural Sciences, Seoul National University, Seoul, Republic of Korea.

[3] Neuramedy Co. Ltd, Seoul, Republic of Korea.

[4] ToolGen, Inc., Seoul, Republic of Korea.

[5] Present address: Neuramedy Co. Ltd, Seoul, Republic of Korea.

[6] Present address: Nutritional Sciences and Toxicology Department, University of California Berkeley, Berkeley, USA.

***Correspondence**

Seung-Jae Lee: Department of Biomedical science, Seoul National University College of Medicine, 103 Daehak-ro, Jongro-gu, Seoul 03080, Republic of Korea, Phone: 82-2-3668-7037, E-mail: sjlee66@snu.ac.kr

Supplementary Table 1. Primary high-contents drug screening results in α-synuclein and mHtt BiFC cell models

HCS No.	Name	Normalized inhibition %	
		α-syn	mHtt
01A02	Azaguanine-8	43.95738 ± 3.27826	-338.45081 ± 218.57731
01A03	Allantoin	44.95738 ± 21.58770	-71.22844 ± 11.32971
01A04	Acetazolamide	84.45766 ± 8.50177	-563.40765 ± 50.57346
01A05	Metformin hydrochloride	87.87698 ± 11.43418	-674.45758 ± 58.95143
01A06	Atracurium besylate	-118.82002 ± 79.41308	-167.59416 ± 20.24039
01A07	Isoflupredone acetate	33.17009 ± 19.05572	-191.55353 ± 20.04717
01A08	Amiloride hydrochloride dihydrate	19.64792 ± 11.92146	-117.43316 ± 18.53687
01A09	Amprolium hydrochloride	93.53471 ± 20.38008	-243.62278 ± 39.09490
01A10	Hydrochlorothiazide	75.95594 ± 27.57995	-166.42997 ± 35.60086
01A11	Sulfaguanidine	71.48545 ± 4.59237	-156.17291 ± 34.24707
01B02	Meticrane	64.69833 ± 4.64100	-246.46638 ± 88.18927
01B03	Benzonatate	60.56317 ± 14.06341	-465.88781 ± 65.55744
01B04	Hydroflumethiazide	30.51627 ± 22.03032	-397.92887 ± 40.49511
01B05	Sulfacetamide sodic hydrate	53.57044 ± 18.61152	-419.80289 ± 41.64164
01B06	Heptaminol hydrochloride	-52.10899 ± 17.87634	-69.40044 ± 8.62705
01B07	Sulfathiazole	-153.47026 ± 123.19257	-109.03362 ± 5.63410
01B08	Levodopa	-40.00393 ± 94.22208	-86.87612 ± 7.80569
01B09	Idoxuridine	71.25550 ± 5.42556	-106.95800 ± 24.01866
01B10	Captopril	77.76200 ± 8.10988	-123.14588 ± 23.21438
01B11	Minoxidil	62.55058 ± 22.88106	-93.16901 ± 12.02168
01C02	Sulfaphenazole	46.41324 ± 15.22760	-167.64041 ± 27.63118
01C03	Panthenol (D)	38.80931 ± 19.50363	-381.90528 ± 75.83726
01C04	Sulfadiazine	17.15296 ± 5.57119	-252.05197 ± 16.28783
01C05	Norethynodrel	19.56222 ± 29.39051	-265.38270 ± 32.82612
01C06	Thiamphenicol	-51.54879 ± 54.91382	-46.36425 ± 5.40851
01C07	Cimetidine	-31.35655 ± 11.86376	-104.80880 ± 18.51670
01C08	Doxylamine succinate	80.43778 ± 29.39051	-64.07523 ± 18.66730
01C09	Ethambutol dihydrochloride	76.51881 ± 3.22332	-127.17540 ± 42.15001
01C10	Antipyrine	13.35248 ± 10.08788	-98.79708 ± 24.75992
01C11	Antipyrine, 4-hydroxy	14.65183 ± 33.73589	-51.27079 ± 19.91019
01D02	Chloramphenicol	36.38004 ± 11.66132	-7.96284 ± 41.25454
01D03	Epirizole	38.85283 ± 19.82938	-158.47966 ± 66.14874
01D04	Diprophylline	38.12498 ± 3.22441	-115.12455 ± 10.29058
01D05	Triamterene	25.25804 ± 31.78912	-134.86733 ± 53.07975
01D06	Dapsone	-26.15734 ± 15.78383	-26.12186 ± 11.62555
01D07	Troleandomycin	-20.88508 ± 22.48976	-51.92186 ± 3.92970
01D08	Pyrimethamine	-108.01150 ± 38.37251	-39.97359 ± 38.84989
01D09	Hexamethonium dibromide dihydrate	71.49924 ± 12.78751	-82.69735 ± 35.70254
01D10	Diflunisal	-235.831031 ± 34.215805	-41.51443 ± 4.54055
01D11	Niclosamide	70.61194 ± 13.23094	-46.56521 ± 20.06980
01E02	Procaine hydrochloride	31.46614 ± 20.50603	14.85652 ± 17.38075
01E03	Moxisylyte hydrochoride	25.10176 ± 20.44727	-43.60682 ± 16.64204
01E04	Betazole hydrochloride	-2.04215 ± 19.60609	1.17773 ± 6.24009
01E05	Isoxicam	38.61040 ± 8.00961	-104.43190 ± 33.51385
01E06	(S)-Naproxen	-37.75693 ± 32.91420	6.33798 ± 9.68470
01E07	Naphazoline hydrochloride	5.91231 ± 13.96516	-41.50470 ± 12.23868

01E08	Ticlopidine hydrochloride	-200.51515 ± 102.86614	-5.60322 ± 10.62666
01E09	Dicyclomine hydrochloride	56.07272 ± 14.11470	-19.17582 ± 31.79294
01E10	Amyleine hydrochloride	62.55012 ± 16.75608	-5.06202 ± 2.75495
01E11	Lidocaine hydrochloride	-67.74917 ± 19.62669	2.21055 ± 15.75517
01F02	Mupirocin	43.30846 ± 7.02706	38.74483 ± 27.14728
01F03	Carbamazepine	26.39547 ± 14.06367	21.20792 ± 30.80027
01F04	Triflupromazine hydrochloride	9.60509 ± 38.40946	15.14133 ± 1.18219
01F05	Mefenamic acid	42.07057 ± 24.76143	-40.78421 ± 19.09482
01F06	Acetohexamide	-32.69614 ± 18.44632	29.25818 ± 17.13411
01F07	Sulpiride	-17.86392 ± 27.75850	-2.80571 ± 12.57773
01F08	Benoxinate hydrochloride	1.79102 ± 47.77717	3.10786 ± 7.70823
01F09	Oxethazaine	60.22707 ± 4.88591	-50.84395 ± 78.76030
01F10	Pheniramine maleate	12.35595 ± 24.96726	32.85143 ± 11.03910
01F11	Tolazoline hydrochloride	31.53783 ± 14.58003	42.79431 ± 6.35467
01G02	Morantel tartrate	-1.53944 ± 18.67500	88.86500 ± 12.68862
01G03	Homatropine hydrobromide (R,S)	43.98915 ± 12.27607	34.96275 ± 25.96836
01G04	Nifedipine	-13.18893 ± 22.11333	79.92640 ± 7.97580
01G05	Chlorpromazine hydrochloride	-263.12518 ± 93.04021	-1.06831 ± 26.10089
01G06	Diphenhydramine hydrochloride	-41.46582 ± 19.37653	4.24793 ± 7.23401
01G07	Minaprine dihydrochloride	-29.55190 ± 10.53444	2.16504 ± 3.06259
01G08	Miconazole	-180.82691 ± 23.65488	14.83334 ± 9.28231
01G09	Isoxsuprine hydrochloride	40.05723 ± 17.29117	-18.65218 ± 42.27145
01G10	Acebutolol hydrochloride	-14.48088 ± 3.70614	12.43868 ± 28.20479
01G11	Tolnaftate	32.33721 ± 11.05397	39.95618 ± 7.69389
01H02	Todralazine hydrochloride	3.04376 ± 24.83362	41.74321 ± 28.16882
01H03	Imipramine hydrochloride	34.98558 ± 23.58782	77.91846 ± 4.94778
01H04	Sulindac	26.34808 ± 8.78909	40.98890 ± 31.36776
01H05	Amitryptiline hydrochloride	-14.43597 ± 18.79959	-44.94800 ± 37.29826
01H06	Adiphenine hydrochloride	24.92191 ± 16.92448	17.71549 ± 11.63621
01H07	Dibucaine	-12.46241 ± 17.32741	21.72113 ± 38.20956
01H08	Prednisone	0.16417 ± 2.82224	22.47546 ± 17.44934
01H09	Thioridazine hydrochloride	70.49308 ± 23.96339	6.85550 ± 71.89896
01H10	Diphemanil methylsulfate	-0.99808 ± 24.84644	36.11560 ± 14.25647
01H11	Trimethobenzamide hydrochloride	-201.24436 ± 172.21025	18.00856 ± 10.74209
02A02	Metronidazole	124.20320 ± 24.20320	-143.74251 ± 15.04402
02A03	Fulvestrant	70.34252 ± 31.96035	-64.02460 ± 13.09184
02A04	Edrophonium chloride	12.29983 ± 29.20183	-53.88552 ± 31.85549
02A05	Moroxidine hydrochloride	111.76955 ± 38.18402	-51.44386 ± 7.38587
02A06	Baclofen (R,S)	-17.59005 ± 5.24148	-566.61716 ± 144.73011
02A07	Acyclovir	99.46423 ± 3.10624	-725.61597 ± 56.11510
02A08	Diazoxide	77.71394 ± 3.06488	-626.12310 ± 38.88770
02A09	Amidopyrine	35.74223 ± 6.91463	-458.41869 ± 41.93243
02A10	Busulfan	13.10938 ± 23.10703	-413.77491 ± 116.07109
02A11	Pindolol	46.85872 ± 20.93210	-532.99560 ± 35.31650
02B02	Khellin	58.24265 ± 25.80719	-130.33686 ± 13.03504
02B03	Zimelidine dihydrochloride monohydrate	57.70698 ± 22.88845	-8.07103 ± 18.20205
02B04	Azacyclonol	57.59893 ± 51.62905	-13.35113 ± 18.19688
02B05	Azathioprine	60.43246 ± 43.29729	18.07673 ± 7.19471
02B06	Lynestrenol	39.04852 ± 2.86942	-111.63799 ± 54.97072
02B07	Guanabenz acetate	74.17153 ± 8.15969	-345.56939 ± 40.91216
02B08	Disulfiram	-3.97280 ± 29.37551	-365.89479 ± 57.23743

02B09	Acetylsalicylsalicylic acid	66.36345 ± 5.32707	-439.16530 ± 18.06078
02B10	Mianserine hydrochloride	41.39149 ± 23.06992	-27.18130 ± 24.86985
02B11	Nocodazole	-59.51880 ± 55.25074	-646.31303 ± 138.66081
02C02	R(-) Apomorphine hydrochloride hemihydrate	-537.21173 ± 257.82771	-88.61993 ± 15.83828
02C03	Amoxapine	-381.93401 ± 266.59538	25.20759 ± 11.34658
02C04	Cyproheptadine hydrochloride	2.51335 ± 49.40213	9.72823 ± 22.41733
02C05	Famotidine	-21.35994 ± 17.81253	14.13945 ± 14.08751
02C06	Danazol	-7.61157 ± 13.74273	-203.88283 ± 87.00401
02C07	Nicorandil	69.99493 ± 35.05585	-244.62222 ± 32.06750
02C08	Pioglitazone	99.14107 ± 4.08607	-271.66333 ± 30.11397
02C09	Nomifensine maleate	54.12215 ± 14.68037	-129.91388 ± 25.82776
02C10	Aliskiren hemifumarate	48.09596 ± 0.31118	-110.15764 ± 30.30764
02C11	Oxandrolone	66.65760 ± 7.13843	-88.41217 ± 59.64917
02D02	Naloxone hydrochloride	105.20625 ± 31.15740	-36.79605 ± 23.91031
02D03	Metolazone	33.01832 ± 18.70919	64.45735 ± 6.32425
02D04	Ciprofloxacin hydrochloride monohydrate	116.45263 ± 48.83090	30.76450 ± 11.80387
02D05	Ampicillin trihydrate	68.03993 ± 51.63066	39.27657 ± 12.24389
02D06	Haloperidol	9.13066 ± 9.33582	12.96752 ± 57.28199
02D07	Naltrexone hydrochloride dihydrate	40.93942 ± 21.31525	-60.37011 ± 18.80606
02D08	Chlorpheniramine maleate	47.22554 ± 5.29189	-129.69834 ± 17.90307
02D09	Nalbuphine hydrochloride	61.03251 ± 7.43588	-144.55844 ± 47.09628
02D10	Picotamide monohydrate	29.84599 ± 5.18279	113.58599 ± 21.91628
02D11	Triamcinolone	27.85382 ± 13.85275	92.94171 ± 24.45020
02E02	Bromocryptine mesylate	56.36338 ± 14.56696	19.82221 ± 9.62580
02E03	Amfepramone hydrochloride	-36.87406 ± 49.85974	51.26161 ± 4.30210
02E04	Dehydrocholic acid	48.65141 ± 7.14370	57.85859 ± 6.97438
02E05	Tioconazole	-72.03514 ± 20.21558	75.80316 ± 6.68616
02E06	Perphenazine	32.63035 ± 16.30181	99.23933 ± 36.36719
02E07	Mefloquine hydrochloride	-82.04928 ± 39.28640	-53.10607 ± 24.16345
02E08	Isoconazole	1.47170 ± 30.74022	-13.63060 ± 19.25695
02E09	Spironolactone	53.84625 ± 9.94625	-455.26689 ± 125.67453
02E10	Pirenzepine dihydrochloride	14.01929 ± 3.82781	82.78451 ± 18.27620
02E11	Dexamethasone acetate	45.15984 ± 11.32883	119.87557 ± 23.59360
02F02	Glipizide	-26.84706 ± 27.52589	-91.00960 ± 48.27152
02F03	Loxapine succinate	42.31734 ± 32.64359	65.84648 ± 21.20217
02F04	Hydroxyzine dihydrochloride	37.72205 ± 21.06955	67.04389 ± 5.78770
02F05	Diltiazem hydrochloride	28.08634 ± 40.56858	58.77144 ± 24.33607
02F06	Azilsartan kamedoxomil	-31.59278 ± 16.59298	143.34332 ± 57.67677
02F07	Astemizole	23.49028 ± 12.30414	108.54937 ± 13.13434
02F08	Clindamycin hydrochloride	53.10075 ± 14.18007	55.87097 ± 29.83685
02F09	Terfenadine	-625.26240 ± 306.37429	-371.78373 ± 36.89151
02F10	Cefotaxime sodium salt	28.84414 ± 7.18206	44.17323 ± 25.66973
02F11	Tetracycline hydrochloride	-5.00441 ± 16.99304	39.11212 ± 24.93397
02G02	Verapamil hydrochloride	73.47994 ± 53.42695	-0.77251 ± 19.04061
02G03	Dipyridamole	-938.29173 ± 201.77159	-49.67711 ± 17.70968
02G04	Chlorhexidine	40.13538 ± 69.23203	45.64042 ± 24.42767
02G05	Loperamide hydrochloride	22.51224 ± 24.12556	64.46519 ± 13.81731
02G06	Chlortetracycline hydrochloride	11.22049 ± 17.06784	17.81729 ± 0.89343
02G07	Tamoxifen citrate	43.91531 ± 21.00036	100.89773 ± 53.89061
02G08	Nicergoline	40.49597 ± 19.69463	155.81631 ± 11.60036
02G09	Canrenoic acid potassium salt	33.30642 ± 15.08833	-140.86703 ± 24.33848

02G10	Thioproperazine dimesylate	42.22837 ± 12.53120	18.41105 ± 1.63873
02G11	Dihydroergotamine tartrate	59.48307 ± 7.32714	72.62329 ± 41.21740
02H02	Erythromycin	74.38259 ± 37.19181	29.36861 ± 1.28260
02H03	Chloroxine	-78.13820 ± 50.76651	44.37272 ± 31.19888
02H04	Didanosine	49.83723 ± 27.06545	98.55718 ± 4.65799
02H05	Josamycin	36.78496 ± 20.98575	40.89013 ± 28.00813
02H06	Paclitaxel	-330.35262 ± 71.22792	19.38362 ± 2.48364
02H07	Ivermectin	16.31098 ± 29.60230	118.25199 ± 82.94163
02H08	Gallamine triethiodide	50.85449 ± 4.01810	18.02138 ± 1.23551
02H09	Neomycin sulfate	63.73375 ± 3.52609	-43.99988 ± 35.69976
02H10	Dihydrostreptomycin sulfate	66.70441 ± 9.00097	106.32538 ± 2.81033
02H11	Gentamicine sulfate	60.80565 ± 18.51447	102.05005 ± 20.50447
03A02	Isoniazid	10.96255 ± 39.93377	-423.22784 ± 51.45972
03A03	Pentylenetetrazole	7.68139 ± 30.46546	-602.98054 ± 225.64166
03A04	Chlorzoxazone	22.44345 ± 29.80079	-129.02560 ± 10.05953
03A05	Ornidazole	-7.66987 ± 50.77657	-793.37770 ± 58.13859
03A06	Ethosuximide	124.80638 ± 10.42522	40.83064 ± 5.01581
03A07	Mafenide hydrochloride	25.31680 ± 6.11268	22.76249 ± 5.05466
03A08	Riluzole hydrochloride	39.82941 ± 7.51590	33.21155 ± 6.27399
03A09	Nitrofurantoin	-11.32962 ± 8.36183	35.17848 ± 2.74458
03A10	Hydralazine hydrochloride	24.65930 ± 2.23077	31.47903 ± 2.32807
03A11	Phenelzine sulfate	17.01806 ± 36.89711	28.75356 ± 1.46136
03B02	Tranexamic acid	60.30625 ± 25.88560	-198.64620 ± 46.27911
03B03	Etofylline	80.25877 ± 43.48207	69.40257 ± 6.28289
03B04	Tranylcypromine hydrochloride	43.23712 ± 16.32993	24.58104 ± 2.31952
03B05	Alverine citrate salt	-254.01820 ± 333.25943	23.86152 ± 5.25433
03B06	Aceclofenac	38.64018 ± 47.48308	31.46883 ± 0.93743
03B07	Iproniazide phosphate	25.85259 ± 11.67640	73.83320 ± 31.04959
03B08	Sulfamethoxazole	37.10831 ± 28.02671	43.76065 ± 22.93009
03B09	Mephenesin	11.53711 ± 11.96769	22.25191 ± 2.33529
03B10	Phenformin hydrochloride	22.66781 ± 10.90694	36.34083 ± 39.77207
03B11	Flutamide	8.57006 ± 27.05034	61.84529 ± 26.59338
03C02	Ampyrone	83.48238 ± 13.19699	-140.86703 ± 24.33848
03C03	Levamisole hydrochloride	85.85788 ± 24.41492	82.75769 ± 6.34401
03C04	Pargyline hydrochloride	61.19604 ± 33.74360	138.09792 ± 31.00409
03C05	Methocarbamol	137.74143 ± 47.44411	43.22886 ± 2.85954
03C06	Aztreonam	-10.92171 ± 18.70713	27.40160 ± 11.78861
03C07	Cloxacillin sodium salt	49.80585 ± 14.05014	73.60388 ± 26.57742
03C08	Lacosamide	24.83456 ± 7.34514	166.63515 ± 29.02423
03C09	Pentolinium bitartrate	45.15244 ± 16.17132	107.93017 ± 44.94084
03C10	Aminopurine, 6-benzyl	-32.92309 ± 38.50696	56.76009 ± 27.26839
03C11	Tolbutamide	68.28947 ± 17.53732	80.30799 ± 0.67549
03D02	Midodrine hydrochloride	34.78777 ± 39.16267	-164.36906 ± 81.56027
03D03	Thalidomide	-4.54367 ± 37.96959	26.75834 ± 18.35210
03D04	Oxolinic acid	-7.38101 ± 19.20949	40.59918 ± 18.08457
03D05	Nimesulide	44.40775 ± 19.68739	29.61259 ± 28.92506
03D06	Asenapine maleate	57.16178 ± 13.72380	76.69213 ± 42.42750
03D07	Pentoxifylline	-16.79185 ± 8.03786	1.03476 ± 10.44044
03D08	Metaraminol bitartrate	-1.71683 ± 11.03460	47.96047 ± 26.39260
03D09	Salbutamol	6.45595 ± 1.96280	33.39384 ± 6.48605
03D10	Prilocaine hydrochloride	-5.95432 ± 26.25565	-14.21083 ± 20.99192

03D11	Camptothecine (S,+)	-853.47849 ± 181.04904	46.38143 ± 12.89336
03E02	Ranitidine hydrochloride	50.90717 ± 5.42219	-60.80140 ± 80.98893
03E03	Tiratricol, 3,3',5-triiodothyroacetic acid	-10.14688 ± 54.42833	22.58651 ± 49.85921
03E04	Flufenamic acid	-14.31968 ± 13.39580	25.61228 ± 3.33330
03E05	Flumequine	50.34057 ± 23.36101	11.62250 ± 13.92922
03E06	Tolfenamic acid	35.81994 ± 28.24598	28.369837 ± 7.758074
03E07	Meclofenamic acid sodium salt monohydrate	-19.49866 ± 8.66227	47.91447 ± 12.23945
03E08	Tibolone	-34.08483 ± 9.53383	32.27634 ± 12.13406
03E09	Trimethoprim	-10.74822 ± 13.89481	49.59547 ± 19.06618
03E10	Metoclopramide monohydrochloride	69.96458 ± 8.42340	7.30289 ± 10.50907
03E11	Fenbendazole	-658.49927 ± 303.92088	7.43582 ± 79.27730
03F02	Piroxicam	26.30464 ± 24.22924	43.05826 ± 37.99376
03F03	Pyrantel tartrate	2.69596 ± 89.38314	-26.71962 ± 30.10353
03F04	Fenspiride hydrochloride	-81.29050 ± 50.40242	-9.11499 ± 21.00471
03F05	Gemfibrozil	-46.23319 ± 36.26268	-15.89121 ± 17.94754
03F06	Mefexamide hydrochloride	47.23408 ± 8.84528	17.16619 ± 2.54152
03F07	Tiapride hydrochloride	-3.99795 ± 5.86704	-9.62971 ± 33.00606
03F08	Mebendazole	-281.65420 ± 59.97868	-2.30285 ± 5.73917
03F09	Fenbufen	-12.48049 ± 5.53316	17.87377 ± 5.57400
03F10	Ketoprofen	10.54922 ± 10.91744	-426.29732 ± 76.06919
03F11	Indapamide	-9.01549 ± 44.59260	-84.03617 ± 104.45910
03G02	Norfloxacin	85.07249 ± 8.85725	53.34772 ± 18.87111
03G03	Antimycin A	-110.41925 ± 46.22853	-80.49457 ± 18.47000
03G04	Xylometazoline hydrochloride	16.20409 ± 6.18789	4.67121± 22.69750
03G05	Oxymetazoline hydrochloride	3.12116 ± 11.06882	-56.15228 ± 3.67651
03G06	Nifenazone	52.27941 ± 9.47494	65.76636 ± 16.51007
03G07	Griseofulvin	-80.90688 ± 18.49119	14.12459 ± 11.21132
03G08	Clemizole hydrochloride	-0.24358 ± 18.09291	87.45886 ± 95.10977
03G09	Tropicamide	-5.45419 ± 25.75860	-1.91743 ± 11.17176
03G10	Nefopam hydrochloride	25.31898 ± 16.23434	-311.81545 ± 66.22305
03G11	Phentolamine hydrochloride	-17.99614 ± 41.62819	-73.39088 ± 164.40882
03H02	Etodolac	50.26645 ± 14.95337	63.14930 ± 43.21410
03H03	Scopolamin-N-oxide hydrobromide	-44.59478 ± 114.04724	-24.08418 ± 24.34997
03H04	Hyoscyamine (L)	-36.26854 ± 33.90194	-18.15151 ± 27.96396
03H05	Chlorphensin carbamate	-8.62057 ± 20.98735	-55.94019 ± 10.15208
03H06	Carmofur	-95.33764 ± 22.00208	47.08019 ± 20.67718
03H07	Dilazep dihydrochloride	-25.82761 ± 31.26488	-18.55766 ± 15.18468
03H08	Ofloxacin	10.21938 ± 15.11873	6.81238 ± 40.90027
03H09	Lomefloxacin hydrochloride	-35.71683 ± 22.42041	-38.80362 ± 10.54370
03H10	Orphenadrine hydrochloride	125.82761 ± 31.26488	-571.27485 ± 50.54051
03H11	Proglumide	12.34171 ± 35.28460	-293.69855 ± 117.76591
04A02	Mexiletine hydrochloride	-1.03697 ± 11.89208	22.66368 ± 3.49497
04A03	Flavoxate hydrochloride	-18.29955 ± 14.89274	27.85839 ± 11.75533
04A04	Bufexamac	-34.17565 ± 35.25515	17.80211 ± 7.23769
04A05	Glutethimide, para-amino	131.93612 ± 17.51734	42.23451 ± 25.46224
04A06	Dropropizine (R,S)	71.99165 ± 25.88646	58.74118 ± 6.45316
04A07	Pinacidil	-70.68745 ± 17.17363	18.94368 ± 7.35205
04A08	Albendazole	-342.90841 ± 386.82737	-64.88941 ± 21.03989
04A09	Clonidine hydrochloride	56.59109 ± 7.61553	-12.07084 ± 31.55362
04A10	Bupropion hydrochloride	54.17092 ± 7.67267	-5.19289 ± 59.53900
04A11	Alprenolol hydrochloride	54.50525 ± 4.53165	42.68206 ± 8.11233

04B02	Chlorothiazide	-13.20583 ± 5.23900	65.30193 ± 27.38557
04B03	Diphenidol hydrochloride	-9.29980 ± 1.95083	15.62717 ± 3.90714
04B04	Norethindrone	7.05139 ± 14.11600	49.77903 ± 7.18057
04B05	Nortriptyline hydrochloride	-65.69637 ± 18.94957	48.44838 ± 40.70190
04B06	Niflumic acid	5.79146 ± 20.64074	71.93302 ± 18.14026
04B07	Isotretinoin	-122.74194 ± 37.85609	54.96537 ± 5.82644
04B08	Retinoic acid	55.36209 ± 38.28605	63.77801 ± 11.04232
04B09	Antazoline hydrochloride	72.91535 ± 3.57163	41.85033 ± 25.04300
04B10	Ethacrynic acid	60.11173 ± 5.26076	-15.69891 ± 34.62061
04B11	Praziquantel	57.62234 ± 6.29051	27.75987 ± 20.15088
04C02	Ethisterone	51.66364 ± 9.66936	66.55387 ± 11.35482
04C03	Triprolidine hydrochloride	52.91383 ± 8.82245	23.67767 ± 22.87673
04C04	Doxepin hydrochloride	73.16476 ± 2.02630	27.57619 ± 42.92899
04C05	Dyclonine hydrochloride	-107.95656 ± 79.47073	54.52775 ± 22.47681
04C06	Dimenhydrinate	-114.86725 ± 13.24080	40.37423 ± 28.12501
04C07	Disopyramide	-146.46352 ± 15.91357	58.40319 ± 11.89590
04C08	Clotrimazole	-192.21112 ± 8.52085	91.16083 ± 12.47946
04C09	Vinpocetine	81.35482 ± 3.44971	62.94828 ± 7.54248
04C10	Clomipramine hydrochloride	59.37117 ± 8.00028	38.47063 ± 42.00148
04C11	Fendiline hydrochloride	67.77477 ± 4.53878	-2.40575 ± 4.47513
04D02	Vincamine	-24.66366 ± 7.02633	58.38096 ± 9.54059
04D03	Indomethacin	-5.21454 ± 9.05015	23.27781 ± 36.76339
04D04	Cortisone	-5.56217 ± 1.19880	-87.78579 ± 18.88698
04D05	Prednisolone	-66.78936 ± 34.69070	-6.81416 ± 9.03114
04D06	Fenofibrate	-117.67121 ± 32.58706	-33.82102 ± 13.75232
04D07	Bumetanide	-179.69043 ± 44.49968	102.21636 ± 23.67710
04D08	Labetalol hydrochloride	-2.23433 ± 22.76803	63.50801 ± 27.66075
04D09	Cinnarizine	77.06957 ± 2.92691	66.71424 ± 9.74040
04D10	Methylprednisolone, 6-alpha	71.46510 ± 13.46853	43.08995 ± 17.56820
04D11	Quinidine hydrochloride monohydrate	66.20329 ± 3.44644	-15.16439 ± 4.71463
04E02	Fludrocortisone acetate	-88.58595 ± 33.50560	-3.39953 ± 43.43685
04E03	Fenoterol hydrobromide	-47.17482 ± 22.66986	12.24587 ± 0.97740
04E04	Homochlorcyclizine dihydrochloride	-596.32011 ± 99.88951	-72.49480 ± 24.23703
04E05	Diethylcarbamazine citrate	-46.30624 ± 30.82661	-57.90631 ± 16.04532
04E06	Chenodiol	-125.49205 ± 24.49901	-50.48095 ± 35.69572
04E07	Perhexiline maleate	-145.09836 ± 64.92694	118.22737 ± 9.23110
04E08	Oxybutynin chloride	-58.87776 ± 41.99585	95.07433 ± 7.06623
04E09	Spiperone	76.09940 ± 4.93709	55.83423 ± 12.84999
04E10	Pyrilamine maleate	66.21644 ± 6.07329	40.09274 ± 8.53844
04E11	Sulfinpyrazone	65.16249 ± 7.24220	-6.61832 ± 21.30788
04F02	Dantrolene sodium salt	-111.66088 ± 15.72538	-161.61668 ± 101.25761
04F03	Trazodone hydrochloride	-22.02293 ± 20.49531	172.87569 ± 25.48419
04F04	Glafenine hydrochloride	-39.32648 ± 8.85269	-97.20138 ± 18.52796
04F05	Pimethixene maleate	-40.86942 ± 15.16943	-76.16738 ± 30.17753
04F06	Pergolide mesylate	-61.15073 ± 25.83985	-51.48311 ± 5.75600
04F07	Acemetacin	-105.76626 ± 58.82138	98.26435 ± 23.22517
04F08	Benzydamine hydrochloride	-72.28843 ± 55.74831	59.66181 ± 7.81580
04F09	Fipexide hydrochloride	71.47145 ± 5.40412	49.15124 ± 12.87514
04F10	Mifepristone	81.99112 ± 9.15294	14.45946 ± 20.44810
04F11	Diperodon hydrochloride	57.77378 ± 2.68780	-3.53086 ± 18.13921
04G02	Lisinopril	-76.65305 ± 15.68872	-53.42707 ± 4.24548

04G03	Lincomycin hydrochloride	10.51545 ± 17.88321	19.47523 ± 31.31646
04G04	Telenzepine dihydrochloride	-20.07635 ± 16.31374	-105.50545 ± 30.43519
04G05	Econazole nitrate	-102.61775 ± 83.40056	-64.48992 ± 101.71925
04G06	Bupivacaine hydrochloride	-66.76072 ± 59.37813	-138.10484 ± 7.91331
04G07	Clemastine fumarate	-237.67178 ± 57.83497	106.19376 ± 10.40510
04G08	Oxytetracycline dihydrate	69.62767 ± 6.38047	105.82296 ± 5.61277
04G09	Pimozide	3.60037 ± 18.06482	103.25715 ± 21.88648
04G10	Amodiaquin dihydrochloride dihydrate	81.79238 ± 7.94217	-24.21460 ± 14.59729
04G11	Mebeverine hydrochloride	64.12345 ± 9.02913	-55.09784 ± 6.53764
04H02	Ifenprodil tartrate	-17.03898 ± 12.30245	-658.25854± 71.48468
04H03	Flunarizine dihydrochloride	43.43961 ± 41.47154	-3.45049 ± 21.37779
04H04	Trifluoperazine dihydrochloride	44.13057 ± 12.74481	-184.17395 ± 12.24423
04H05	Enalapril maleate	-60.75110 ± 54.27129	-90.44513 ± 91.12956
04H06	Minocycline hydrochloride	-13.12546 ± 63.49385	-158.39415 ± 14.37369
04H07	Glibenclamide	-65.64767 ± 10.37675	134.55830 ± 3.27213
04H08	Guanethidine sulfate	78.56379 ± 4.07623	45.66914 ± 77.84537
04H09	Quinacrine dihydrochloride hydrate	-679.30879 ± 22.86191	120.34560 ± 26.44384
04H10	Clofilium tosylate	-3.60261 ± 15.09524	6.94616 ± 19.06840
04H11	Fluphenazine dihydrochloride	76.55518 ± 2.12327	-60.70315 ± 13.62406
05A02	Streptomycin sulfate	69.15068 ± 2.50394	99.68318 ± 32.24714
05A03	Alfuzosin hydrochloride	35.77517 ± 0.35155	24.83367 ± 17.20952
05A04	Chlorpropamide	62.06152 ± 7.84492	-336.52523 ± 20.96057
05A05	Phenylpropanolamine hydrochloride	63.36314 ± 18.13605	-300.75224 ± 21.90984
05A06	Ascorbic acid	18.43220 ± 8.28496	-196.40375 ± 41.89427
05A07	Methyldopa (L,-)	61.90216 ± 17.96519	-164.41018 ± 4.23831
05A08	Cefoperazone dihydrate	53.52484 ± 16.25039	-58.38797 ± 55.95469
05A09	Zoxazolamine	52.72405 ± 12.81264	-99.88397 ± 20.00357
05A10	Tacrine hydrochloride	64.49516 ± 9.50536	-138.64683 ± 22.96079
05A11	Bisoprolol fumarate	70.54643 ± 6.10747	-131.99048 ± 26.61878
05B02	Lopinavir	56.05547 ± 14.83248	24.80134 ± 21.60533
05B03	Practolol	67.64479 ± 12.19040	23.45116 ± 4.92669
05B04	Zidovudine, AZT	35.83121 ± 18.98747	-131.60654 ± 31.34198
05B05	Sulfisoxazole	7.01000 ± 48.40385	-177.62270 ± 38.23759
05B06	Zaprinast	20.09209 ± 13.03088	-176.99256 ± 18.20130
05B07	Chlormezanone	64.15326 ± 10.18119	-171.49636 ± 11.08626
05B08	Procainamide hydrochloride	30.80648 ± 6.54926	-19.92982 ± 5.17562
05B09	N6-methyladenosine	24.23114 ± 20.61991	-47.67739 ± 7.73905
05B10	Guanfacine hydrochloride	77.62025 ± 5.26523	-47.79781 ± 5.25706
05B11	Domperidone	69.68632 ± 1.47159	-72.44234 ± 34.40086
05C02	Furosemide	47.37747 ± 5.15548	19.67232 ± 3.98228
05C03	Methapyrilene hydrochloride	58.32077 ± 6.54661	12.08093 ± 19.22172
05C04	Desipramine hydrochloride	41.30801 ± 8.40457	-105.33896 ± 5.81319
05C05	Clorgyline hydrochloride	44.23808 ± 13.62544	-153.37998 ± 60.60975
05C06	Clenbuterol hydrochloride	89.99964 ± 14.91794	-124.62437 ± 23.07518
05C07	Maprotiline hydrochloride	37.83302 ± 5.79994	-146.80941 ± 4.09329
05C08	Thioguanosine	-188.56444 ± 37.84641	-156.03185 ± 7.54936
05C09	Chlorprothixene hydrochloride	27.03608 ± 2.60120	-33.77069 ± 3.54108
05C10	Ritodrine hydrochloride	54.33598 ± 14.20596	-4.72500 ± 10.03639
05C11	Clozapine	61.03762 ± 27.03764	-53.69639 ± 21.09733
05D02	Chlorthalidone	59.33841 ± 3.76112	-16.68940 ± 7.86236
05D03	Dobutamine hydrochloride	67.89979 ± 11.44194	-54.00233 ± 4.82631

05D04	Moclobemide	11.95855 ± 23.51997	-665.03359 ± 215.72084
05D05	Clopamide	-5.74048 ± 19.77707	-392.49751 ± 182.27165
05D06	Hycanthone	-184.55352 ± 30.11525	-529.09396 ± 210.34575
05D07	Adenosine 5'-monophosphate monohydrate	54.03108 ± 10.06758	-103.43272 ± 13.74350
05D08	Amoxicillin	50.43401 ± 11.01552	15.42494 ± 3.21286
05D09	Pemirolast potassium	-6.84185 ± 3.24130	17.47710 ± 7.44370
05D10	Dextromethorphan hydrobromide monohydrate	82.06929 ± 4.79177	-5.09836 ± 4.25435
05D11	Droperidol	57.60373 ± 16.63032	-6.18932 ± 16.52931
05E02	Bambuterol hydrochloride	51.50635 ± 13.88784	-3.34226 ± 14.13979
05E03	Betamethasone	64.47785 ± 11.83118	-39.52812 ± 6.31043
05E04	Colchicine	-339.14952 ± 157.25266	-260.60169 ± 31.19611
05E05	Metergoline	-9.18668 ± 11.27382	-230.64280 ± 49.80213
05E06	Brinzolamide	62.55490 ± 8.19344	-275.33299 ± 133.79551
05E07	Ambroxol hydrochloride	62.17915 ± 7.26147	-92.63576 ± 9.18408
05E08	Benfluorex	46.86817 ± 0.65622	34.24800 ± 4.48913
05E09	Bepridil hydrochloride	78.01569 ± 8.98507	22.88956 ± 1.59283
05E10	Meloxicam	71.11711 ± 1.57643	13.87941 ± 4.24733
05E11	Benzbromarone	55.98624 ± 11.86004	-17.46532 ± 21.00051
05F02	Ketotifen fumarate	60.13253 ± 29.74998	-3.23008 ± 2.93941
05F03	Debrisoquin sulfate	42.53665 ± 15.36852	-22.38917 ± 14.98417
05F04	Amethopterin (R,S)	-351.61567 ± 159.13783	-549.34002 ± 198.28003
05F05	Methylergometrine maleate	36.05103 ± 17.58295	-280.07673 ± 194.90066
05F06	Methiothepin maleate	27.53429 ± 8.38993	-125.23227 ± 64.36974
05F07	Clofazimine	120.61670 ± 6.55688	-45.37736 ± 14.08253
05F08	Nafronyl oxalate	57.04014 ± 2.94347	34.54598 ± 15.97306
05F09	Bezafibrate	56.26015 ± 5.35208	26.30680 ± 2.98240
05F10	Nefazodone hydrochloride	81.48228 ± 4.57091	22.98158 ± 5.96650
05F11	Clebopride maleate	45.64590 ± 2.31764	21.24139 ± 5.55062
05G02	Lidoflazine	49.34493 ± 25.62277	-4.59795 ± 39.31992
05G03	Betaxolol hydrochloride	45.07729 ± 14.31081	-31.15340 ± 7.09235
05G04	Nicardipine hydrochloride	67.67366 ± 8.99267	-38.53346 ± 174.02650
05G05	Probucol	14.98182 ± 44.62480	-406.66052 ± 268.65579
05G06	Mitoxantrone dihydrochloride	-6.48045 ± 11.52454	46.02188 ± 8.91595
05G07	GBR 12909 dihydrochloride	35.00942 ± 7.12036	-22.73972 ± 14.82744
05G08	Carbetapentane citrate	55.56187 ± 10.03423	65.09041 ± 9.26409
05G09	Dequalinium dichloride	-95.16952 ± 15.42236	-28.10987 ± 9.38646
05G10	Ketoconazole	95.10669 ± 4.27094	52.26046 ± 4.28670
05G11	Fusidic acid sodium salt	53.23249 ± 0.42574	-1.23407 ± 22.43137
05H02	Terbutaline hemisulfate	23.95429 ± 10.32141	-32.54003 ± 25.79593
05H03	Ketanserin tartrate hydrate	34.25416 ± 27.44616	-39.17991 ± 10.00382
05H04	Hemicholinium bromide	24.08113 ± 4.53379	20.98232 ± 28.70128
05H05	Kanamycin A sulfate	42.81379 ± 30.96484	-241.10665 ± 186.35636
05H06	Amikacin hydrate	53.93326 ± 17.43293	-13.04593 ± 32.58389
05H07	Etoposide	-205.25629 ± 18.73735	-246.81234 ± 42.27151
05H08	Clomiphene citrate (Z,E)	22.13035 ± 5.22852	67.37206 ± 4.09284
05H09	Oxantel pamoate	37.77650 ± 19.65996	17.15848 ± 15.30472
05H10	Prochlorperazine dimaleate	85.50680 ± 1.60007	45.53929 ± 4.80912
05H11	Hesperidin	52.52767 ± 6.60850	21.75132 ± 26.98103
06A02	Testosterone propionate	79.45482 ± 12.08447	-227.96577 ± 13.60217
06A03	Haloprogin	-1.81459 ± 17.59403	-247.05292 ± 126.91576
06A04	Thyroxine (L)	60.06966 ± 13.42118	-107.92290 ± 35.63826

06A05	Idebenone	92.63597 ± 34.95044	-316.98486 ± 8.80577
06A06	Pepstatin A	68.37407 ± 3.35363	-301.04443 ± 62.25467
06A07	Delavirdine	60.38889 ± 27.66599	-401.25891 ± 64.44179
06A08	Adamantamine fumarate	99.28926 ± 20.31563	-45.94665 ± 26.01588
06A09	Butoconazole nitrate	122.20273 ± 14.31367	-114.64547 ± 59.99083
06A10	Amiodarone hydrochloride	86.62102 ± 22.67183	-82.17611 ± 57.99176
06A11	Amphotericin B	24.09889 ± 27.49612	-146.15315 ± 78.19596
06B02	Androsterone	69.44775 ± 29.33134	-139.09979 ± 13.14028
06B03	Amifostine	56.03420 ± 18.33048	-76.37365 ± 1.97337
06B04	Carbarsone	50.57013 ± 12.98368	-83.39430 ± 51.18898
06B05	Amlodipine	37.69740 ± 26.83158	-56.51495 ± 62.01041
06B06	Modafinil	100.43134 ± 65.36439	-98.86305 ± 15.96144
06B07	Bacampicillin hydrochloride	108.23560 ± 60.52719	-138.26047 ± 17.52233
06B08	Lamivudine	118.98660 ± 22.23499	-4.15257 ± 5.79597
06B09	Biotin	126.97976 ± 10.37768	-37.06065 ± 24.59464
06B10	Bisacodyl	59.21800 ± 4.64890	-44.86587 ± 18.00965
06B11	Erlotinib	-9.30597 ± 17.03709	-78.84431 ± 22.48191
06C02	Suloctidil	52.89731 ± 4.35444	-98.55474 ± 38.94109
06C03	Zotepine	-133.89643 ± 60.32674	-55.73915 ± 34.27477
06C04	Carisoprodol	53.97424 ± 8.16723	-51.32315 ± 21.12379
06C05	Cephalosporanic acid, 7-amino	48.43635 ± 2.77560	-50.82725 ± 47.11022
06C06	Chicago sky blue 6B	136.16223 ± 47.20321	87.12984 ± 15.91274
06C07	Buflomedil hydrochloride	100.20415 ± 39.10735	-89.14805 ± 10.70493
06C08	Dibenzepine hydrochloride	13.72473 ± 28.16238	-9.96946 ± 5.70572
06C09	Roxatidine Acetate hydrochloride	132.51405 ± 25.50541	-0.31983 ± 14.83324
06C10	Valacyclovir hydrochloride	-5.00780 ± 32.59282	-2.63064 ± 18.78287
06C11	Cisapride	20.80891 ± 59.19007	-25.34971 ± 7.52345
06D02	Pefloxacine	55.01215 ± 18.59580	-38.16252 ± 25.34137
06D03	Corticosterone	50.50005 ± 14.82100	-33.18660 ± 17.37058
06D04	Cyanocobalamin	56.28177 ± 10.87945	-58.21386 ± 39.56496
06D05	Cefadroxil	76.98889 ± 10.38941	14.36098 ± 22.56433
06D06	Cyclosporin A	-59.25083 ± 26.27990	-4.11972 ± 19.84392
06D07	Digitoxigenin	-542.66560 ± 334.69322	-96.46128 ± 40.42145
06D08	Digoxin	-826.71036 ± 253.16078	-61.14530 ± 24.20142
06D09	Doxorubicin hydrochloride	-613.80118 ± 237.71097	-168.79920 ± 503.77168
06D10	Carbimazole	84.25769 ± 30.54292	10.28885 ± 10.69264
06D11	Epiandrosterone	13.63374 ± 14.82946	-10.60295 ± 16.91540
06E02	Estradiol-17 beta	32.17765 ± 35.24474	-28.79821 ± 27.99086
06E03	Clobutinol hydrochloride	15.53561 ± 37.14796	17.96171 ± 10.06912
06E04	Nebivolol hydrochloride	24.26658 ± 14.23220	-101.76838 ± 28.55038
06E05	Oxcarbazepine	21.72258 ± 24.83553	5.21206 ± 17.59514
06E06	Cyclobenzaprine hydrochloride	92.26922 ± 10.02297	11.77703 ± 17.77043
06E07	Carteolol hydrochloride	81.74425 ± 43.67777	-15.02532 ± 17.62576
06E08	Hydrocortisone base	75.53702 ± 22.18811	29.47878 ± 7.39991
06E09	Pitavastatin calcium	-61.77277 ± 164.65314	-28.59899 ± 21.73812
06E10	Pilocarpine nitrate	48.89903 ± 25.56653	43.43667 ± 19.26109
06E11	Dicloxacillin sodium salt hydrate	25.33686 ± 7.34986	-35.85849 ± 14.95094
06F02	Alizapride hydrochloride	17.02231 ± 13.19211	6.41855 ± 24.88974
06F03	Stanozolol	-185.53968 ± 237.49699	25.49600 ± 5.60150
06F04	Calcipotriene	-17.42976 ± 7.54391	-142.83319 ± 30.98884
06F05	Linezolid	43.98918 ± 16.64336	64.10464 ± 20.10086

06F06	Mebhydroline 1,5-naphtalenedisulfonate	85.51532 ± 10.27295	-21.98509 ± 13.56795
06F07	Meclocycline sulfosalicylate	-153.18031± 91.36949	-1.60590 ± 11.81733
06F08	Meclozine dihydrochloride	-26.19486± 63.03996	31.94562 ± 16.66663
06F09	Melatonin	65.34385 ± 5.38380	55.18061 ± 5.78828
06F10	Butalbital	19.71288 ± 19.59498	47.14887 ± 0.97675
06F11	Dinoprost trometamol	9.91635 ± 23.64574	-24.33048 ± 21.94466
06G02	Tropisetron hydrochloride	32.13050 ± 8.68132	-35.04440 ± 33.04767
06G03	Cefixime	-4.78029 ± 8.92511	19.94611 ± 9.50050
06G04	Metrizamide	6.28184 ± 14.64040	-12.85638 ± 9.39279
06G05	Quetiapine hemifumarate	128.90584 ± 28.90584	29.89125 ± 20.99353
06G06	Tosufloxacin hydrochloride	76.45888 ± 26.53689	-8.41568 ± 62.60402
06G07	Efavirenz	57.48500 ± 37.89028	24.18781 ± 9.19627
06G08	Rifapentine	64.16809 ± 11.75139	33.81061 ± 17.70610
06G09	Neostigmine bromide	23.54112 ± 26.53689	23.96795 ± 19.78168
06G10	Niridazole	-261.29601 ± 56.76891	59.09208 ± 5.07542
06G11	Ceforanide	15.56476 ± 2.40666	-7.73667 ± 13.53703
06H02	Vatalanib	56.73574 ± 21.64743	-57.81486 ± 30.74964
06H03	Itopride	5.24616 ± 17.73347	48.67457 ± 7.83281
06H04	Cefotetan	55.01930 ± 20.80773	-23.60365 ± 13.05189
06H05	Fentiazac	113.47343 ± 13.50760	36.02693 ± 11.37816
06H06	Brompheniramine maleate	45.69307 ± 25.36893	-22.05639 ± 54.37048
06H07	Primaquine diphosphate	73.13818 ± 35.51376	28.52409 ± 11.36296
06H08	Progesterone	64.67088 ± 23.18681	65.15301 ± 11.79443
06H09	Felodipine	8.39724 ± 12.14734	51.39144 ± 14.94256
06H10	Raclopride	28.14343 ± 26.48540	47.25844 ± 12.49806
06H11	Closantel	13.69382 ± 17.08387	-44.78586 ± 26.62818
07A02	Serotonin hydrochloride	34.37955 ± 14.83348	-6.93285 ± 28.19664
07A03	Cefotiam hydrochloride	50.89141 ± 12.08648	-10.81189 ± 13.30659
07A04	Rofecoxib	29.68146 ± 39.15150	-26.50424 ± 4.60940
07A05	Benperidol	18.04722 ± 18.95718	15.71098 ± 23.96219
07A06	Cefaclor hydrate	35.56720 ± 32.48483	-0.19667 ± 5.60289
07A07	Colistin sulfate	83.76286 ± 4.49896	-1.33675 ± 13.05158
07A08	Daunorubicin hydrochloride	119.74626 ± 25.25126	-109.46572 ± 174.80628
07A09	Dosulepin hydrochloride	50.56952 ± 18.14323	31.60926 ± 18.61762
07A10	Ceftazidime pentahydrate	33.87178 ± 12.13299	39.78602 ± 47.83043
07A11	Iobenguane sulfate	-1.72524 ± 19.98528	38.62719 ± 29.76227
07B02	Metixene hydrochloride	32.02371 ± 23.75365	-31.26466 ± 19.44671
07B03	Nitrofural	29.43597 ± 39.02492	-1.99856 ± 3.74366
07B04	Omeprazole	34.18612 ± 15.11361	-16.52421 ± 5.15030
07B05	Propylthiouracil	-31.18135 ± 17.67191	-33.10990 ± 13.29056
07B06	Terconazole	28.14521 ± 23.05691	-14.84415 ± 20.80859
07B07	Tiaprofenic acid	69.46492 ± 13.38699	-14.39302 ± 20.16448
07B08	Vancomycin hydrochloride	32.45839 ± 16.48421	-20.43528 ± 2.82510
07B09	Artemisinin	-237.03055 ± 6.26330	24.32058 ± 5.65620
07B10	Propafenone hydrochloride	41.30227 ± 11.82888	38.52671 ± 7.66714
07B11	Ethamivan	34.47388 ± 19.38952	13.79066 ± 23.10330
07C02	Vigabatrin hydrochloride	23.75732 ± 17.00884	-82.56618 ± 34.70997
07C03	Biperiden hydrochloride	86.69245 ± 43.77557	-3.59916 ± 6.61845
07C04	Cetirizine dihydrochloride	-23.91507 ± 37.49739	-9.33379 ± 14.38461
07C05	Etifenin	-45.76698 ± 59.17318	-89.27584 ± 59.66944
07C06	Metaproterenol sulfate, orciprenaline sulfate	-21.13471 ± 13.08891	-33.43546 ± 9.34782

07C07	Sisomicin sulfate	26.88892 ± 11.10916	-5.68300 ± 10.13742
07C08	Sibutramine hydrochloride	78.67015 ± 6.14705	-17.30915 ± 5.94583
07C09	Acenocoumarol	63.78814 ± 21.85863	30.20760 ± 28.03679
07C10	Bromperidol	-6.08657 ± 9.56701	64.34445 ± 33.22356
07C11	Cyclizine hydrochloride	47.20734 ± 8.34896	19.94847 ± 8.31780
07D02	Fluoxetine hydrochloride	59.78046 ± 3.31066	-70.14637 ± 59.02576
07D03	Iohexol	26.42004 ± 26.01216	31.47118 ± 6.11908
07D04	Norcyclobenzaprine	42.46328 ± 15.23625	30.77026 ± 16.94533
07D05	Pyrazinamide	-5.39599 ± 31.93541	-9.09857 ± 11.82705
07D06	Trimethadione	-9.97515 ± 23.87117	-12.84972 ± 4.68169
07D07	Lovastatin	81.47136 ± 14.39356	3.06058 ± 9.92965
07D08	Nystatine	-52.37259 ± 5.33366	-23.67315 ± 8.59045
07D09	Budesonide	36.35408 ± 36.77682	10.44784 ± 19.64722
07D10	Imipenem	14.67333 ± 11.16769	-7.14563 ± 38.62664
07D11	Sulfasalazine	9.52135 ± 20.78573	15.15137 ± 13.40959
07E02	Lofexidine	63.64592 ± 36.77682	-14.90824 ± 34.39976
07E03	Thiostrepton	85.32667 ± 11.16769	-30.23590 ± 8.70070
07E04	Miglitol	20.87482 ± 19.80492	11.83294 ± 8.03822
07E05	Tiabendazole	25.18202 ± 8.12938	-18.66220 ± 22.21324
07E06	Rifampicin	-35.39176 ± 20.34829	11.15278 ± 2.84642
07E07	Ethionamide	79.12518 ± 19.80492	27.06392 ± 14.94242
07E08	Tenoxicam	61.04458 ± 31.55113	21.51413 ± 45.92171
07E09	Triflusal	68.70917 ± 12.74845	-1.83962 ± 21.55615
07E10	Mesoridazine besylate	36.21236 ± 2.40213	51.08030 ± 30.22745
07E11	Trolox	42.52317 ± 1.77192	9.34700 ± 21.85717
07F02	Pirenperone	20.32373 ± 14.33950	-24.67449 ± 41.55309
07F03	Grepafloxacin	20.48009 ± 16.03034	23.15479 ± 1.73382
07F04	Phenacetin	-13.99885 ± 18.46675	46.82636 ± 3.86757
07F05	Atovaquone	-264.31045 ± 30.48562	-3.77510 ± 26.30163
07F06	Methoxamine hydrochloride	10.14280 ± 29.39164	30.61023 ± 2.14923
07F07	(S)-(-)-Atenolol	29.03291 ± 7.97564	26.87185 ± 9.07828
07F08	Piracetam	1.39942 ± 51.05618	55.87928 ± 20.99966
07F09	Phenindione	53.06124 ± 24.29269	19.43516 ± 28.53606
07F10	Thiocolchicoside	-19.66914 ± 27.14136	42.22132 ± 12.76134
07F11	Clorsulon	20.66133 ± 11.31370	-21.23975 ± 9.22821
07G02	Ciclopirox ethanolamine	-763.48078 ± 59.63914	19.22918 ± 15.68221
07G03	Probenecid	18.16045 ± 22.75443	25.98231 ± 14.30635
07G04	Betahistine mesylate	-37.07265 ± 23.68135	45.91581 ± 9.44548
07G05	Tobramycin	6.04127 ± 4.18459	44.79159 ± 4.95111
07G06	Tetramisole hydrochloride	-3.31864 ± 35.36843	0.37061 ± 22.27424
07G07	Pregnenolone	67.56353 ± 3.26546	19.94629 ± 8.80984
07G08	Molsidomine	63.68740 ± 16.99263	-16.74293 ± 4.15511
07G09	Chloroquine diphosphate	45.30852 ± 3.35318	-14.63959 ± 11.49954
07G10	Trimetazidine dihydrochloride	30.13815 ± 13.25375	46.26989 ± 19.67115
07G11	Ropivacaine hydrochloride	65.65321 ± 26.16171	-4.01209 ± 11.98035
07H02	Hexetidine	54.40461 ± 15.61564	57.21582 ± 8.13551
07H03	Selegiline hydrochloride	10.03766 ± 9.57784	14.87830 ± 43.37422
07H04	Pentamidine isethionate	81.75836 ± 12.73613	33.10268 ± 10.41658
07H05	Tolazamide	89.43511 ± 17.40221	47.49896 ± 9.89377
07H06	Nifuroxazide	-22.22382 ± 18.98858	51.75124 ± 48.40842
07H07	Mirtazapine	65.13483 ± 10.62127	47.43578 ± 7.60606

07H08	Dirithromycin	43.49370 ± 10.70364	36.08705 ± 16.63309
07H09	Gliclazide	56.47844 ± 16.77923	-24.47282 ± 7.95205
07H10	Tazarotene	47.56425 ± 10.95250	64.64197 ± 48.02084
07H11	Prenylamine lactate	46.57229 ± 8.10280	50.11417 ± 63.84002
08A02	Ziprasidone Hydrochloride	97.70013 ± 43.18310	-17.01070 ± 63.68520
08A03	Mevastatin	-28.75423 ± 51.21003	61.68448 ± 35.70444
08A04	Pyridostigmine iodide	59.55882 ± 64.52153	26.51679 ± 29.18463
08A05	Pentobarbital	-3.67363 ± 29.85112	6.65676 ± 21.53491
08A06	Atropine sulfate monohydrate	69.03503 ± 10.43100	-160.17037 ± 102.87467
08A07	Eserine hemisulfate salt	43.65222 ± 33.90959	-714.58514 ± 39.80367
08A08	Itraconazole	95.17383 ± 22.80383	-574.11327 ± 231.09291
08A09	Acarbose	5.74887 ± 33.25647	262.11419 ± 82.33877
08A10	Entacapone	5.56332 ± 13.43610	-14.62191 ± 3798668
08A11	Nicotinamide	50.18998 ± 29.01546	-78.25652 ± 176.52813
08B02	Tetracaïne hydrochloride	6.88876 ± 22.84765	-51.20124 ± 41.47190
08B03	Mometasone furoate	-74.07135 ± 57.34023	37.20823 ± 42.05471
08B04	Troglitazone	70.89973 ± 35.04942	15.99832 ± 27.04562
08B05	Dacarbazine	19.13144 ± 31.38966	3.93255 ± 41.27535
08B06	Tenatoprazole	1.11471 ± 16.79794	-28.38657 ± 40.09449
08B07	Acetopromazine maleate salt	74.22513 ± 8.01110	-310.57207 ± 93.67959
08B08	Escitalopram oxalate	38.12866 ± 17.62676	-170.42186 ± 279.45631
08B09	Ropinirole hydrochloride	49.80631 ± 7.55746	-16.44055± 39.34411
08B10	Lacidipine	-65.07446 ± 32.41441	-39.73455 ± 15.43082
08B11	Argatroban	12.91403 ± 19.85011	13.69419 ± 11.63427
08C02	Reboxetine mesylate	-52.94510 ± 20.85886	-23.33820 ± 7.56545
08C03	Camylofine chlorhydrate	-1.96933 ± 22.56553	6.86727 ± 33.84802
08C04	Papaverine hydrochloride	-125.91096 ± 34.37517	10.01145 ± 23.88390
08C05	Yohimbine hydrochloride	-68.88845 ± 5.48573	22.01834 ± 50.21714
08C06	Voriconazole	13.96601 ± 10.58860	-495.60076 ± 146.07783
08C07	Alfacalcidol	-53.43394 ± 42.65003	-112.81556 ± 15.02876
08C08	Cilostazol	-32.88090 ± 39.94299	1.73300 ± 22.34578
08C09	Galanthamine hydrobromide	46.62547 ± 5.94279	48.13970 ± 24.69541
08C10	Azelastine hydrochloride	-25.96594 ± 28.44024	-101.06636 ± 17.05734
08C11	Etretinate	-34.34910 ± 13.47434	-198.82995± 20.78277
08D02	Emedastine	20.64629 ± 21.75410	15.35626 ± 55.75485
08D03	Etofenamate	-50.20507 ± 24.10937	-12.65434 ± 36.54711
08D04	Zaleplon	-58.40433 ± 25.00808	0.50643 ± 22.34895
08D05	Diclofenac sodium	79.35371 ± 21.75410	-29.26142 ± 27.25757
08D06	Exemestane	5.56332 ± 13.43610	-203.50421± 41.23496
08D07	Fomepizole	-0.82322 ± 12.70259	53.10021 ± 6.48206
08D08	Temozolomide	57.32140 ± 8.36023	32.66393 ± 72.91867
08D09	Xylazine	41.86460 ± 14.41980	31.86579 ± 17.14107
08D10	Celiprolol hydrochloride	2.60236 ± 25.54052	-27.87129 ± 47.30848
08D11	Zopiclone	-5.91806 ± 21.10136	-59.73839 ± 88.58230
08E02	Tranilast	-32.38015 ± 23.14534	89.82478 ± 10.58776
08E03	Tizanidine hydrochloride	-37.90595 ± 6.62892	51.63625 ± 16.30632
08E04	Zafirlukast	-75.53598 ± 77.22163	54.91370 ± 38.77010
08E05	Butenafine Hydrochloride	-5.37523 ± 40.76804	-195.10005± 84.99414
08E06	Carbadox	43.75513 ± 17.31119	38.90960 ± 13.74598
08E07	Rimantadine Hydrochloride	24.47476 ± 0.99040	49.33222 ± 17.42522
08E08	Tirofiban hydrochloride	10.15336 ± 18.12456	43.68961 ± 19.20380

08E09	Oxibendazol	-123.09184 ± 64.79970	19.68213 ± 4.66134
08E10	Ipsapirone	11.97532 ± 4.28955	-47.64120 ± 71.91011
08E11	Hydroxychloroquine sulfate	24.78841 ± 30.60806	-8.62280 ± 18.13291
08F02	Loracarbef	2.05402 ± 41.84406	40.65160 ± 27.06866
08F03	Fenipentol	1.25830 ± 33.00165	50.55770 ± 34.57920
08F04	Diosmin	-4.23785 ± 38.62874	52.10645 ± 20.75937
08F05	Carbidopa	6.11678 ± 35.20480	-24.82481 ± 86.03877
08F06	(-)-Emtricitabine	12.05397 ± 9.16995	52.25395 ± 22.50833
08F07	Demecarium bromide	-30.28116 ± 49.23762	65.05706 ± 3.30624
08F08	Tolvaptan	-24.07701 ± 7.63590	57.72757 ± 3.07690
08F09	Acipimox	-34.53870 ± 86.68573	12.19347 ± 1.04942
08F10	Diflorasone Diacetate	8.06132 ± 19.61320	21.50613 ± 51.98236
08F11	Acamprosate calcium	-8.17702 ± 25.42611	22.11268 ± 1.25362
08G02	Mizolastine	15.83064 ± 12.59417	51.39990 ± 21.00403
08G03	Amisulpride	-73.29051 ± 76.83895	5.75202 ± 10.36929
08G04	Pyridoxine hydrochloride	7.64969 ± 11.12957	18.97581 ± 7.64352
08G05	Mercaptopurine	45.75651 ± 21.19518	-95.38150 ± 13.09630
08G06	Cytarabine	-106.24293 ± 25.41122	59.03054 ± 3.15023
08G07	Racecadotril	-6.21005 ± 9.86390	27.90559 ± 21.98350
08G08	Folic acid	5.90913 ± 38.58182	64.86031 ± 4.11116
08G09	Benazepril hydrochloride	-34.33781 ± 17.37992	1.89665 ± 18.61255
08G10	Aniracetam	11.01299 ± 6.56759	-74.73600 ± 57.59482
08G11	Dimethisoquin hydrochloride	18.57785 ± 29.34760	36.84854 ± 12.22729
08H02	Alendronate sodium	27.54750 ± 47.06633	65.01094 ± 23.72578
08H03	Dipivefrin hydrochloride	-7.77536 ± 24.81280	-4.44744 ± 17.77698
08H04	Thiorphan	-14.26964 ± 89.08241	36.47350 ± 23.66169
08H05	Tomoxetine hydrochloride	108.33035 ± 29.30803	-540.72158 ± 11.55522
08H06	Aceclidine Hydrochloride	55.35102 ± 10.66087	84.89165 ± 12.57747
08H07	Penciclovir	15.18875 ± 46.86660	-31.37349 ± 63.83421
08H08	Levetiracetam	-1.84278 ± 11.71876	63.86071 ± 11.49793
08H09	Dexfenfluramine hydrochloride	30.82537 ± 20.02504	23.26836 ± 4.69045
08H10	Etoricoxib	58.26036 ± 28.02786	9.22488 ± 15.25612
08H11	Sertindole	-311.31818 ± 63.33245	0.54901 ± 11.25032
09A02	Sulmazole	60.48213 ± 18.87866	-645.35124 ± 149.24134
09A03	Gefitinib	36.67769 ± 23.34478	-613.62524 ± 19.60449
09A04	Flunisolide	5.36131 ± 8.22333	-521.83576 ± 23.87340
09A05	N-Acetyl-DL-homocysteine Thiolactone	62.22496 ± 23.18858	-434.20257 ± 8.19885
09A06	Flurandrenolide	-73.45565 ± 37.81933	-119.86897 ± 26.18598
09A07	Oxiconazole Nitrate	-17.68696 ± 5.32216	-149.82412 ± 24.23100
09A08	Rebamipide	10.11692 ± 14.66534	-145.72551 ± 25.81636
09A09	Nilvadipine	49.31528 ± 15.73850	-70.31357 ± 20.69608
09A10	Etanidazole	20.31649 ± 11.42197	-124.01309 ± 20.50287
09A11	Pinaverium bromide	54.25088 ± 4.20711	-124.07078 ± 7.27799
09B02	Glimepiride	-7.41745 ± 11.77882	-193.79877 ± 73.98795
09B03	Picrotoxinin	-28.04694 ± 18.12400	-189.66722 ± 40.98991
09B04	Mepenzolate bromide	13.75636 ± 1.65959	-252.37958 ± 24.13095
09B05	Benfotiamine	15.49049 ± 4.22626	-287.17777 ± 14.60126
09B06	Halcinonide	11.04927 ± 21.35020	-26.25251 ± 4.58163
09B07	Lanatoside C	-39.90405 ± 15.44171	-253.63386 ± 44.90785
09B08	Benzamil hydrochloride	6.46292 ± 8.52268	-87.68858 ± 13.53009
09B09	Suxibuzone	41.71960 ± 0.67797	-76.30925 ± 8.58517

09B10	6-Furfurylaminopurine	38.30652 ± 13.56749	-26.55413 ± 6.58479
09B11	Avermectin B1	-39.40487 ± 17.79274	-32.56248 ± 5.12697
09C02	Pranlukast	-10.09108 ± 8.85765	-75.80569 ± 121.91347
09C03	D,L-Penicillamine	35.96370 ± 9.80584	-155.45355 ± 13.50777
09C04	Zileuton	6.40888 ± 23.30659	-116.15148 ± 28.06462
09C05	Loratadine	-13.61494 ± 21.20809	-155.48822 ± 8.94351
09C06	Tetraethylenepentamine pentahydrochloride	-25.59912 ± 20.16044	-58.59398 ± 8.55675
09C07	Nisoldipine	27.25193 ± 2.19572	-244.68969 ± 29.00602
09C08	Acefylline	27.51168 ± 4.62888	-51.28332 ± 6.11098
09C09	Acitretin	15.50077 ± 2.94608	-35.73468 ± 22.51285
09C10	Zonisamide	35.50492 ± 5.77825	-13.60289 ± 3.74950
09C11	Irsogladine maleate	36.49825 ± 9.36768	-17.70678 ± 1.40270
09D02	Dydrogesterone	52.16047 ± 22.09415	-100.78844 ± 217.11446
09D03	Sumatriptan succinate	12.57199 ± 7.37840	26.42850 ± 30.96000
09D04	Opipramol dihydrochloride	24.72757 ± 17.86938	-74.71932 ± 23.36314
09D05	Nalidixic acid sodium salt	-5.27019 ± 13.50949	-99.31401 ± 3.86476
09D06	Oxacillin sodium	-5.34890 ± 18.93301	1.04657 ± 10.54479
09D07	Beta-Escin	-1.96151 ± 14.63139	-73.35306 ± 11.11296
09D08	Thiamine hydrochloride	9.82950 ± 8.05082	-37.04463 ± 12.04800
09D09	Tazobactam	22.84360 ± 7.11888	-20.13516 ± 26.30771
09D10	Ibandronate sodium	32.84230 ± 5.95568	27.40532 ± 6.18348
09D11	Warfarin	25.83063 ± 2.63791	11.29505 ± 4.20323
09E02	Pranoprofen	20.31212 ± 24.65981	-534.99433 ± 55.32492
09E03	Secnidazole	-6.37644 ± 9.11071	14.82526 ± 12.19246
09E04	Pempidine	21.37298 ± 5.30646	-0.50472 ± 11.62500
09E05	Mirabegron	4.10291 ± 2.35267	-4.75009 ± 6.81666
09E06	Ibutilide fumarate	37.73275 ± 19.17071	-8.51645 ± 7.63969
09E07	Tigecycline	25.56401 ± 4.11619	6.91117 ± 6.15946
09E08	Tramadol hydrochloride	25.41789 ± 9.88595	-9.62910 ± 14.23930
09E09	Estropipate	5.81859 ± 13.48549	-67.45578 ± 11.19492
09E10	Butylscopolammonium (n-) bromide	24.49639 ± 20.89473	9.94858 ± 5.92558
09E11	Irinotecan hydrochloride trihydrate	-103.81070 ± 12.58744	-118.70793 ± 3.63716
09F02	Tylosin	17.94067 ± 26.31815	-335.90051 ± 13.08403
09F03	Citalopram Hydrobromide	75.50361 ± 20.89473	80.56429 ± 8.38757
09F04	Promazine hydrochloride	59.07791 ± 10.81015	-106.66710 ± 11.77408
09F05	Sulfamerazine	14.60759 ± 17.76922	33.71321 ± 2.90903
09F06	Venlafaxine	-7.71117 ± 20.69573	36.41932 ± 5.83065
09F07	Ethotoin	-14.00658 ± 12.07587	25.10860 ± 0.75894
09F08	3-alpha-Hydroxy-5-beta-androstan-17-one	7.32883 ± 13.69139	20.72663 ± 0.39712
09F09	Tetrahydrozoline hydrochloride	-17.55658 ± 6.91739	-51.83566 ± 10.87646
09F10	Hexestrol	29.81206 ± 0.85001	46.43533 ± 8.71928
09F11	Cefmetazole sodium salt	17.40109 ± 19.13939	35.08136 ± 4.31538
09G02	Trihexyphenidyl-D,L Hydrochloride	2.35667 ± 33.75470	-97.38331 ± 16.75174
09G03	Succinylsulfathiazole	8.75502 ± 6.86157	10.77003 ± 0.55655
09G04	Famprofazone	44.56442 ± 6.07850	-521.08163 ± 125.07944
09G05	Bromopride	4.13865 ± 10.60733	9.09603 ± 0.49669
09G06	Methyl benzethonium chloride	-154.40555 ± 16.79714	14.04918 ± 7.84401
09G07	Chlorcyclizine hydrochloride	32.26613 ± 3.65718	-6.41950 ± 27.26088
09G08	Diphenylpyraline hydrochloride	16.32414 ± 9.00730	33.67945 ± 10.20304
09G09	Benzethonium chloride	51.11192 ± 19.68334	-202.88795 ± 32.13381
09G10	Trioxsalen	28.12989 ± 5.47121	54.53210 ± 3.91625

09G11	Doxofylline	36.15446 ± 12.79354	35.09072 ± 16.56132
09H02	Sulfabenzamide	60.48213 ± 18.87866	-137.50808 ± 29.60984
09H03	Benzocaine	-25.36728 ± 26.65742	40.28527 ± 24.91206
09H04	Dipyrone	107.53995 ± 10.69835	108.97180 ± 21.44710
09H05	Isosorbide dinitrate	110.98347 ± 10.80006	109.29434 ± 8.30885
09H06	Sulfachloropyridazine	39.53029 ± 11.25773	42.31840 ± 17.59608
09H07	Pramoxine hydrochloride	34.12742 ± 8.81206	14.70204 ± 25.87531
09H08	Finasteride	35.79007 ± 3.75852	56.47268 ± 13.24498
09H09	Fluorometholone	34.46735 ± 2.55413	-10.27271 ± 1.31186
09H10	Cephalothin sodium salt	57.31314 ± 5.73470	31.31849 ± 23.02693
09H11	Cefuroxime sodium salt	47.10031 ± 7.87565	50.49302 ± 20.43201
10A02	Althiazide	37.78175 ± 1.84248	-85.79224 ± 12.07320
10A03	Isopyrin hydrochloride	41.97241 ± 5.68327	-169.96329 ± 36.68147
10A04	Phenethicillin potassium salt	53.33743 ± 9.83327	-149.74070 ± 8.49465
10A05	Sulfamethoxypyridazine	24.08153 ± 6.49966	-190.71600 ± 13.30384
10A06	Deferoxamine mesylate	15.55711 ± 16.32340	-233.28088 ± 7.81617
10A07	Mephentermine hemisulfate	15.61658 ± 6.28492	-613.62524 ± 19.60449
10A08	Liranaftate	40.20552 ± 16.90159	-521.83576 ± 23.87340
10A09	Sulfadimethoxine	1.97988 ± 7.04239	-434.20257 ± 8.19885
10A10	Sulfanilamide	-5.69867 ± 8.57155	-365.40300 ± 4.49638
10A11	Balsalazide Sodium	-20.04954 ± 19.04387	-376.54661 ± 63.28873
10B02	Sulfaquinoxaline sodium salt	49.70918 ± 8.82353	-47.49626 ± 10.02650
10B03	Streptozotocin	27.26713 ± 14.89388	-101.47672 ± 22.30995
10B04	Metoprolol-(+,-) (+)-tartrate salt	-17.40772 ± 3.58718	-70.04093 ± 18.64012
10B05	Flumethasone	-11.26315 ± 6.88968	-163.32371 ± 14.50516
10B06	Flecainide acetate	9.35777 ± 6.17694	-148.80038 ± 13.28586
10B07	Cefazolin sodium salt	1.26338 ± 4.49155	-189.66722 ± 40.98991
10B08	Trimetozine	1.22745 ± 3.13631	-252.37958 ± 24.13095
10B09	Folinic acid calcium salt	-5.65539 ± 7.28699	-287.17777 ± 14.60126
10B10	Levonordefrin	-22.28838 ± 20.05074	-99.65900 ± 9.45039
10B11	Amprenavir	33.30460 ± 3.79879	-158.92238 ± 33.66067
10C02	Bimatoprost	37.89790 ± 10.66702	-25.59258 ± 6.46656
10C03	Sulfamethizole	36.15899 ± 3.28403	-75.83522 ± 20.60448
10C04	Medrysone	2.88519 ± 7.53856	-72.30479 ± 6.67119
10C05	Flunixin meglumine	-2.35592 ± 2.31768	-104.26912 ± 11.09230
10C06	Spiramycin	5.25989 ± 8.03242	-98.61919 ± 10.05412
10C07	Glycopyrrolate	18.70768 ± 7.86542	-155.45355 ± 13.50777
10C08	Aprepitant	26.03082 ± 12.79035	-116.15148 ± 28.06462
10C09	Monensin sodium salt	-464.89071 ± 72.54135	-155.48822 ± 8.94351
10C10	Isoetharine mesylate salt	-46.98830 ± 10.74467	-100.72268 ± 43.36689
10C11	Dronedarone hydrochloride	-198.83447 ± 6.08377	-84.65671 ± 52.17430
10D02	Terazosin hydrochloride	44.13121 ± 6.09977	12.11106 ± 3.85751
10D03	Phenazopyridine hydrochloride	-87.80585 ± 48.75067	-26.35318 ± 8.11660
10D04	Demeclocycline hydrochloride	-414.84329 ± 108.02965	-4.02482 ± 4.11485
10D05	Fenoprofen calcium salt dihydrate	-13.91873 ± 3.17172	-69.14513 ± 2.37455
10D06	Piperacillin sodium salt	-14.62366 ± 4.41223	-103.61377 ± 16.33977
10D07	Diethylstilbestrol	-10.31472± 4.48612	26.42850 ± 30.96000
10D08	Chlorotrianisene	27.91802 ± 14.62303	-74.71932 ± 23.36314
10D09	Ribostamycin sulfate salt	-38.95549 ± 8.64296	-99.31401 ± 3.86476
10D10	Methacholine chloride	-11.85355 ± 5.15539	46.02878 ± 10.86865
10D11	Pipenzolate bromide	21.50455 ± 7.33752	8.58157 ± 6.77789

10E02	Butamben	1.38484 ± 6.12741	16.66923 ± 1.08409
10E03	Sulfapyridine	32.11769 ± 9.96181	7.05133 ± 0.73424
10E04	Meclofenoxate hydrochloride	-12.71055 ± 5.69219	-12.81422 ± 8.99577
10E05	Furaltadone hydrochloride	-42.92366 ± 22.27244	-33.75114 ± 11.14756
10E06	Ethoxyquin	-116.37228 ± 11.37803	-534.99433 ± 55.32492
10E07	Tinidazole	-28.89032 ± 9.65045	14.82526 ± 12.19246
10E08	Guanadrel sulfate	3.55039 ± 11.11277	-0.50472 ± 11.62500
10E09	Vidarabine	-40.62650 ± 12.44848	-4.75009 ± 6.81666
10E10	Sulfameter	-13.60893 ± 14.62970	47.82687 ± 11.71954
10E11	Isopropamide iodide	9.62376 ± 4.60829	43.59367 ± 29.16003
10F02	Alclometasone dipropionate	53.88290 ± 8.42704	31.55497 ± 4.89234
10F03	Leflunomide	-7.07321 ± 12.11064	22.75684 ± 15.47013
10F04	Norgestrel-(-)-D	-5.10487 ± 13.73525	28.43933 ± 10.90468
10F05	Fluocinonide	-6.16615 ± 13.68432	-11.70097 ± 19.37287
10F06	Sulfamethazine sodium salt	7.64603 ± 8.40066	-335.90051 ± 13.08403
10F07	Guaifenesin	0.13858 ± 12.99289	27.21617 ± 18.32499
10F08	Alexidine dihydrochloride	-616.47660 ± 101.15332	-106.66710 ± 11.77408
10F09	Proadifen hydrochloride	-48.19199 ± 20.39997	33.71321 ± 2.90903
10F10	Zomepirac sodium salt	-52.14507 ± 9.49626	33.48421 ± 23.40852
10F11	Cinoxacin	9.33281 ± 8.36950	52.03415 ± 20.60604
10G02	Clobetasol propionate	56.18211 ± 23.16444	60.50471 ± 6.18091
10G03	Podophyllotoxin	-512.21540 ± 74.07904	-184.46371 ± 38.21992
10G04	Clofibric acid	6.21902 ± 12.09724	26.67300 ± 18.81925
10G05	Bendroflumethiazide	-14.14516 ± 6.03071	35.89078 ± 10.58057
10G06	Dicumarol	15.78816 ± 8.42024	-97.38331 ± 16.75174
10G07	Methimazole	-2.40498 ± 15.48486	10.77003 ± 0.55655
10G08	Merbromin	84.67985 ± 24.69662	-521.08163 ± 125.07944
10G09	Hexylcaine hydrochloride	-32.89379 ± 4.91764	66.26089 ± 29.11804
10G10	Drofenine hydrochloride	-59.32893 ± 77.93402	34.63923 ± 23.74815
10G11	Cycloheximide	-209.80021 ± 15.28103	25.82598 ± 40.98295
10H02	(R) -Naproxen sodium salt	63.39959 ± 3.50613	72.83009 ± 2.58478
10H03	Propidium iodide	34.04835 ± 9.06904	31.28988 ± 22.09339
10H04	Cloperastine hydrochloride	44.16707 ± 6.26417	26.99032 ± 41.34401
10H05	Eprosartan mesylate	30.28407 ± 3.21183	42.02713 ± 10.14379
10H06	Isocarboxazid	27.15171 ± 4.97613	-137.50808 ± 29.60984
10H07	Lithocholic acid	-11.27211 ± 9.74533	10.32485 ± 0.94042
10H08	Methotrimeprazine maleat salt	41.49645 ± 5.83961	-13.44878 ± 26.47594
10H09	Dienestrol	18.42572 ± 5.52124	10.92943 ± 0.83088
10H10	Pridinol methanesulfonate salt	6.84607 ± 3.37068	12.81021 ± 0.51032
10H11	Amrinone	24.53002 ± 3.54368	11.77540 ± 2.04697
11A02	Carbinoxamine maleate salt	-21.46093 ± 14.35927	-281.60189 ± 32.82583
11A03	Methazolamide	42.27194 ± 16.81202	-293.46755 ± 43.50813
11A04	Pyrithyldione	51.65135 ± 13.35628	-148.95816 ± 20.03009
11A05	Spectinomycin dihydrochloride	9.87080 ± 19.74523	-127.24175 ± 11.12002
11A06	Piromidic acid	6.28384 ± 3.39551	-100.70762 ± 0.90315
11A07	Trimipramine maleate salt	-14.32629 ± 11.61543	-315.90877 ± 68.54106
11A08	Chloropyramine hydrochloride	4.34519 ± 11.33150	-547.27747 ± 22.23658
11A09	Furazolidone	-294.78861 ± 118.89264	-371.22503 ± 42.12617
11A10	Dichlorphenamide	-1.72010 ± 11.70424	-274.33601 ± 35.63436
11A11	Sulconazole nitrate	14.45584 ± 11.70344	-159.90286 ± 11.82119
11B02	Auranofin	-260.38615 ± 42.96579	-423.02950 ± 174.30590

11B03	Cromolyn disodium salt	14.89487 ± 2.76173	-284.87013 ± 26.39593
11B04	Bucladesine sodium salt	31.46029 ± 7.45479	-129.51894 ± 33.21087
11B05	Cefsulodin sodium salt	-6.19521 ± 7.21313	-351.27696 ± 33.92652
11B06	Fosfosal	-14.03036 ± 4.00119	-433.99114 ± 66.27110
11B07	Suprofen	-8.58602 ± 10.52377	-12.84062 ± 27.21230
11B08	Deflazacort	-0.94838 ± 6.45136	-189.76231 ± 53.78819
11B09	Nadolol	18.24942 ± 6.63969	-175.71562 ± 45.49225
11B10	Moxalactam disodium salt	12.81972 ± 3.87555	-257.40285 ± 51.05659
11B11	Aminophylline	16.00284 ± 18.42954	-183.90287 ± 60.37627
11C02	Azlocillin sodium salt	2.96654 ± 10.89128	-117.26171 ± 34.69157
11C03	Clidinium bromide	12.36201 ± 3.22766	-84.33191 ± 17.79081
11C04	Sulfamonomethoxine	-22.25837 ± 43.92520	-287.34055 ± 141.34210
11C05	Benzthiazide	-14.55233 ± 3.63677	-237.30223 ± 53.22886
11C06	Trichlormethiazide	-17.96538 ± 9.55767	-202.58710 ± 47.30253
11C07	Oxalamine citrate salt	-505.21482 ± 119.78034	-69.84647 ± 41.69426
11C08	Propantheline bromide	9.72392 ± 8.13236	-171.26359 ± 12.74346
11C09	Viloxazine hydrochloride	1.64739 ± 15.14359	-225.27722 ± 147.21253
11C10	Dimethadione	-7.49954 ± 9.64230	-141.84498 ± 74.39717
11C11	Ethaverine hydrochloride	37.86469 ± 7.33284	-420.43381 ± 57.64854
11D02	Butacaine	1.38484 ± 6.12741	-20.02292 ± 16.63478
11D03	Cefoxitin sodium salt	10.65900 ± 11.17416	-106.86551 ± 24.01261
11D04	Eszopiclone	11.97207 ± 8.89984	96.42119 ± 55.77945
11D05	Novobiocin sodium salt	8.95340 ± 18.70966	79.85695 ± 39.33453
11D06	Zolmitriptan	-4.37232 ± 8.71363	-4.45068 ± 43.32866
11D07	Indoprofen	4.30309 ± 1.36876	13.48844 ± 26.73135
11D08	Carbenoxolone disodium salt	13.87998 ± 12.24936	18.96970 ± 40.79050
11D09	Iocetamic acid	27.42928 ± 8.73465	-9.63038 ± 8.23023
11D10	Ganciclovir	7.93347 ± 12.18784	-93.02283 ± 36.76890
11D11	Ethopropazine hydrochloride	58.86651 ± 7.55037	33.57246 ± 0.54511
11E02	Olanzapine	21.83586 ± 21.65546	19.90215 ± 19.80186
11E03	Trimeprazine tartrate	37.19861 ± 4.59784	-851.54420 ± 31.36944
11E04	Nafcillin sodium salt monohydrate	4.79943 ± 12.76317	124.97517 ± 60.39513
11E05	Procyclidine hydrochloride	20.84640 ± 11.63032	65.61946 ± 34.06816
11E06	Amiprilose hydrochloride	-8.10621 ± 4.80956	-6.52117 ± 64.57493
11E07	Ethynylestradiol 3-methyl ether	-9.72949 ± 5.62339	54.80874 ± 13.56476
11E08	(-) -Levobunolol hydrochloride	-0.39851 ± 10.42446	31.37810 ± 9.07646
11E09	Iodixanol	8.79140 ± 17.97690	30.90297 ± 21.24466
11E10	Clinafloxacin	-25.08176 ± 15.27531	-129.18981 ± 30.73448
11E11	Equilin	37.68196 ± 8.65034	10.30639 ± 85.74806
11F02	Paroxetine Hydrochloride	22.18880 ± 6.41113	56.16143 ± 9.91913
11F03	Nylidrin	26.86090 ± 4.39222	-482.93932 ± 191.46242
11F04	Liothyronine	39.25525 ± 5.47692	15.12710 ± 2.03141
11F05	Roxithromycin	19.77514 ± 2.72170	14.63117 ± 3.78634
11F06	Beclomethasone dipropionate	-17.26692 ± 5.23293	3.37401 ± 6.98131
11F07	Tolmetin sodium salt dihydrate	-34.44557 ± 27.10965	7.62096 ± 0.70226
11F08	(+) -Levobunolol hydrochloride	24.42927 ± 8.89189	41.58849 ± 17.73184
11F09	Doxazosin mesylate	39.29069 ± 1.25435	6.67646 ± 0.80766
11F10	Fluvastatin sodium salt	-17.96488 ± 29.23449	-166.63820 ± 36.59030
11F11	Methylhydantoin-5-(L)	9.37478 ± 7.53107	32.47076 ± 2.79376
11G02	Gabapentin	4.40407 ± 2.16924	10.22423 ± 0.94841
11G03	Raloxifene hydrochloride	-14.19426 ± 14.66265	-115.33130 ± 69.35085

11G04	Ciclesonide	-6.32296 ± 13.44590	38.38715 ± 19.75449
11G05	Methylhydantoin-5-(D)	5.37625 ± 6.78851	6.25979 ± 23.37655
11G06	Simvastatin	-154.47332 ± 125.41829	-249.17564 ± 64.40268
11G07	Azacytidine-5	-47.55113 ± 18.78377	27.35202 ± 14.09469
11G08	Paromomycin sulfate	22.18880 ± 6.41113	6.16439 ± 19.64448
11G09	Acetaminophen	24.10433 ± 7.26988	74.14730 ± 37.61753
11G10	Phthalylsulfathiazole	18.34907 ± 6.70522	-73.34807 ± 49.89622
11G11	Luteolin	8.65311 ± 15.53764	32.79041 ± 2.06252
11H02	Iopamidol	19.76784 ± 19.81259	12.35531 ± 0.60544
11H03	Iopromide	9.38148 ± 17.40219	-117.58008 ± 94.14649
11H04	Theophylline monohydrate	11.56640 ± 7.89496	20.88534 ± 3.76173
11H05	Theobromine	80.23216 ± 19.81259	-1.45698 ± 12.86545
11H06	Reserpine	-251.39043 ± 88.20792	13.58900 ± 0.52003
11H07	Bicalutamide	21.26943 ± 9.52107	12.45160 ± 1.65692
11H08	Scopolamine hydrochloride	15.17905 ± 9.94921	20.33345 ± 88.30351
11H09	Ioversol	35.16779 ± 1.07413	58.93011 ± 24.12509
11H10	Rabeprazole Sodium salt	43.44769 ± 7.45247	-199.75289 ± 89.35603
11H11	Carbachol	38.69334 ± 4.40082	32.05886 ± 4.00251
12A02	Niacin	56.76292 ± 8.86009	-166.34268 ± 20.68262
12A03	Bemegride	-4.68161 ± 22.07079	-192.14719 ± 19.34859
12A04	Digoxigenin	-25.15958 ± 17.63685	-289.35490 ± 17.85918
12A05	Meglumine	-0.91682 ± 16.60440	-161.30086 ± 6.62304
12A06	Dolasetron mesilate	-8.94061 ± 17.45307	-127.51330 ± 15.67400
12A07	Clioquinol	-11.61770 ± 24.12419	-127.05095 ± 12.76151
12A08	Oxybenzone	51.71247 ± 14.33679	-531.62602 ± 33.59839
12A09	Promethazine hydrochloride	46.96279 ± 4.69349	-191.51373 ± 43.27311
12A10	Diacerein	9.71335 ± 9.76355	-326.81380 ± 92.48550
12A11	Esmolol hydrochloride	25.81329 ± 6.06255	30.44632 ± 17.15771
12B02	Cortisol acetate	26.32996 ± 10.68911	-112.42579 ± 30.69289
12B03	Flubendazol	-637.93194 ± 57.38947	-348.57444 ± 41.71908
12B04	Felbinac	7.38188 ± 12.71246	-48.35232 ± 15.38280
12B05	Butylparaben	32.44397 ± 12.92691	-153.15086 ± 11.29420
12B06	Aminohippuric acid	49.28899 ± 1.87999	-66.90115 ± 10.29840
12B07	N-Acetyl-L-leucine	17.81546 ± 13.12052	-74.53902 ± 18.48753
12B08	Pipemidic acid	18.92685 ± 11.75097	-59.56360 ± 32.48414
12B09	Dioxybenzone	12.57463 ± 9.30821	-187.91166 ± 64.23279
12B10	Adrenosterone	43.90318 ± 3.90568	-144.88454 ± 30.70129
12B11	Methylatropine nitrate	35.74598 ± 10.28631	18.64540 ± 18.62512
12C02	Hymecromone	36.33403 ± 18.47852	-79.19938 ± 15.28778
12C03	Abacavir Sulfate	-0.74415 ± 11.26755	-113.94817 ± 16.22433
12C04	Diloxanide furoate	32.15909 ± 15.69945	-45.16479 ± 14.23619
12C05	Metyrapone	36.14781 ± 6.17736	-32.14311 ± 12.68329
12C06	Urapidil hydrochloride	34.84554 ± 10.16838	-36.80697 ± 13.83889
12C07	Fluspirilen	-10.84165 ± 10.27041	-30.36333 ± 3.60382
12C08	S-(+)-ibuprofen	15.84426 ± 16.80575	-95.18219 ± 24.14357
12C09	Ethynodiol diacetate	13.73435 ± 15.30366	-93.20332 ± 17.47111
12C10	Nabumetone	11.68694 ± 14.14257	-95.11677 ± 22.46068
12C11	Nisoxetine hydrochloride	32.82669 ± 8.67328	10.62513 ± 16.34332
12D02	(+)-Isoproterenol (+)-bitartrate salt	38.77908 ± 2.98899	-162.89835 ± 100.42476
12D03	Monobenzone	-147.76973 ± 15.58128	-107.09013 ± 6.34390
12D04	2-Aminobenzenesulfonamide	35.79973 ± 16.69144	2.27858 ± 3.62140

12D05	Estrone	21.60714 ± 8.94321	-24.00836 ± 5.35135
12D06	Lorglumide sodium salt	53.04016 ± 5.10924	1.47944 ± 3.65729
12D07	Nitrendipine	23.49121 ± 13.04912	-98.44803 ± 55.86087
12D08	Flurbiprofen	39.97323 ± 14.36641	27.56535 ± 44.02329
12D09	Nimodipine	29.86323 ± 6.30565	33.34687 ± 27.58806
12D10	Bacitracin	18.14229 ± 12.11126	15.40619 ± 13.32258
12D11	Gemifloxacin mesylate	35.57820 ± 4.14651	35.44217 ± 18.22612
12E02	Nizatidine	12.81090 ± 17.04886	7.09575 ± 14.24767
12E03	Thioperamide maleate	20.92861 ± 5.29216	-431.56449 ± 16.75460
12E04	Xamoterol hemifumarate	44.52067 ± 5.86768	17.59123 ± 5.32827
12E05	Posaconazole	-120.97376 ± 36.60222	21.75770 ± 13.64586
12E06	Thonzonium bromide	-92.42921 ± 23.98181	-6.59954 ± 16.40144
12E07	Idazoxan hydrochloride	25.59311 ± 0.67505	-326.65464 ± 66.27954
12E08	Quinapril hydrochloride	34.16694 ± 17.91931	19.78840 ± 25.65113
12E09	Nilutamide	26.24870 ± 10.87166	41.08000 ± 23.40767
12E10	Ketorolac tromethamine	29.62729 ± 10.21421	27.84678 ± 26.81914
12E11	Protriptyline hydrochloride	22.71754 ± 5.20735	26.87403 ± 9.08920
12F02	Propofol	58.41269 ± 8.59915	36.94391 ± 10.69581
12F03	S(-)Eticlopride hydrochloride	16.19539 ± 4.64570	-238.73879 ± 18.85198
12F04	Primidone	24.92838 ± 14.41597	45.56589 ± 21.89805
12F05	Flucytosine	32.18309 ± 10.56228	40.13747 ± 15.99097
12F06	Paliperidone	18.27184 ± 8.04260	49.38278 ± 7.15525
12F07	Bephenium hydroxynaphthoate	29.71139 ± 8.93747	-207.21388 ± 68.43639
12F08	Dehydroisoandosterone 3-acetate	37.39625 ± 9.02144	10.42536 ± 1.67023
12F09	Benserazide hydrochloride	30.50650 ± 5.53934	50.48531 ± 18.64058
12F10	Iodipamide	43.35060 ± 9.65956	23.30221 ± 11.20768
12F11	Allopurinol	44.82322 ± 8.66967	-7.00157 ± 19.85751
12G02	Pentetic acid	29.43731 ± 7.49686	-18.53701 ± 48.47422
12G03	Bretylium tosylate	14.52106 ± 9.13171	10.73440 ± 12.58651
12G04	Pralidoxime chloride	23.82487 ± 17.19133	45.15000 ± 18.66233
12G05	Phenoxybenzamine hydrochloride	4.91863 ± 30.80207	47.41989 ± 28.40705
12G06	Salmeterol	33.02289 ± 17.38752	24.16999 ± 13.92867
12G07	Altretamine	8.07595 ± 9.05961	-95.11677 ± 22.46068
12G08	Prazosin hydrochloride	11.61417 ± 20.41457	12.51937 ± 1.76158
12G09	Timolol maleate salt	31.05010 ± 4.03786	-9.69539 ± 24.59016
12G10	(+,-)-Octopamine hydrochloride	48.72089 ± 7.30728	33.20470 ± 21.87328
12G11	Stavudine	42.13077 ± 10.53935	-447.52313 ± 92.45019
12H02	Crotamiton	49.61649 ± 0.12968	23.42420± 31.12958
12H03	Toremifene	0.88300 ± 11.16223	-196.59517 ± 64.24213
12H04	(R)-(+)-Atenolol	2.93220 ± 24.71426	10.30050 ± 0.10449
12H05	Tyloxapol	49.95360 ± 9.27658	41.44740 ± 27.37647
12H06	Florfenicol	8.08840 ± 4.42624	74.27396 ± 6.88830
12H07	Megestrol acetate	43.59042 ± 16.07817	-111.28234 ± 54.29128
12H08	Deoxycorticosterone	52.96903 ± 13.88321	15.10441 ± 1.03221
12H09	Urosiol	36.57243 ± 10.93429	-5.72396 ± 19.34406
12H10	Proparacaine hydrochloride	49.69897 ± 6.73389	14.83534 ± 0.92335
12H11	Aminocaproic acid	51.07876 ± 16.10377	-313.71781 ± 22.16804
13A02	Denatonium benzoate	36.18593 ± 15.94016	16.49970 ± 5.37021
13A03	Canrenone	27.14402 ± 25.08027	10.12967 ± 2.23971
13A04	Enilconazole	12.74878 ± 58.94703	12.72557 ± 0.83850
13A05	Methacycline hydrochloride	11.39699 ± 17.32418	27.16179 ± 0.73985

13A06	Floxuridine	-216.60497 ± 93.88455	30.88804 ± 0.76264
13A07	Sotalol hydrochloride	25.58960 ± 16.36081	32.55307 ± 14.06864
13A08	Gestrinone	19.51172 ± 5.92744	-344.68438 ± 148.65847
13A09	Decamethonium bromide	-7.01906 ± 11.44688	-392.17223 ± 101.31102
13A10	Darifenacin hydrobromide	64.37499 ± 11.07431	-383.94388 ± 113.91955
13A11	Indatraline hydrochloride	84.91893 ± 7.42216	-332.00726 ± 138.39178
13B02	Remoxipride Hydrochloride	11.03976 ± 24.04549	-42.85746 ± 46.33710
13B03	THIP Hydrochloride	26.10114 ± 7.00821	22.34602 ± 8.19247
13B04	Pirlindole mesylate	34.72630 ± 10.94348	-169.36748 ± 50.63290
13B05	Pronethalol hydrochloride	8.78903 ± 22.72432	35.32893 ± 14.31281
13B06	Naftopidil dihydrochloride	22.54096 ± 8.75207	41.46338 ± 2.54176
13B07	Tracazolate hydrochloride	9.44984 ± 14.70616	35.15631 ± 1.72770
13B08	Zardaverine	13.11251 ± 8.74813	-536.12492 ± 105.03049
13B09	Memantine Hydrochloride	77.45904 ± 8.75207	-440.55850 ± 88.41384
13B10	Ozagrel hydrochloride	39.76384 ± 5.58756	-448.94256 ± 55.76781
13B11	Piribedil hydrochloride	49.72283 ± 11.84560	-582.41337 ± 137.61183
13C02	Nitrocaramiphen hydrochloride	-20.17429 ± 9.86256	-120.26111 ± 42.24670
13C03	Nandrolone	-10.78644 ± 17.46210	9.62833 ± 9.43307
13C04	Dimaprit dihydrochloride	6.55770 ± 7.50419	-20.98364 ± 30.63891
13C05	Oxfendazol	-5.36525 ± 12.32968	36.00132 ± 2.44495
13C06	Guaiacol	6.51685 ± 10.41473	29.73184 ± 13.00440
13C07	Capecitabine	-38.12829 ± 20.09110	32.06930 ± 15.10085
13C08	Pramipexole dihydrochloride	6.08756 ± 8.11017	-376.07721 ± 18.47790
13C09	Norgestimate	45.81632 ± 6.06888	-541.78816 ± 162.23535
13C10	Chlormadinone acetate	35.79396 ± 9.86388	-425.59088 ± 85.91958
13C11	Phenylbutazone	38.07187 ± 0.73970	-461.25403 ± 68.62516
13D02	Gliquidone	9.80574 ± 29.95081	-148.98398 ± 30.58974
13D03	Pizotifen malate	40.13397 ± 2.34234	-118.91062 ± 26.13194
13D04	Ribavirin	4.98949 ± 2.32083	5.96231 ± 5.65626
13D05	Cyclopenthiazide	-1.24006 ± 26.42201	5.32676 ± 0.15773
13D06	Fluvoxamine maleate	-5.83612 ± 7.37445	4.55787 ± 0.79016
13D07	Prothionamide	16.55379 ± 21.09978	3.60808 ± 0.55410
13D08	Fluticasone propionate	-12.16488 ± 14.64289	-738.78754 ± 50.62837
13D09	Zuclopenthixol dihydrochloride	52.01225 ± 6.29192	-729.31076 ± 127.77282
13D10	Proguanil hydrochloride	31.55667 ± 4.48407	-581.14952 ± 105.89337
13D11	Lymecycline	40.52391 ± 1.96971	-570.89168 ± 45.56704
13E02	Alfadolone acetate	26.70969 ± 23.95355	-227.91043 ± 48.69827
13E03	Alfaxalone	21.25808 ± 23.38941	-145.96167 ± 39.10181
13E04	Azapropazone	4.34091 ± 19.38790	40.74514 ± 9.79851
13E05	Meptazinol hydrochloride	24.77047 ± 30.01426	48.30270 ± 7.36367
13E06	Apramycin	-9.71592 ± 13.69022	48.13286 ± 4.67746
13E07	Darunavir	2.93403 ± 17.26447	39.75943 ± 4.22110
13E08	Fursultiamine Hydrochloride	24.74522 ± 18.28912	-524.08266 ± 36.92159
13E09	Gabexate mesilate	31.15232 ± 8.35178	-681.08622 ± 156.37907
13E10	Pivampicillin	23.79842 ± 12.18565	-684.98660 ± 77.36235
13E11	Lodoxamide	31.99923 ± 11.43119	32.73921 ± 2.17963
13F02	Flucloxacillin sodium	-3.51987 ± 27.24543	-190.19749 ± 33.48030
13F03	Trapidil	-25.12009 ± 21.48017	-233.66846 ± 28.25726
13F04	Deptropine citrate	-5.11507 ± 24.19524	40.89116 ± 11.25800
13F05	Sertraline	33.88079 ± 11.68172	35.10641 ± 15.15818
13F06	Ethamsylate	-18.35806 ± 30.71680	44.92796 ± 4.74524

13F07	Moxonidine	12.79317 ± 9.36375	16.96510 ± 12.40554
13F08	Etilefrine hydrochloride	-7.50545 ± 15.38435	-773.37948 ± 30.57050
13F09	Alprostadil	34.68440 ± 9.15800	-675.74118 ± 66.22570
13F10	Tribenoside	53.49998 ± 10.45044	-729.55127 ± 107.57128
13F11	Rimexolone	42.17656 ± 14.38262	19.57533 ± 2.91351
13G02	Isradipine	23.50518 ± 13.22382	13.14546 ± 32.39644
13G03	Nifekalant	-4.60727 ± 33.70890	-31.47616 ± 6.90167
13G04	Isometheptene mucate	14.54571 ± 21.65102	42.34738 ± 5.65514
13G05	Nifurtimox	2.73364 ± 19.99248	40.68316 ± 17.36518
13G06	Letrozole	5.49514 ± 20.37284	27.36326 ± 14.40481
13G07	Levofloxacin	-11.73053 ± 17.82610	42.41172 ± 7.15476
13G08	Tocainide hydrochloride	11.59074 ± 4.56602	-748.12637 ± 64.05710
13G09	Benzathine benzylpenicillin	25.52951 ± 6.57576	-544.50301 ± 48.42206
13G10	Risperidone	42.17147 ± 23.28254	-724.90992 ± 111.55058
13G11	Torsemide	42.72883 ± 7.10738	22.56798 ± 1.13678
13H02	Halofantrine hydrochloride	21.04919 ± 10.48981	9.14194 ± 29.49843
13H03	Articaine hydrochloride	18.92891 ± 8.72295	-246.23057 ± 14.60843
13H04	Nomegestrol acetate	44.20164 ± 4.76980	49.60317 ± 5.24615
13H05	Pancuronium bromide	-5.01256 ± 21.50032	38.66212 ± 16.71341
13H06	Molindone hydrochloride	46.32631 ± 3.53620	27.21715 ± 13.18586
13H07	Alcuronium chloride	26.73667 ± 25.43078	35.72529 ± 16.92415
13H08	Zalcitabine	12.31405 ± 14.93912	-85.60095 ± 13.97497
13H09	Methyldopate hydrochloride	7.18647 ± 5.32606	-70.87861 ± 19.69025
13H10	Levocabastine hydrochloride	50.88943 ± 5.57898	-648.79656 ± 83.98843
13H11	Pyrvinium pamoate	-363.83678 ± 16.76058	-239.46424 ± 16.32613
14A02	Etomidate	9.37914 ± 47.29116	41.96055 ± 16.94096
14A03	Tridihexethyl chloride	1.67863 ± 32.69945	22.75089 ± 0.86912
14A04	Penbutolol sulfate	6.21834 ± 17.75224	25.62130 ± 0.51380
14A05	Prednicarbate	-1.88922 ± 12.92938	22.45719 ± 1.21529
14A06	Sertaconazole nitrate	12.03001 ± 8.53846	21.73985 ± 7.14422
14A07	Repaglinide	1.73778 ± 17.98717	25.81377 ± 0.83470
14A08	Piretanide	44.55021 ± 25.79843	17.44033 ± 2.76779
14A09	Piperacetazine	35.02549 ± 15.52479	-369.16261 ± 10.02363
14A10	Oxyphenbutazone	-48.19552 ± 9.14681	-219.33127 ± 46.05423
14A11	Quinethazone	-99.13145 ± 22.24430	-176.03263 ± 23.19135
14B02	Moricizine hydrochloride	29.54816 ± 29.44989	20.91704 ± 4.03093
14B03	Iopanoic acid	37.54764 ± 22.60742	25.16642 ± 3.06143
14B04	Pivmecillinam hydrochloride	39.53683 ± 46.78474	21.47728 ± 2.05632
14B05	Levopropoxyphene napsylate	39.03337 ± 25.18345	22.97380 ± 3.68753
14B06	Piperidolate hydrochloride	35.02549 ± 15.52479	23.89685 ± 0.56921
14B07	Trifluridine	-61.79209 ± 36.83050	3.52403 ± 23.64607
14B08	Oxprenolol hydrochloride	33.51731 ± 29.04692	23.43310 ± 3.65794
14B09	Ondansetron Hydrochloride	54.09186 ± 8.99215	-72.87547 ± 18.83696
14B10	Propoxycaine hydrochloride	-51.20221 ± 37.50400	-116.45583 ± 32.27293
14B11	Oxaprozin	23.24874 ± 7.13794	-98.04455 ± 16.74914
14C02	Phensuximide	38.12249 ± 32.61951	21.43687 ± 3.68841
14C03	Ioxaglic acid	10.44602 ± 25.85754	21.93963 ± 2.64303
14C04	Naftifine hydrochloride	18.81822 ± 20.05030	19.59498 ± 1.34556
14C05	Meprylcaine hydrochloride	48.69864 ± 35.14403	36.01071 ± 14.01066
14C06	Milrinone	-43.24521 ± 18.04897	20.01425 ± 3.35991
14C07	Methantheline bromide	-1.03240 ± 23.96607	16.90034 ± 2.58705

14C08	Ticarcillin sodium	19.36642 ± 15.27459	27.44763 ± 3.83683
14C09	Thiethylperazine dimalate	-0.93556± 32.35232	-80.37835 ± 17.40951
14C10	Mesalamine	10.71943 ± 10.43321	-98.07666 ± 22.41974
14C11	Vorinostat	-148.37400 ± 38.37738	-156.15221 ± 5.57515
14D02	Imidurea	55.88363 ± 18.94122	22.44950 ± 2.20213
14D03	Lansoprazole	29.48875 ± 19.92214	23.65216 ± 1.75467
14D04	Bethanechol chloride	60.96420 ± 15.48961	89.71932 ± 17.75146
14D05	Cyproterone acetate	71.32670 ± 13.40178	72.40849 ± 13.90352
14D06	(R)-Propranolol hydrochloride	-22.33358 ± 19.24254	23.12396 ± 1.73385
14D07	Ciprofibrate	56.30488 ± 29.43247	20.42089 ± 4.11144
14D08	Formestane	32.54557 ± 21.37846	27.37168 ± 3.14160
14D09	Benzylpenicillin sodium	83.60704 ± 10.01218	-13.38097 ± 27.44348
14D10	Methicillin sodium	18.47693 ± 20.54518	-53.37822 ± 21.51342
14D11	Methiazole	-470.34515 ± 61.29092	-625.07028 ± 8.92326
14E02	(S)-propranolol hydrochloride	71.50897 ± 8.55291	23.82809 ± 3.96359
14E03	(-)-Eseroline fumarate salt	-135.30331 ± 58.78872	87.36676 ± 27.82144
14E04	Isosorbide mononitrate	15.32553 ± 9.21957	24.27406 ± 3.44285
14E05	Levalbuterol hydrochloride	21.74546 ± 21.09720	30.14482 ± 0.40475
14E06	Topiramate	-23.41755 ± 25.00117	27.73240 ± 3.15261
14E07	D-cycloserine	-26.92694 ± 39.22919	27.91879 ± 2.38316
14E08	Nelarabine	-5.69772 ± 11.86629	-242.20767 ± 38.76902
14E09	(+,-)-Synephrine	-46.82535 ± 17.06820	5.66572 ± 13.93725
14E10	(S)-(-)-Cycloserine	-56.56203 ± 19.95571	-13.30788 ± 15.29666
14E11	Homosalate	50.30143 ± 5.46508	-40.13908 ± 15.51293
14F02	Spaglumic acid	42.52336 ± 27.24270	26.54187 ± 2.26073
14F03	Ranolazine	2.11105 ± 34.28176	27.32616 ± 1.26389
14F04	Misoprostol	31.78798 ± 12.15378	27.22895 ± 1.06920
14F05	Sulfadoxine	56.13991 ± 15.24332	81.25958 ± 57.86355
14F06	Cyclopentolate hydrochloride	16.87365 ± 22.85880	27.68308 ± 2.92354
14F07	Estriol	97.63027 ± 20.23128	98.28503 ± 21.88486
14F08	(-)-Isoproterenol hydrochloride	-45.53821 ± 30.48131	-111.14287 ± 12.01790
14F09	Sarafloxacin	-19.19238 ± 26.44858	52.68230 ± 15.06381
14F10	Nialamide	-88.30271 ± 35.04305	-2.32479 ± 26.61504
14F11	Toltrazuril	-199.82889 ± 25.79308	-16.98964 ± 12.00925
14G02	Perindopril	74.74189 ± 18.25871	72.72440 ± 17.30851
14G03	Fexofenadine hydrochloride	25.80532 ± 11.40569	22.61397 ± 4.91873
14G04	4-aminosalicylic acid	53.56886 ± 21.66663	28.12202 ± 0.81012
14G05	Clonixin Lysinate	23.23102 ± 26.24224	31.58091 ± 1.11437
14G06	Verteporfin	-55.58399 ± 82.82262	23.66644 ± 3.74256
14G07	Meropenem	8.68006 ± 26.50345	28.92838 ± 2.05794
14G08	Ramipril	6.61183 ± 13.12230	-119.20784 ± 23.20088
14G09	Mephenytoin	47.10734 ± 34.16534	57.37391 ± 12.85079
14G10	Rifabutin	-64.63985 ± 16.67238	39.29681 ± 18.65087
14G11	Parbendazole	-98.15977 ± 13.75613	-216.06348 ± 28.34570
14H02	Mecamylamine hydrochloride	95.36093 ± 6.74201	63.73187 ± 11.96024
14H03	Procarbazine hydrochloride	40.24288 ± 25.76398	24.05121 ± 6.48627
14H04	Viomycin sulfate	62.50190 ± 8.50944	82.20798 ± 24.28749
14H05	Saquinavir mesylate	-8.08427 ± 6.94212	31.02240 ± 2.81778
14H06	Ronidazole	86.81512 ± 6.54091	58.80820 ± 18.38216
14H07	Dorzolamide hydrochloride	2.41702 ± 26.06043	32.24176 ± 0.31571
14H08	Azaperone	42.91506 ± 14.27362	-89.56160 ± 19.80856

14H09	Cefepime hydrochloride	-58.97240 ± 29.41612	28.85975 ± 18.93001
14H10	Clocortolone pivalate	5.56925 ± 49.60466	53.61474 ± 6.05638
14H11	Nadifloxacin	-15.58493 ± 17.52155	36.21564 ± 24.35534
15A02	Buspirone hydrochloride	-64.44401 ± 24.41783	-224.79136 ± 20.69514
15A03	Anastrozole	-42.69782 ± 2.25828	-113.45904 ± 20.74326
15A04	Doxycycline hydrochloride	-13.24200± 39.87225	-193.34462 ± 16.43397
15A05	Sulbactam	13.74905 ± 3.93033	-154.24744 ± 17.01230
15A06	Fleroxacin	81.77111 ± 15.58285	-31.81956 ± 27.18452
15A07	Clavulanate potassium salt	41.17378 ± 5.51675	-107.32601 ± 19.30336
15A08	Valproic acid	69.54519 ± 15.75184	-127.82151 ± 12.93850
15A09	Mepivacaine hydrochloride	-109.69930 ± 83.14136	-168.04163 ± 62.10210
15A10	Rifaximin	-22.30443 ± 43.23949	-236.08678 ± 57.91522
15A11	Estradiol Valerate	-132.42160 ± 50.26407	-187.42984 ± 44.37326
15B02	Acetylcysteine	-32.77203 ± 21.71730	-104.75570 ± 12.34300
15B03	Melengestrol acetate	5.38641 ± 14.72297	-71.58759 ± 21.95648
15B04	Bromhexine hydrochloride	49.34145 ± 38.07018	-62.94937 ± 13.20268
15B05	Anethole-trithione	38.56720 ± 11.97696	-157.09519 ± 22.64850
15B06	Amcinonide	67.08230 ± 14.79560	8.72113 ± 39.64593
15B07	Caffeine	55.27766 ± 28.41829	-33.60934 ± 10.45559
15B08	Carvedilol	58.98954 ± 10.04318	-25.50484 ± 18.60982
15B09	Methenamine	11.70197 ± 30.29893	-161.28302 ± 9.52022
15B10	Phentermine hydrochloride	46.54637 ± 20.47418	-175.73883 ± 45.09172
15B11	Diclazuril	12.52661 ± 44.98826	-166.74402 ± 93.42453
15C02	Famciclovir	7.06510 ± 7.96539	-92.01467 ± 28.61502
15C03	Dopamine hydrochloride	87.47729 ± 20.07739	-40.06099 ± 9.75662
15C04	Cefdinir	36.11558 ± 14.81901	-72.89310 ± 4.52434
15C05	Carprofen	-31.87150 ± 24.95693	-80.44715 ± 11.36600
15C06	Celecoxib	-19.89139 ± 32.81391	1.85088 ± 21.53337
15C07	Candesartan	30.71395 ± 18.03010	-34.37692 ± 19.84992
15C08	Fludarabine	-108.23384 ± 71.78274	-249.67746 ± 31.65103
15C09	Cladribine	-13.70133± 20.08392	-142.02580 ± 25.54562
15C10	Vardenafil	-228.38980± 43.60372	-209.40365 ± 55.70697
15C11	Fluconazole	-61.84164 ± 49.12089	-45.22083 ± 5.40837
15D02	5-fluorouracil	-579.10788 ± 50.37924	-162.41822 ± 24.69683
15D03	Mesna	109.27575 ± 12.69283	-20.48063 ± 14.64758
15D04	Mitotane	4.18302 ± 13.03412	-63.29666 ± 8.75496
15D05	Ambrisentan	-10.20666 ± 35.71362	-72.96678 ± 29.21252
15D06	Triclosan	-14.91812 ± 24.81174	20.25240 ± 37.88314
15D07	Enoxacin	42.05637 ± 12.89054	8.92574 ± 3.06188
15D08	Olopatadine hydrochloride	-1.48507 ± 32.39343	21.32009 ± 9.40770
15D09	Granisetron	41.95066 ± 6.16662	-44.70070 ± 34.57505
15D10	Anthralin	16.11695± 2.14410	-86.51185 ± 69.17613
15D11	Lamotrigine	2.85413± 8.40496	-14.50423 ± 46.52809
15E02	Clofibrate	58.04934 ± 6.16662	-2.79434 ± 14.91942
15E03	Dexrazoxane hydrochloride	-1.25947 ± 41.71998	-32.19784 ± 17.82837
15E04	Aripiprazole	9.27419± 25.77170	-8.48924 ± 23.72038
15E05	Ethinylestradiol	-105.17262 ± 63.55656	-291.22741 ± 109.23235
15E06	Fluocinolone acetonide	0.19881 ± 4.56739	35.95570 ± 13.96686
15E07	Sparfloxacin	48.96629 ± 13.89560	19.02267 ± 13.24209
15E08	Desloratadine	-386.93244 ± 15.68907	8.94445 ± 5.15631
15E09	Clarithromycin	-26.53407 ± 62.19951	-36.64393 ± 33.11479

15E10	Tripelennamine hydrochloride	-47.90347 ± 17.05210	-22.30736 ± 4.99200
15E11	Tulobuterol	-15.51918 ± 48.21759	-44.80083 ± 51.55280
15F02	Topotecan	-199.82889 ± 25.79308	-398.55267 ± 50.94970
15F03	Atorvastatin	-53.02101 ± 55.51645	-22.50314 ± 6.48886
15F04	Azithromycin	9.27419± 25.77170	-34.00011 ± 1.91043
15F05	Ibudilast	48.29994 ± 36.07216	-52.71206 ± 17.19437
15F06	Losartan	-37.22498 ± 46.96397	41.27721 ± 16.14010
15F07	Benztropine mesylate	59.01240 ± 10.80801	44.93958 ± 5.05295
15F08	Vecuronium bromide	-14.01703 ± 9.78115	34.82901 ± 6.97858
15F09	Telmisartan	-256.80050 ± 144.89447	6.62230 ± 16.04025
15F10	Nalmefene hydrochloride	46.18300 ± 42.58773	31.07641 ± 11.52003
15F11	Bifonazole	-60.07704 ± 48.67890	-1.58996 ± 13.38990
15G02	Gatifloxacin	60.76555 ± 19.75719	62.55421 ± 13.50713
15G03	Bosentan	11.57729 ± 8.60423	24.65530 ± 9.42999
15G04	Gemcitabine	-641.10059± 270.76849	-500.06552 ± 75.01186
15G05	Olmesartan	-17.82347 ± 21.98550	-31.71907 ± 14.66751
15G06	Racepinephrine hydrochloride	39.81838 ± 21.94392	28.74719 ± 18.77630
15G07	Montelukast	52.08440 ± 24.55024	31.06654 ± 20.86974
15G08	Docetaxel	-102.80241 ± 13.78102	-117.66338 ± 21.60588
15G09	Cilnidipine	-116.21222 ± 68.89683	70.58174 ± 12.47965
15G10	Imiquimod	-141.47115 ± 46.76469	63.31032 ± 49.73189
15G11	Fosinopril	-367.24385 ± 24.99988	23.25373 ± 22.99786
15H02	Imatinib	37.62014 ± 16.44880	9.86564 ± 20.09313
15H03	Moxifloxacin	43.50369 ± 7.53835	59.78554 ± 16.22150
15H04	Formoterol fumarate	80.14461 ± 19.04598	37.78696 ± 7.60015
15H05	Rufloxacin	53.52169 ± 11.17318	-1.55204 ± 8.76282
15H06	Pravastatin	70.21681 ± 9.89258	90.57085 ± 9.21074
15H07	Rosiglitazone Hydrochloride	96.81577 ± 10.75302	40.60824 ± 12.07182
15H08	Rivastigmine	-70.95116 ± 44.12276	55.02370 ± 11.48291
15H09	Sildenafil	10.36769 ± 22.91938	41.89928 ± 22.76579
15H10	Acetylsalicylic acid	-105.41919 ± 68.24800	3.94360 ± 10.43069
15H11	Hexachlorophene	-300.90267 ± 20.84057	12.49510 ± 8.95325
16A02	Nelfinavir mesylate	75.51313 ± 8.01445	-359.44376 ± 48.26266
16A03	Silodosin	74.14550 ± 5.81554	-194.94590 ± 47.37309
16A04	Trimebutine	24.37592 ± 4.07723	-58.86497 ± 23.33522
16A05	Nevirapine	28.58624± 8.80604	31.16722 ± 30.23644
16A06	Doxapram hydrochloride	62.99360 ± 8.70983	-251.23961 ± 31.81218
16A07	Amlexanox	28.51533 ± 10.00028	-239.07163 ± 14.46922
16A08	Amorolfine hydrochloride	42.33098 ± 17.71984	-198.22597 ± 19.41145
16A09	Enrofloxacin	-19.63554 ± 13.89726	-266.70571 ± 125.21894
16A10	Ubenimex	17.69107 ± 12.44837	-226.29342 ± 13.50736
16A11	Troxipide	49.94191 ± 5.14595	-271.50742 ± 47.92027
16B02	Ipriflavone	33.35165 ± 13.48014	-30.15333 ± 23.84251
16B03	Ezetimibe	50.06043 ± 7.26586	-142.45276 ± 51.58064
16B04	Rizatriptan benzoate	32.09741 ± 8.87015	-18.81718 ± 7.16733
16B05	Tegaserod maleate	-5.33804 ± 29.46640	-108.57001 ± 46.33709
16B06	Pantoprazole sodium	37.12600 ± 19.91249	-137.45324 ± 8.66524
16B07	Tegafur	57.79360 ± 7.34173	-158.63707 ± 9.46598
16B08	Tolcapone	56.07817 ± 11.89448	-181.74779 ± 23.98822
16B09	Altrenogest	20.86252 ± 12.66199	-148.04369 ± 15.46320
16B10	Felbamate	-23.31706 ± 1.93673	-151.14460 ± 30.35037

16B11	Estramustine	-334.87769 ± 120.38515	-163.13975 ± 22.10088
16C02	(R)-Duloxetine hydrochloride	62.37650 ± 9.35721	-456.14992 ± 115.14573
16C03	Donepezil hydrochloride	31.24070 ± 9.38674	-505.12973 ± 18.42169
16C04	1,8-Dihydroxyanthraquinone	14.73535 ± 10.36962	-376.60711 ± 15.04326
16C05	Nitazoxanide	42.40837 ± 8.14829	-828.80525 ± 52.07680
16C06	Nateglinide	17.57633 ± 10.11572	-145.83939 ± 23.96756
16C07	Avobenzone	2.79487 ± 3.75218	-108.44192 ± 17.07682
16C08	Algestone acetophenide	16.07950 ± 10.89640	-75.99342 ± 15.02323
16C09	Actarit	22.66178 ± 8.47172	-169.41844 ± 3.97286
16C10	Ethoxzolamide	-19.08472 ± 13.70715	-152.34571 ± 9.22533
16C11	Azatadine maleate	4.33189 ± 4.21294	-139.58587 ± 12.58897
16D02	Aminacrine	-187.16330 ± 68.95499	-211.09212 ± 86.60227
16D03	Pidotimod	0.23668 ± 3.26446	-293.63149 ± 39.87782
16D04	Benidipine hydrochloride	41.05308 ± 10.15865	-171.79974 ± 67.85575
16D05	Perospirone	-43.35596 ± 30.21675	-743.62185 ± 226.52596
16D06	Cefpiramide	24.89326 ± 14.58648	-84.53116 ± 19.07754
16D07	Fenoldopam	52.04605 ± 3.22647	-78.29380 ± 18.22982
16D08	Adapalene	-420.94272 ± 86.86341	-61.21903 ± 20.33814
16D09	Diatrizoic acid dihydrate	75.10674 ± 14.58648	-46.62123 ± 5.94352
16D10	Dofetilide	11.20165 ± 18.07617	-86.62571 ± 9.20681
16D11	Phenprobamate	18.20628 ± 5.49519	-106.31560 ± 37.66007
16E02	Cefuroxime axetil	24.89326 ± 14.58648	-129.65371 ± 62.26822
16E03	Anagrelide	11.63878 ± 4.82783	-193.94840 ± 64.44976
16E04	Clopidogrel	24.81833 ± 19.05655	-157.48020 ± 22.99305
16E05	Benzoxiquine	-86.55751 ± 38.63912	-191.32530 ± 35.33679
16E06	Phenothiazine	50.42952 ± 7.18573	-54.51739 ± 13.59516
16E07	Enalaprilat dihydrate	-21.03744 ± 5.35416	-47.63129 ± 9.52949
16E08	Pregabalin	10.76097 ± 8.05810	-15.28293 ± 24.57719
16E09	Homoveratrylamine	1.69068 ± 13.59781	-63.23997 ± 27.61238
16E10	Zoledronic acid hydrate	-18.43244 ± 16.78310	-13.60918 ± 6.79576
16E11	Cefpodoxime proxetil	-45.55192 ± 7.59599	-35.96965 ± 4.88761
16F02	Irbesartan	-65.74475 ± 47.31587	-13.54241 ± 30.23962
16F03	Indinavir sulfate	-49.57722 ± 9.65868	-85.30652 ± 25.34055
16F04	Terbinafine	19.09234 ± 6.41995	-1.91386 ± 10.42954
16F05	Histamine dihydrochloride	21.81677 ± 6.29119	-168.67093 ± 8.98218
16F06	Rasagiline	-25.45347 ± 26.42021	-29.42486 ± 15.86925
16F07	Flumethasone pivalate	-26.15555 ± 5.84865	-11.21766 ± 9.79483
16F08	Lofepramine	53.23177 ± 5.58879	28.97895 ± 10.40842
16F09	Valdecoxib	0.99714 ± 29.64689	8.57285 ± 21.91551
16F10	Besifloxacin hydrochloride	-209.96359 ± 29.94006	65.57729 ± 11.25003
16F11	Ritonavir	-97.11270 ± 26.96835	-17.83498 ± 21.99113
16G02	Epirubicin hydrochloride	-261.98485 ± 188.82953	-464.04206 ± 260.34363
16G03	Loteprednol etabonate	-35.49677 ± 7.31455	-32.75372 ± 90.06527
16G04	Tolterodine tartrate	37.22710 ± 16.16067	26.01991 ± 30.17858
16G05	Lomerizine hydrochloride	40.27690 ± 18.54198	-98.62699 ± 27.60973
16G06	Ampiroxicam	3.80451 ± 4.05480	25.27550 ± 15.53328
16G07	Alosetron hydrochloride	-14.51545 ± 15.89944	0.71992 ± 32.08469
16G08	Risedronic acid monohydrate	19.03568 ± 10.90614	45.32012 ± 13.92228
16G09	Palonosetron hydrochloride	-16.25265 ± 28.53359	4.49348 ± 7.35409
16G10	Oxymetholone	-36.00755 ± 3.33328	-9.39119 ± 56.75334
16G11	Latanoprost	-23.63408 ± 37.71119	21.36767 ± 34.28279

16H02	Cisatracurium besylate	-40.69716 ± 18.48057	28.67097 ± 7.48810
16H03	Pemetrexed disodium	-41.12961 ± 11.68846	-19.42179 ± 20.49787
16H04	Raltitrexed	-205.07191 ± 19.34098	-47.65566 ± 35.81536
16H05	Ceftibuten	49.43311 ± 8.11193	-113.00795 ± 6.98546
16H06	Valsartan	22.84710 ± 19.95635	23.72771 ± 13.27089
16H07	Milnacipran hydrochloride	8.92951 ± 5.73744	-22.15793 ± 26.52642
16H08	Triclabendazole	28.25552 ± 4.69498	35.86300 ± 20.95654
16H09	Brimonidine L-Tartrate	13.85786 ± 9.29805	33.78196 ± 13.05931
16H10	Desonide	6.36018 ± 13.92030	12.88209 ± 37.52534
16H11	Cefprozil	-442.16134 ± 128.00100	45.69471 ± 12.41249

Supplementary Table 2. Secondary drug screening for inhibitory modifiers of α-synuclein propagation

HCS No.	Name	Normalized inhibition (%)
01A04	Acetazolamide	8.17557 ± 10.41480
01A05	Metformin hydrochloride*	46.17124 ± 7.09331
01A09	Amprolium hydrochloride	23.41166 ± 7.73530
01A10	Hydrochlorothiazide	17.58282 ± 8.29294
01A11	Sulfaguanidine	33.75510 ± 13.31750
01B10	Captopril	24.01194 ± 14.16562
01B11	Minoxidil	21.29622 ± 11.70115
02A02	Metronidazole	22.75932 ± 8.14595
02A03	Fulvestrant	35.62413 ± 0.94200
02A07	Acyclovir	6.66908 ± 9.34953
02A08	Diazoxide	30.78644 ± 8.94942
02B02	Khellin	2.85496 ± 9.71922
02B07	Guanabenz acetate	25.45288 ± 12.63587
02B09	Acetylsalicylsalicylic acid	3.27852 ± 25.60486
02C07	Nicorandil	18.43313 ± 10.99117
02C08	Pioglitazone	-126.98299 ± 15.15342
02C11	Oxandrolone*	45.14198 ± 12.38420
02D02	Naloxone hydrochloride	10.28969 ± 12.80263
02D04	Ciprofloxacin hydrochloride monohydrate	8.03209 ± 6.45978
02D05	Ampicillin trihydrate	36.75246 ± 7.71999
02D09	Nalbuphine hydrochloride	33.42054 ± 9.80830
02G02	Verapamil hydrochloride	-52.97132 ± 20.54148
02G11	Dihydroergotamine tartrate**	48.52938 ± 6.36318
02H02	Erythromycin	-17.33454 ± 9.52601
02H09	Neomycin sulfate**	48.42470 ± 1.67065
02H10	Dihydrostreptomycin sulfate**	50.65397 ± 9.39332
03A06	Ethosuximide***	56.12513 ± 4.07331
03C02	Ampyrone	37.63003 ± 6.66297
03C11	Tolbutamide*	43.79651 ± 1.02866
03E10	Metoclopramide monohydrochloride****	64.38270 ± 4.64141
04A05	Glutethimide, para-amino	46.63385 ± 11.47715
04B09	Antazoline hydrochloride	39.07710 ± 3.66575
04C04	Doxepin hydrochloride****	67.86659 ± 22.32084
04C09	Vinpocetine***	54.32562 ± 5.39616
04C11	Fendiline hydrochloride	38.81716 ± 4.15562
04D09	Cinnarizine*	45.61695 ± 3.55921
04D10	Methylprednisolone, 6-alpha	-8.55332 ± 3.97784
04D11	Quinidine hydrochloride monohydrate	12.39635 ± 9.79009
04E09	Spiperone	4.51103 ± 6.11554
04E10	Pyrilamine maleate	-44.92691 ± 5.41029
04E11	Sulfinpyrazone	-8.85080 ± 10.38534
04F09	Fipexide hydrochloride	-19.53770 ± 9.66882
04F10	Mifepristone	36.72993 ± 5.21590
04G08	Oxytetracycline dihydrate	7.13942 ± 1.17151
04G10	Amodiaquin dihydrochloride dihydrate	14.57344 ± 3.49583
04G11	Mebeverine hydrochloride	15.03420 ± 7.83510
04H08	Guanethidine sulfate*	45.20633 ± 11.46721
04H11	Fluphenazine dihydrochloride	4.73746 ± 14.25456
05A02	Streptomycin sulfate*	46.32601 ± 13.41777

05A10	Tacrine hydrochloride	-2.60193 ± 21.42547
05A11	Bisoprolol fumarate	20.76842 ± 6.00688
05B03	Practolol**	53.51776 ± 12.02881
05B07	Chlormezanone***	58.16702 ± 7.21718
05B10	Guanfacine hydrochloride	-41.50642 ± 6.95665
05B11	Domperidone	9.91747 ± 9.30545
05C03	Methapyrilene hydrochloride*	42.51570 ± 7.12530
05C06	Clenbuterol hydrochloride***	54.95133 ± 2.86249
05D03	Dobutamine hydrochloride**	50.94692 ± 5.02966
05D10	Dextromethorphan hydrobromide monohydrate**	52.40658 ± 8.87776
05E03	Betamethasone*	41.69414 ± 8.09872
05E06	Brinzolamide*	43.09016 ± 7.29473
05E07	Ambroxol hydrochloride***	54.82754 ± 2.35081
05E09	Bepridil hydrochloride****	64.11540 ± 9.77096
05E10	Meloxicam	-18.70007 ± 9.76720
05F07	Clofazimine	-9.86915 ± 13.19691
05F08	Nafronyl oxalate*	41.90540 ± 22.19429
05F09	Bezafibrate**	51.54339 ± 1.84811
05F10	Nefazodone hydrochloride	33.69628 ± 15.55155
05G04	Nicardipine hydrochloride****	61.40155 ± 26.88942
05G08	Carbetapentane citrate	42.77781 ± 14.28019
05G10	Ketoconazole	39.84936 ± 1.55839
05H10	Prochlorperazine dimaleate****	67.31284 ± 6.88775
06A05	Idebenone	-8.79078 ± 13.30707
06A08	Adamantamine fumarate**	54.11584 ± 2.04018
06A09	Butoconazole nitrate	18.31520 ± 25.07146
06B08	Lamivudine	31.69649 ± 5.05941
06B09	Biotin*	41.20723 ± 4.08887
06C06	Chicago sky blue 6B****	67.32207 ± 19.45831
06C09	Roxatidine Acetate hydrochloride	-7.83690 ± 20.70943
06D05	Cefadroxil	29.14621 ± 15.11925
06E06	Cyclobenzaprine hydrochloride	28.43143 ± 5.49881
06F06	Mebhydroline 1,5-naphtalenedisulfonate*	44.99692 ± 10.59279
06G05	Quetiapine hemifumarate	34.52822 ± 10.80318
06H05	Fentiazac	27.75686 ± 11.87370
06H07	Primaquine diphosphate	25.68073 ± 6.57387
07A07	Colistin sulfate	25.77946 ± 17.37355
07C08	Sibutramine hydrochloride*	45.04690 ± 0.30268
07D07	Lovastatin	21.47947 ± 11.63934
07E09	Triflusal**	51.45266 ± 10.14594
07G08	Molsidomine	32.71432 ± 10.24329
08A06	Atropine sulfate monohydrate	12.06987 ± 2.15720
08A07	Eserine hemisulfate salt	16.20264 ± 6.55903
08A08	Itraconazole	40.11784 ± 1.68934
08B07	Acetopromazine maleate salt***	55.75993 ± 6.99918
08B09	Ropinirole hydrochloride	14.84491 ± 6.36065
08C09	Galanthamine hydrobromide	15.00538 ± 4.39606
08D08	Temozolomide	34.25450 ± 4.55237
08D09	Xylazine	9.70834 ± 17.48563
08E06	Carbadox	-24.79987 ± 12.00901
08G05	Mercaptopurine	-49.30689 ± 9.50713

08H05	Tomoxetine hydrochloride	39.92646 ± 2.46238
08H06	Aceclidine Hydrochloride	22.55653 ± 5.53578
09A11	Pinaverium bromide***	56.18092 ± 3.85136
09H04	Dipyrone**	47.46238 ± 5.10040
09H05	Isosorbide dinitrate*	42.84240 ± 0.61094
09H10	Cephalothin sodium salt**	47.37825 ± 3.46259
10E02	Butamben*	44.99692 ± 10.59279
10G08	Merbromin****	100.00000 ± 0.00000
10H02	(R) -Naproxen sodium salt**	52.14157 ± 6.52265
11A04	Pyrithyldione***	56.84122 ± 4.60669
11D11	Ethopropazine hydrochloride**	51.38728 ± 10.06858
12A02	Niacin**	47.10597 ± 9.39765
12A08	Oxybenzone	33.26158 ± 10.95801
12A09	Promethazine hydrochloride	27.72649 ± 14.70770
12B06	Aminohippuric acid*	41.57597 ± 4.65742
12D06	Lorglumide sodium salt	16.53409 ± 4.48904
12F02	Propofol***	57.60025 ± 11.24151
12H02	Crotamiton	28.60360 ± 5.28514
12H05	Tyloxapol	27.16876 ± 6.68952
12H08	Deoxycorticosterone	36.46223 ± 5.38624
13A11	Indatraline hydrochloride*	46.49084 ± 3.71563
14D05	Cyproterone acetate*	45.80399 ± 1.26933
14D09	Benzylpenicillin sodium	26.05839 ± 6.14064
14E02	(S)-propranolol hydrochloride	2.50876 ± 12.81671
14F07	Estriol*	44.90876 ± 3.93080
14G02	Perindopril*	42.50891 ± 0.19076
14H02	Mecamylamine hydrochloride**	47.19780 ± 9.54345
14H06	Ronidazole*	46.47382 ± 7.24216
15A06	Fleroxacin	17.72449 ± 13.52147
15C03	Dopamine hydrochloride	32.45954 ± 9.02856
15D03	Mesna	27.15204 ± 9.12888
15H04	Formoterol fumarate	34.23249 ± 9.68730
15H06	Pravastatin****	63.77590 ± 1.99460
15H07	Rosiglitazone Hydrochloride	16.26169 ± 10.85767
16A02	Nelfinavir mesylate*	41.74381 ± 12.02671
16A03	Silodosin	35.40794 ± 9.72526
16A06	Doxapram hydrochloride	8.09699 ± 11.59692
16A11	Troxipide	18.97564 ± 4.94388
16B02	Ipriflavone	-9.95351 ± 44.46288
16B07	Tegafur	31.94442 ± 11.30386
16C02	(R)-Duloxetine hydrochloride	18.91831 ± 15.98197
16F08	Lofepramine*	46.86155 ± 10.54772

*; $P < 0.05$, **; $P < 0.005$, ***; $P < 0.0005$, ****; $P < 0.0001$ compared to V1S and SV2 co-cultured cells treated with DMSO

Supplementary Table 3. Secondary hit compound screening for inhibitory modifiers of mHtt propagation

HCS No.	Name	Normalized inhibition (%)
02H04	Didanosine	38.16381 ± 5.62296
02H10	Dihydrostreptomycin sulfate	36.93990 ± 3.46380
02H11	Gentamicine sulfate	21.74478 ± 4.87207
03A06	Ethosuximide	12.99829 ± 7.20601
03B03	Etofylline	-0.32288 ± 1.48451
03C03	Levamisole hydrochloride	1.52518 ± 4.49927
03C04	Pargyline hydrochloride	-4.44867 ± 3.37516
03C05	Methocarbamol	8.01028 ± 10.66335
03C11	Tolbutamide	39.63762 ± 3.91173
03G02	Norfloxacin	35.52196 ± 4.25261
04A05	Glutethimide, para-amino	24.68710 ± 2.77654
04A06	Dropropizine (R,S)[#]	52.85851 ± 1.20446
04B04	Norethindrone[####]	96.20292 ± 34.19172
04B06	Niflumic acid[###]	71.93302 ± 18.14026
04B08	Retinoic acid[##]	63.77801 ± 11.04232
04C09	Vinpocetine[##]	62.94828 ± 7.54248
04D07	Bumetanide[####]	102.21636 ± 23.67710
04D08	Labetalol hydrochloride[####]	91.19795 ± 5.37537
04D09	Cinnarizine[###]	66.71424 ± 9.74040
04E07	Perhexiline maleate[####]	118.22737 ± 9.23110
04E08	Oxybutynin chloride[####]	95.07433 ± 7.06623
04F07	Acemetacin[####]	131.59769 ± 15.27894
04G07	Clemastine fumarate[####]	106.19376 ± 10.40510
04G08	Oxytetracycline dihydrate[####]	105.82296 ± 5.61277
04G09	Pimozide[####]	103.25715 ± 21.88648
04H07	Glibenclamide[####]	134.55830 ± 3.27213
04H09	Quinacrine dihydrochloride hydrate[####]	120.34560 ± 26.44384
05A02	Streptomycin sulfate	45.85324 ± 2.93021
06C06	Chicago sky blue 6B[###]	69.29177 ± 2.73699
06H08	Progesterone	31.35516 ± 6.20353
09F03	Citalopram Hydrobromide[#]	51.01537 ± 2.21278
09H04	Dipyrone	45.17905 ± 2.15629
09H05	Isosorbide dinitrate	29.55172 ± 1.15735
10H02	(R) -Naproxen sodium salt	25.32835 ± 4.25746
14D04	Bethanechol chloride[##]	57.73932 ± 1.13242
14D05	Cyproterone acetate[##]	56.08548 ± 1.69271
14E03	(-)-Eseroline fumarate salt[#]	52.11720 ± 5.12153
14F07	Estriol[##]	57.80116 ± 1.70816
14G02	Perindopril[##]	60.25359 ± 1.09935
14H02	Mecamylamine hydrochloride[##]	58.09398 ± 4.25909
14H04	Viomycin sulfate	18.25348 ± 10.89833
14H06	Ronidazole[#]	50.69551 ± 2.50943
15G02	Gatifloxacin	45.73256 ± 7.66895
15H06	Pravastatin[###]	74.30994 ± 9.42101

#; $P < 0.05$, ##; $P < 0.005$, ###; $P < 0.0005$, ####; $P < 0.0001$ compared to V1Q and QV2 co-cultured cells treated with DMSO
