# Supplementary material for: Statins suppress cell-to-cell propagation of α-synuclein by lowering cholesterol
Source: Cell Death Dis. 2023 Jul 27;14(7):474. doi: 10.1038/s41419-023-05977-9 (PMC10374525; doi:10.1038/s41419-023-05977-9)

Original Data

Figure 3A

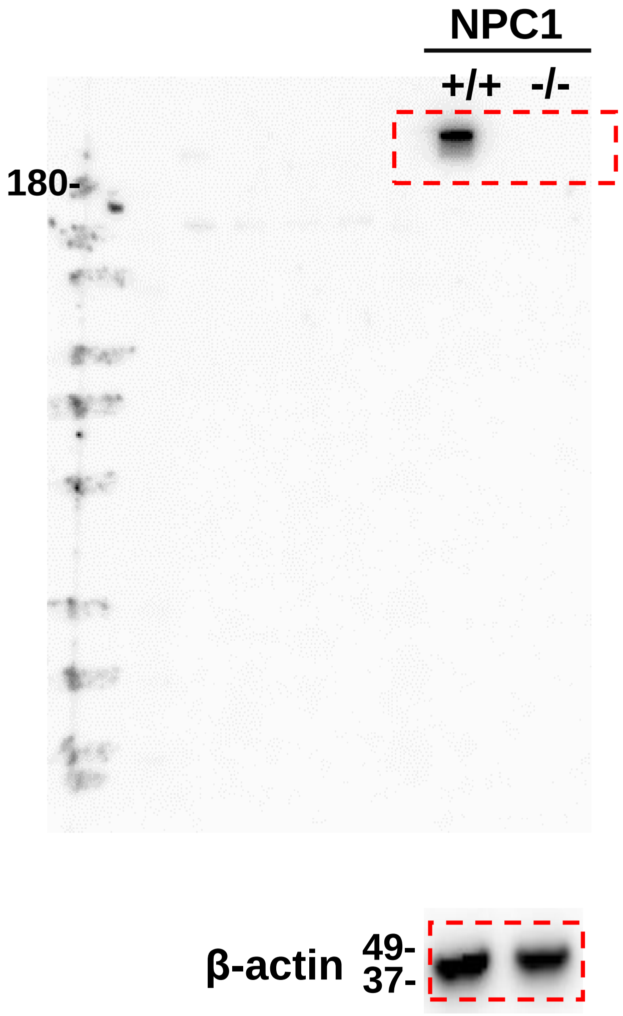

Figure 4A

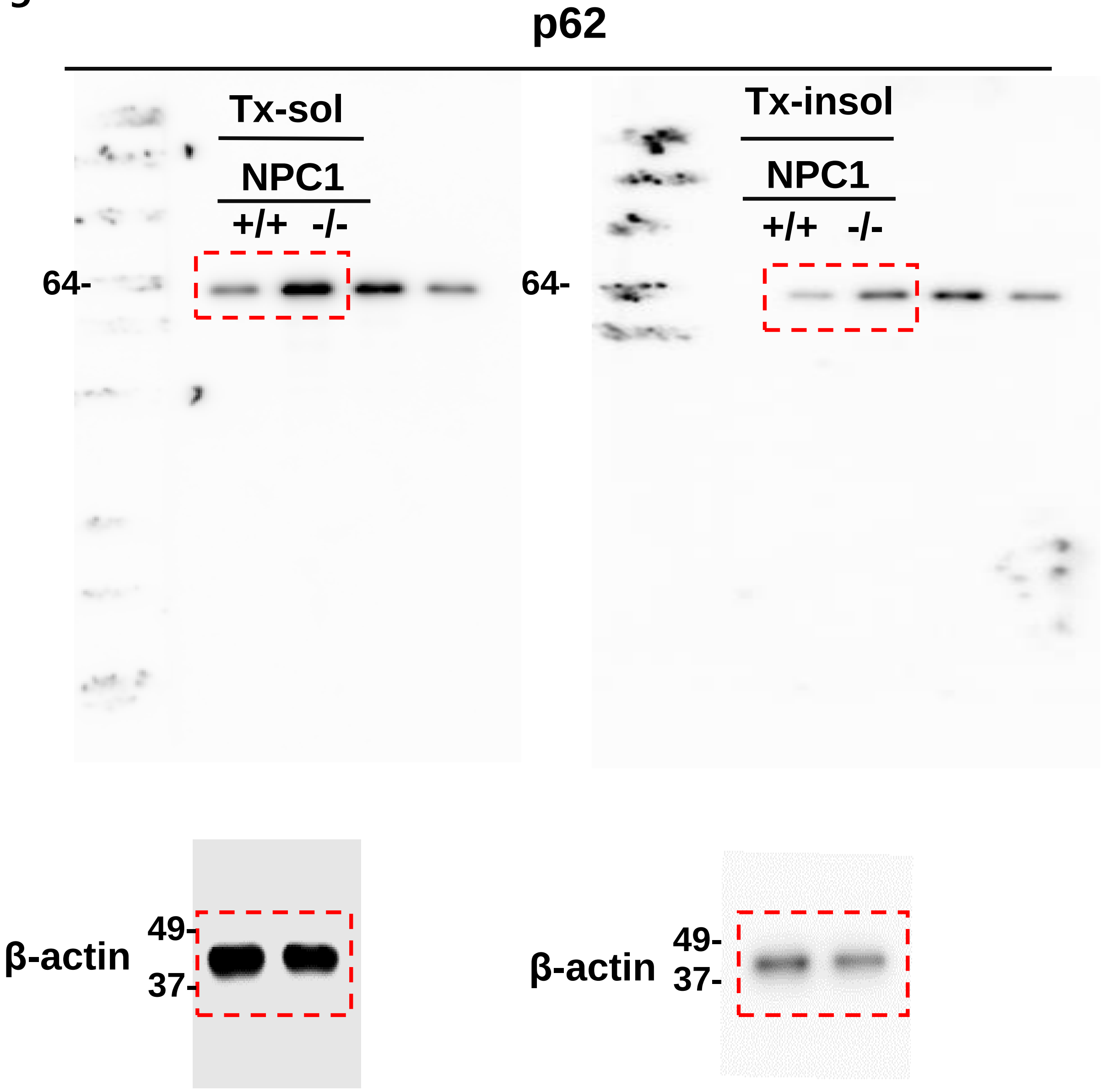

Figure 4B

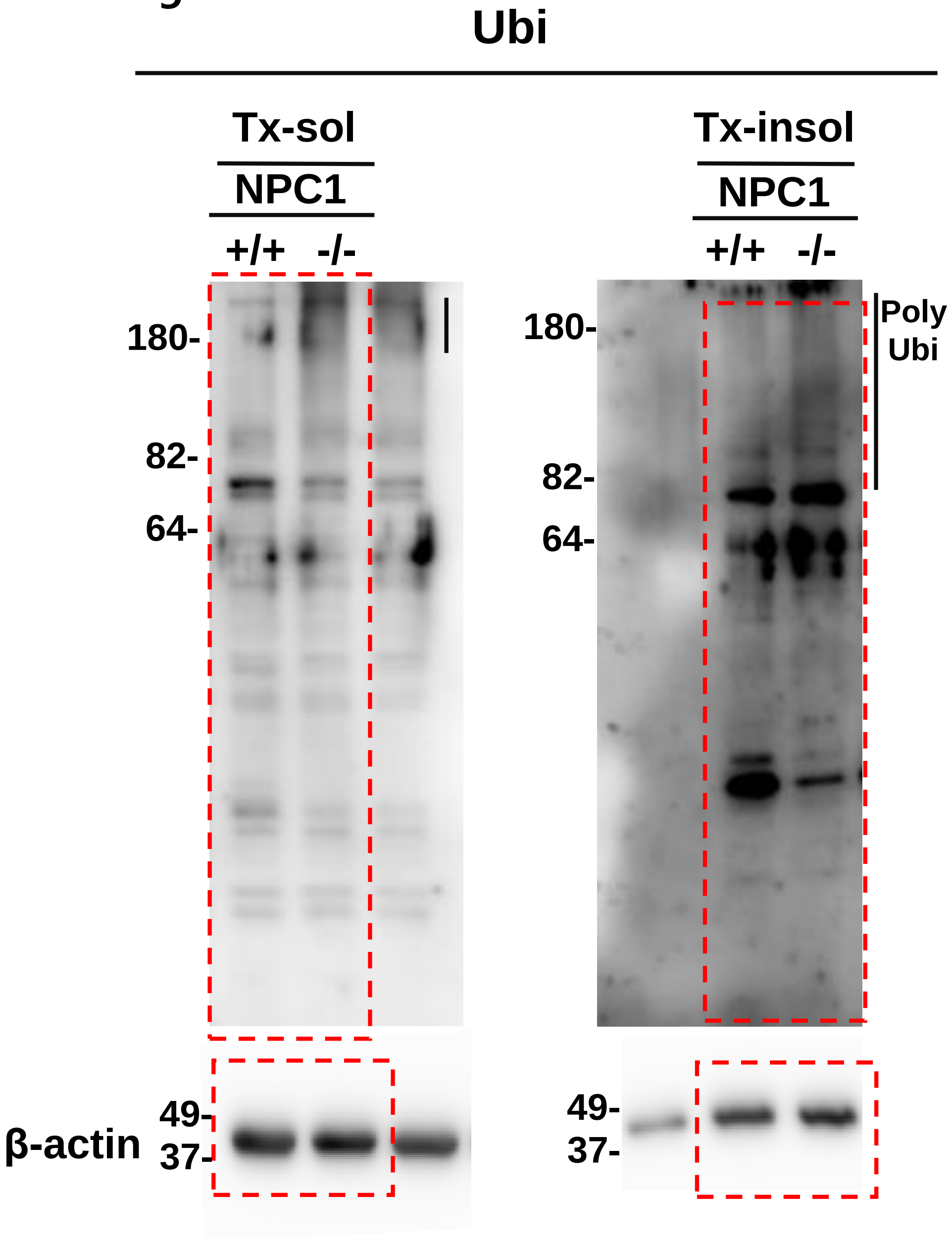

Figure 4C

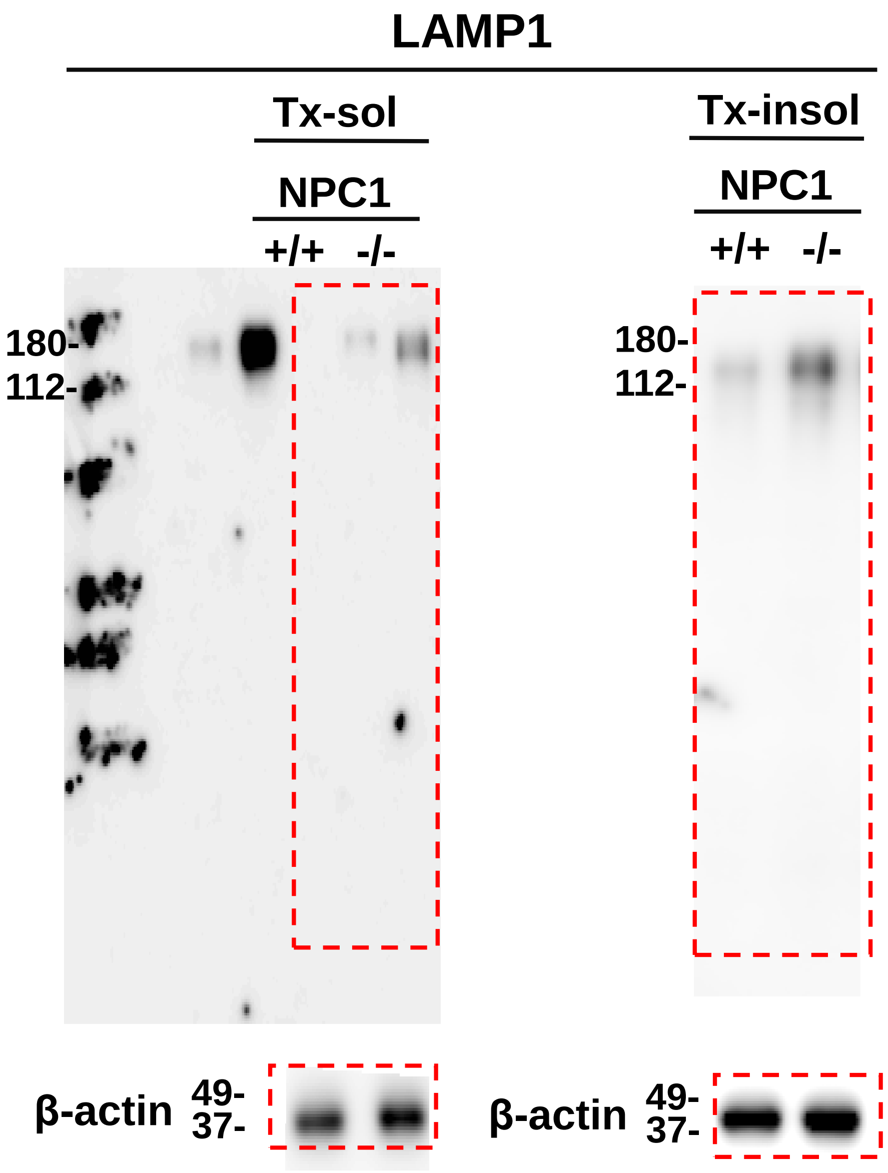

Figure 4E

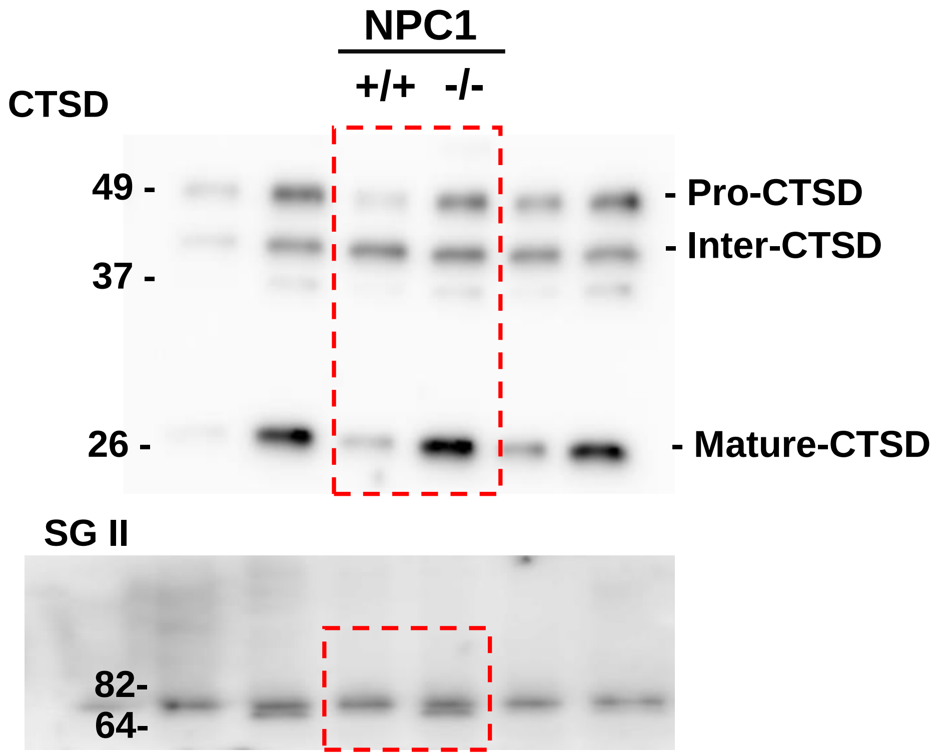

Figure 5A

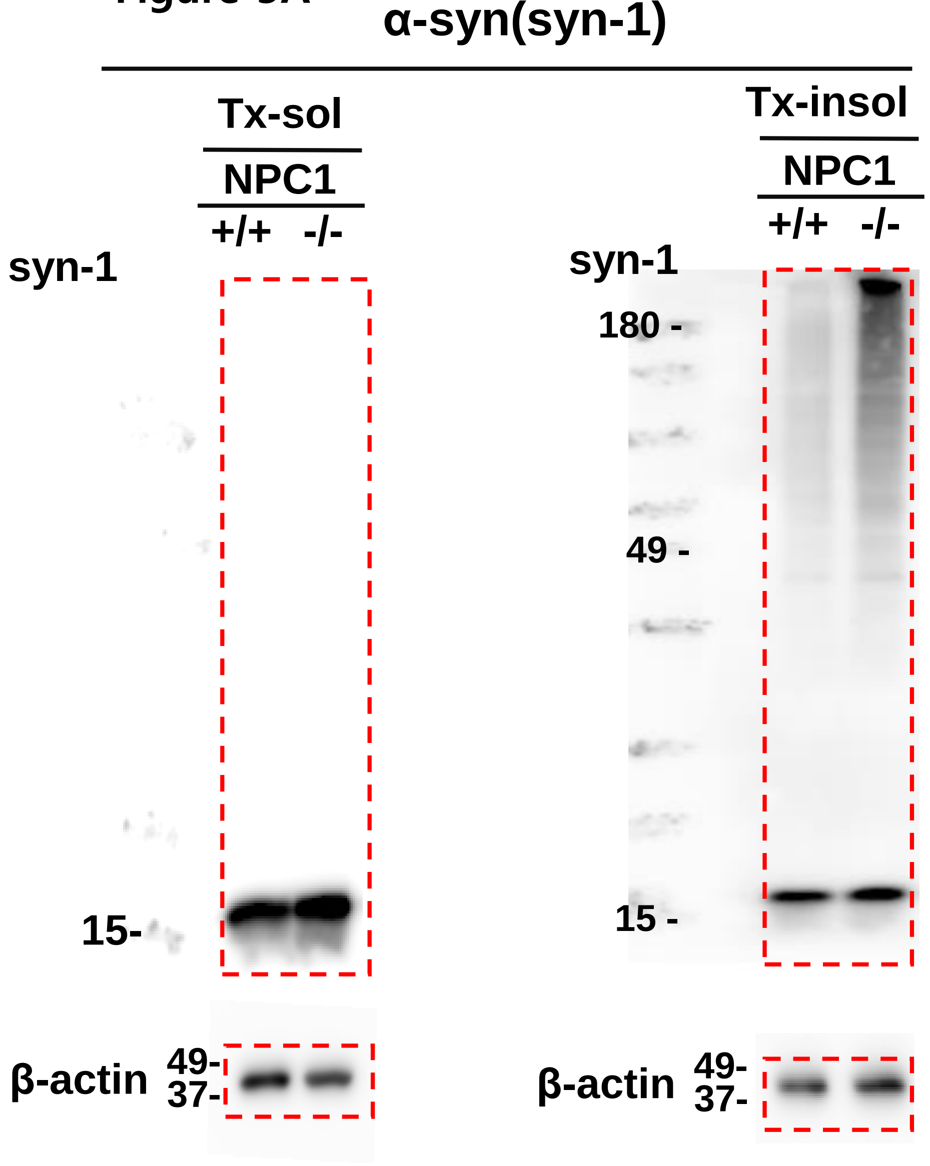

Figure 5B

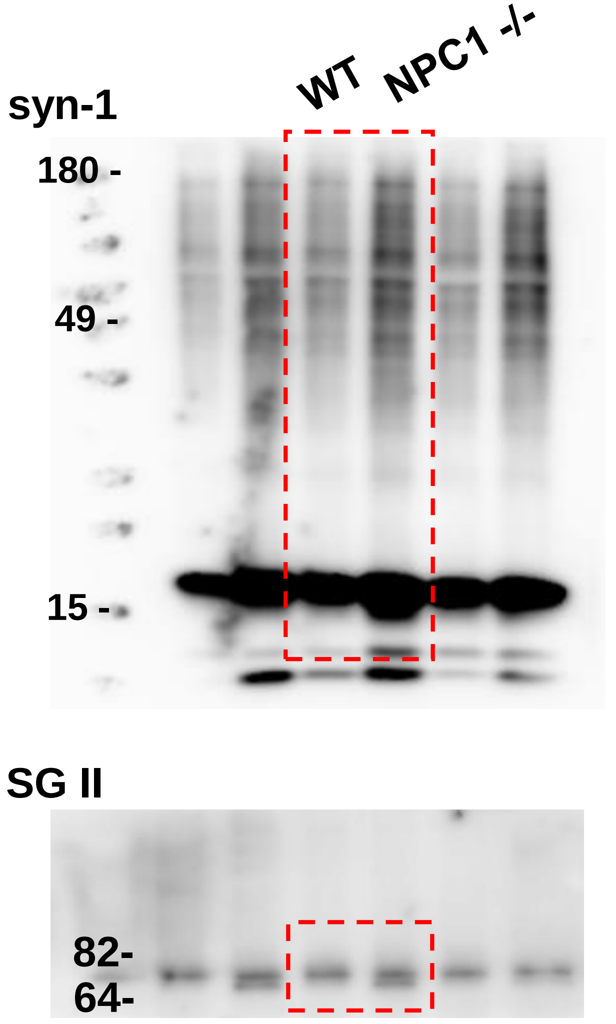

Figure 5D

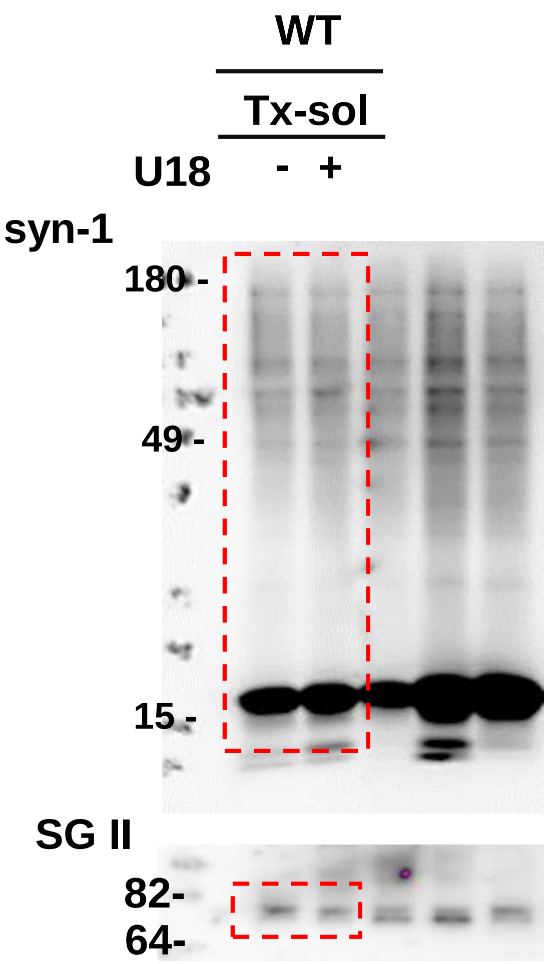

Figure 5E

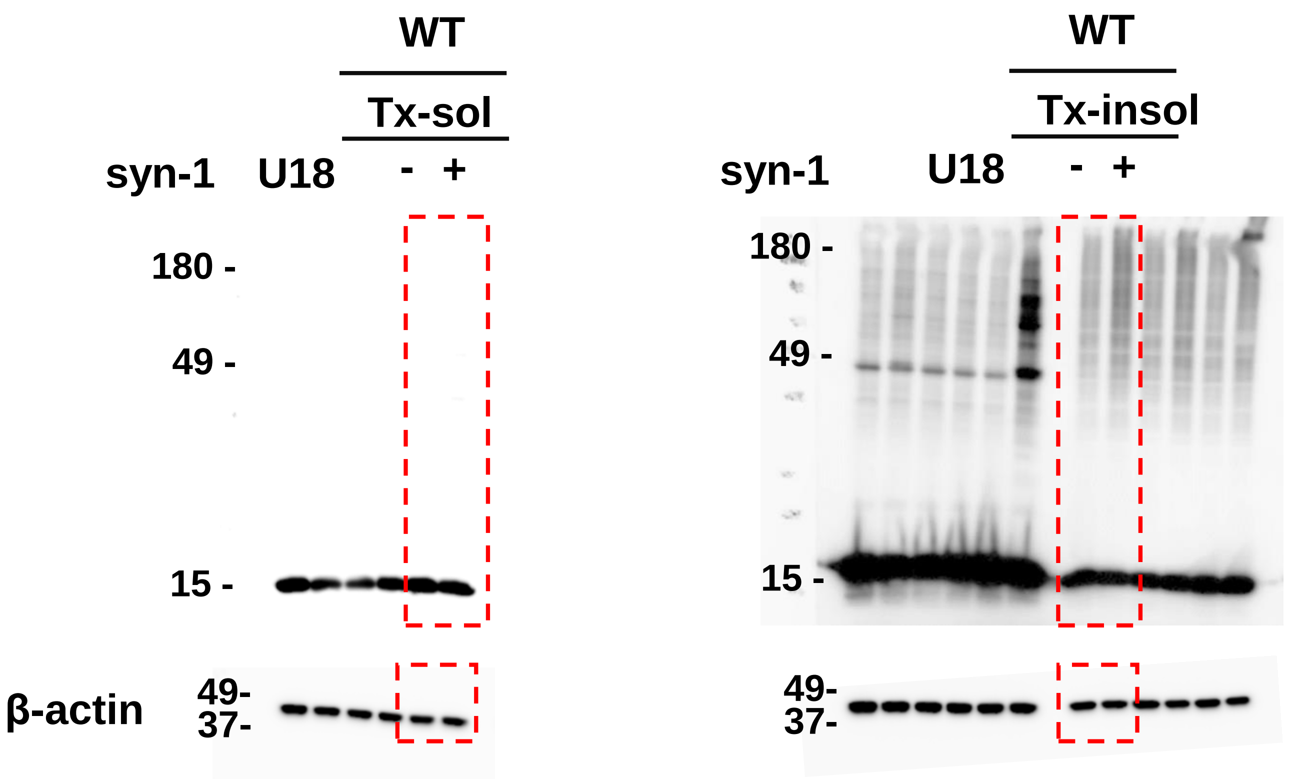

Figure 5G

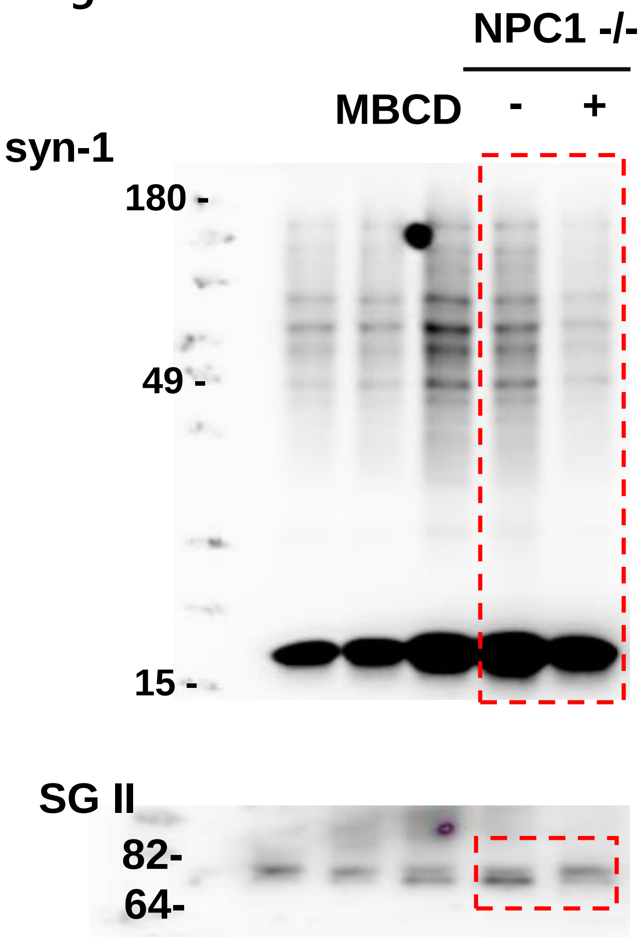

Figure 5H

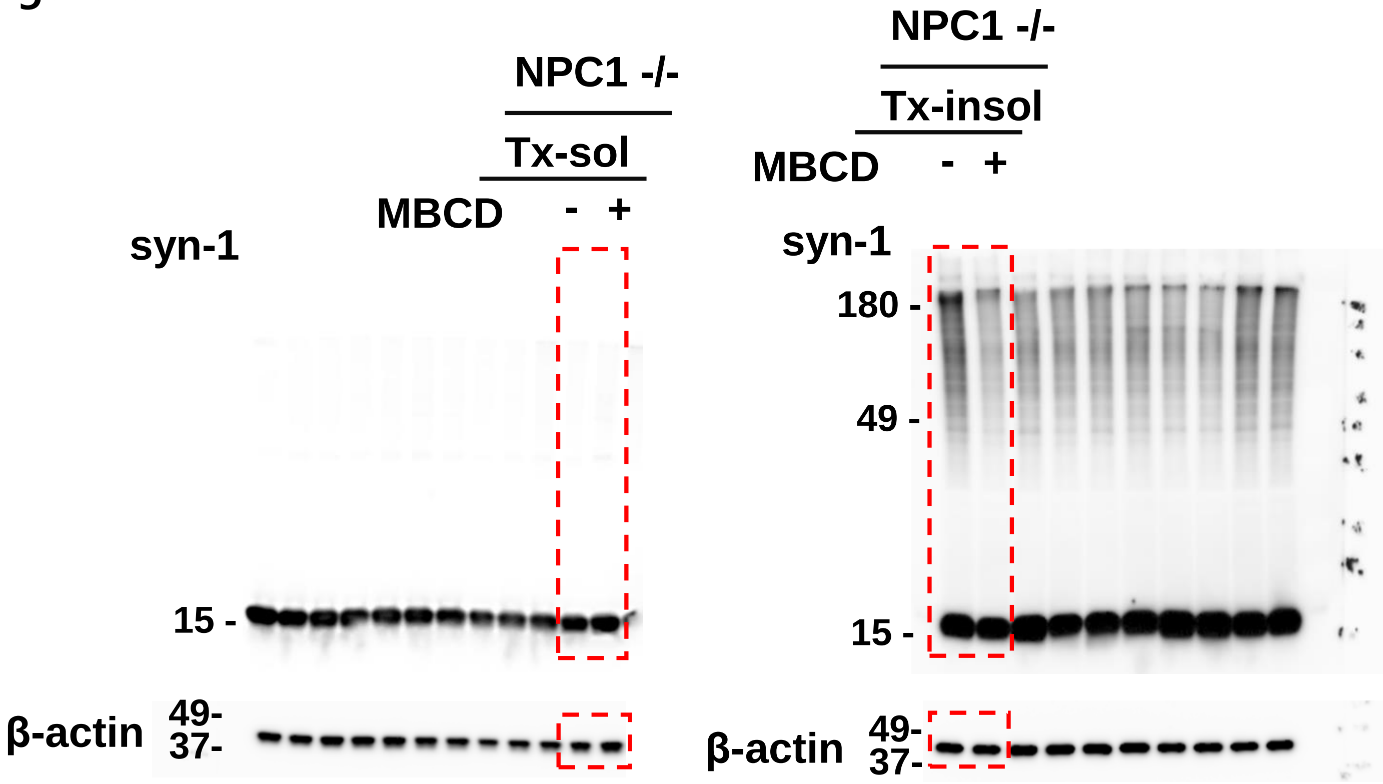

Figure 5J

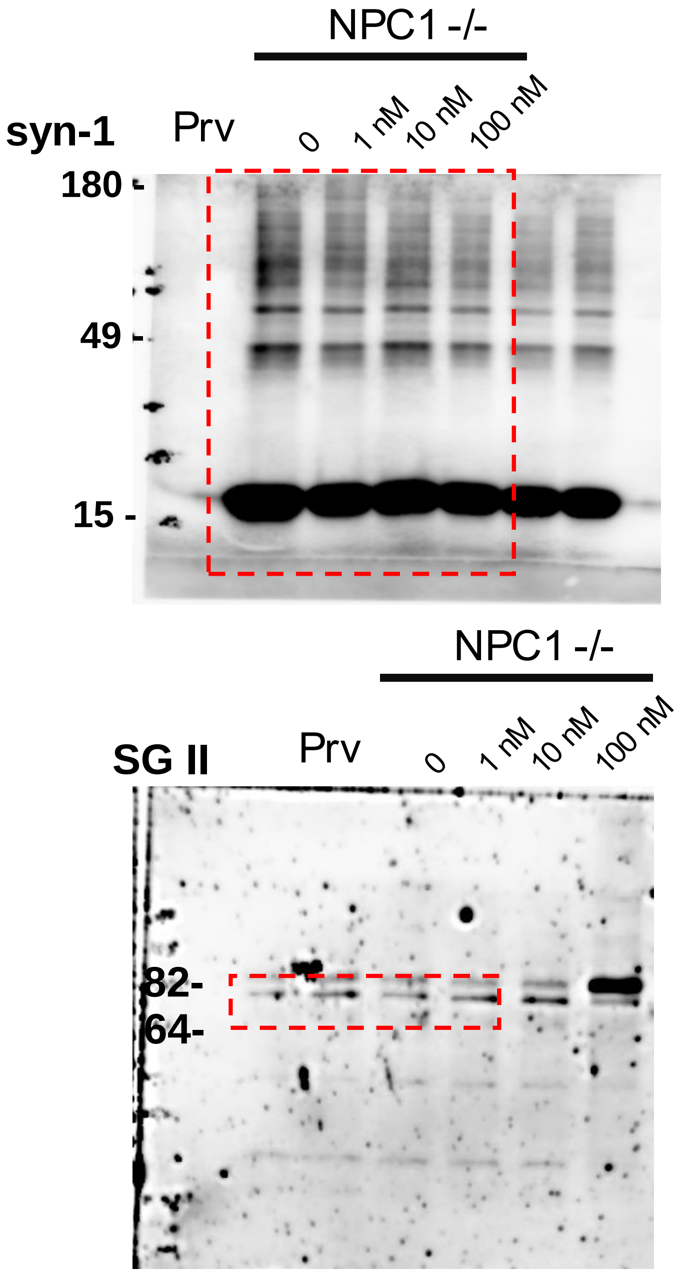

Figure 5K

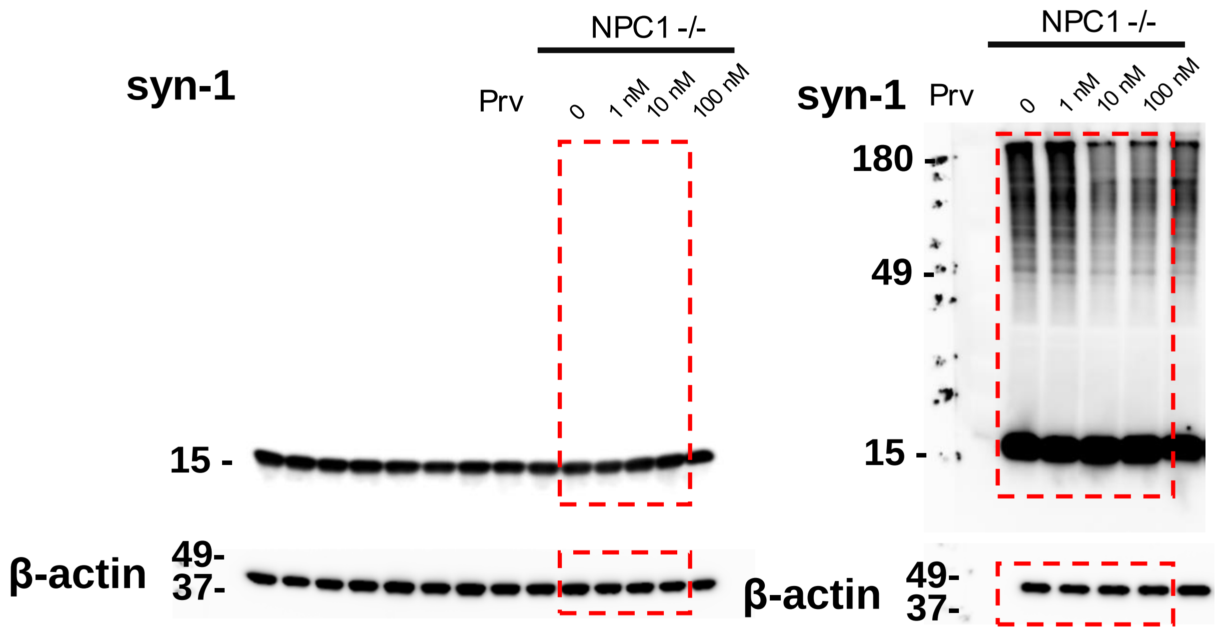

Figure 5M

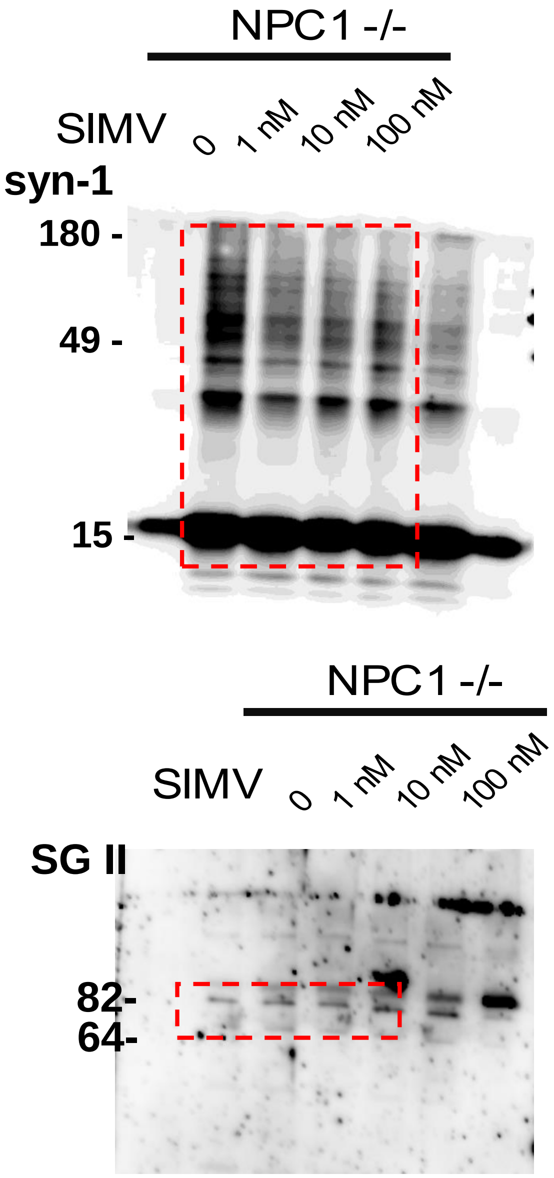

Figure 5N

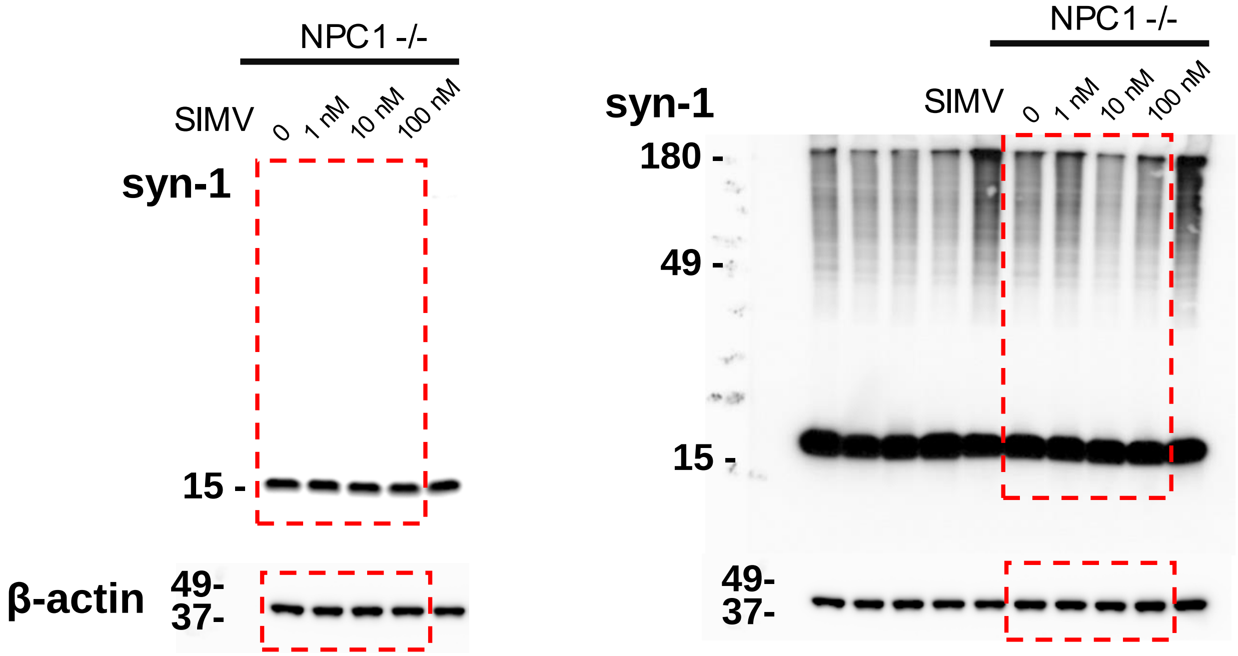

Figure 6D

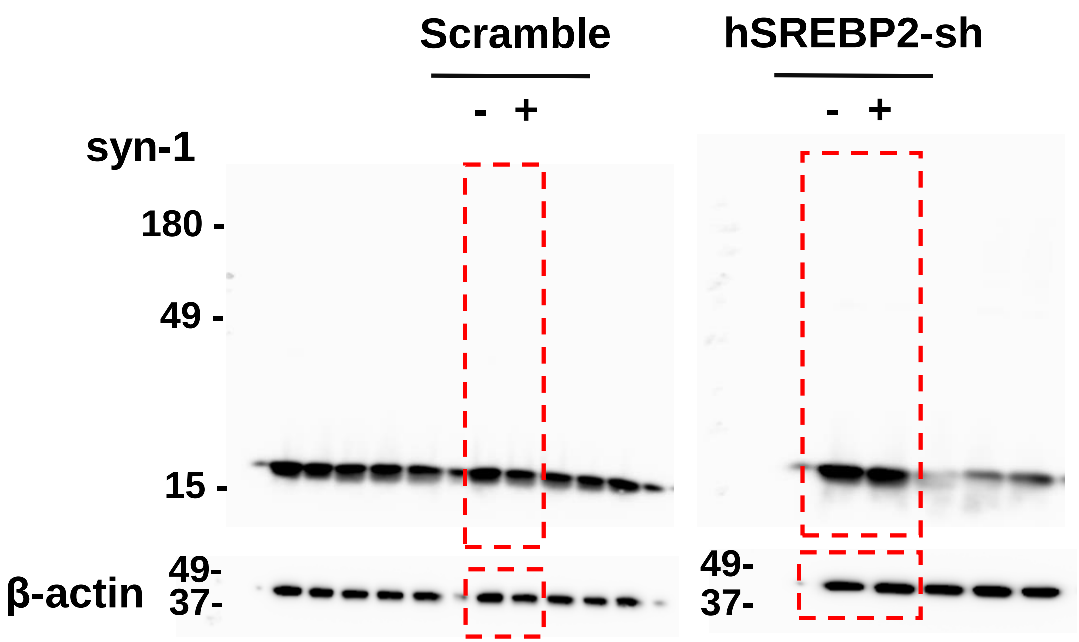

Figure 6E

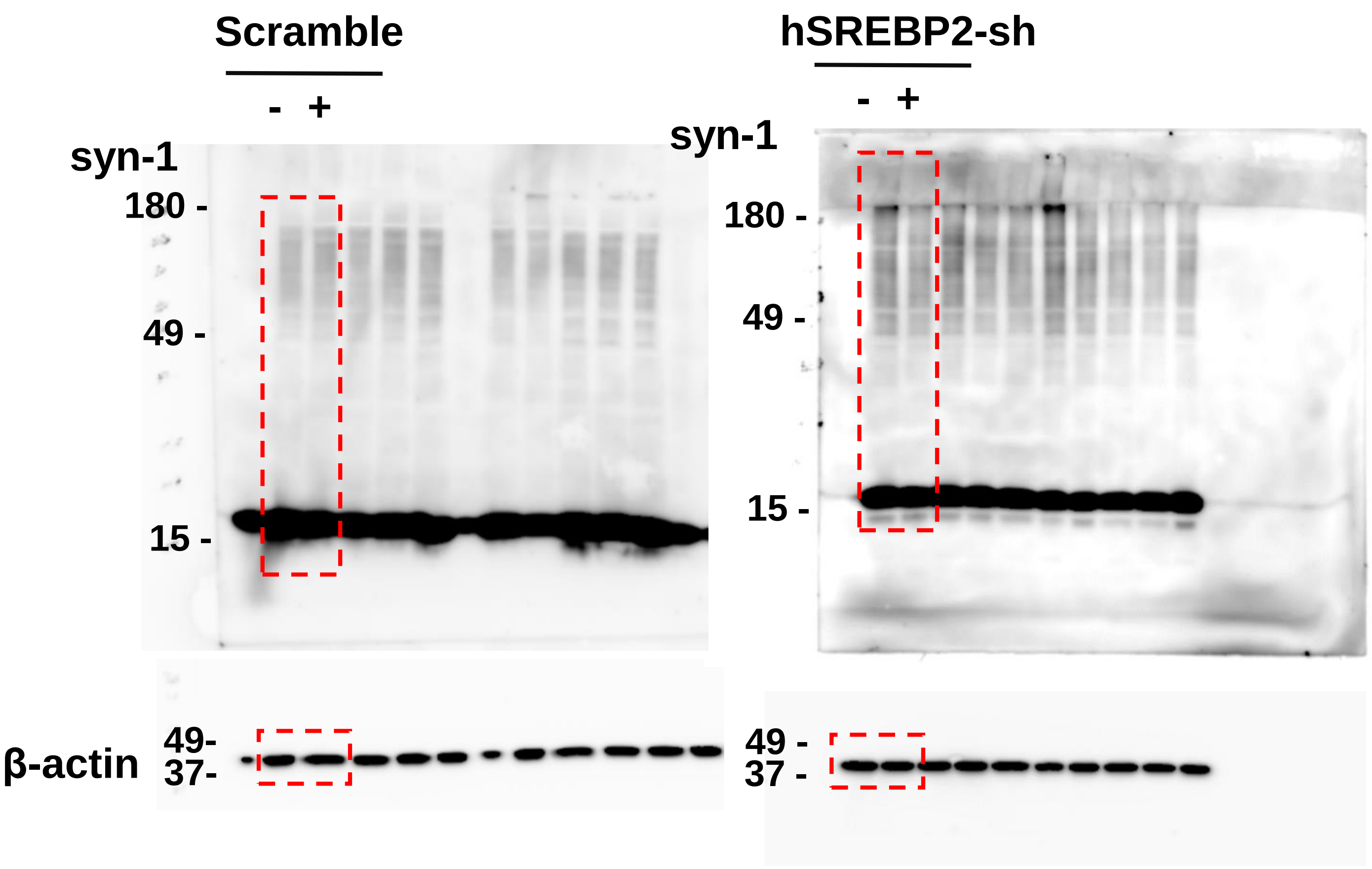

Figure 6F

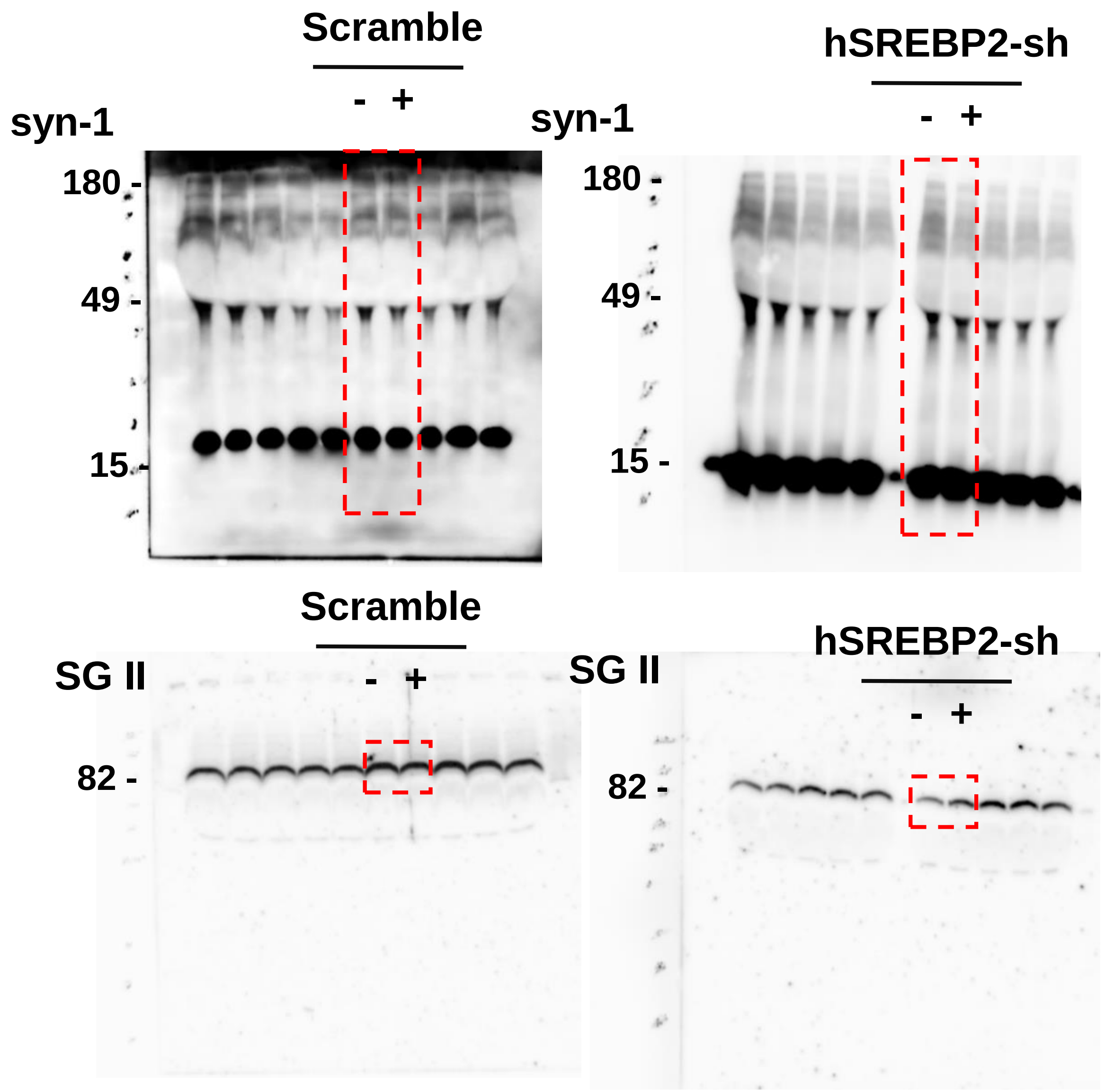
Scramble
- +
syn-1
180 -
49 -
15 -
hSREBP2-sh
- +
syn-1
180 -
49 -
15 -
Scramble
SG II
- +
82 -
hSREBP2-sh
SG II
- +
82 -

Supplementary Figure 1A

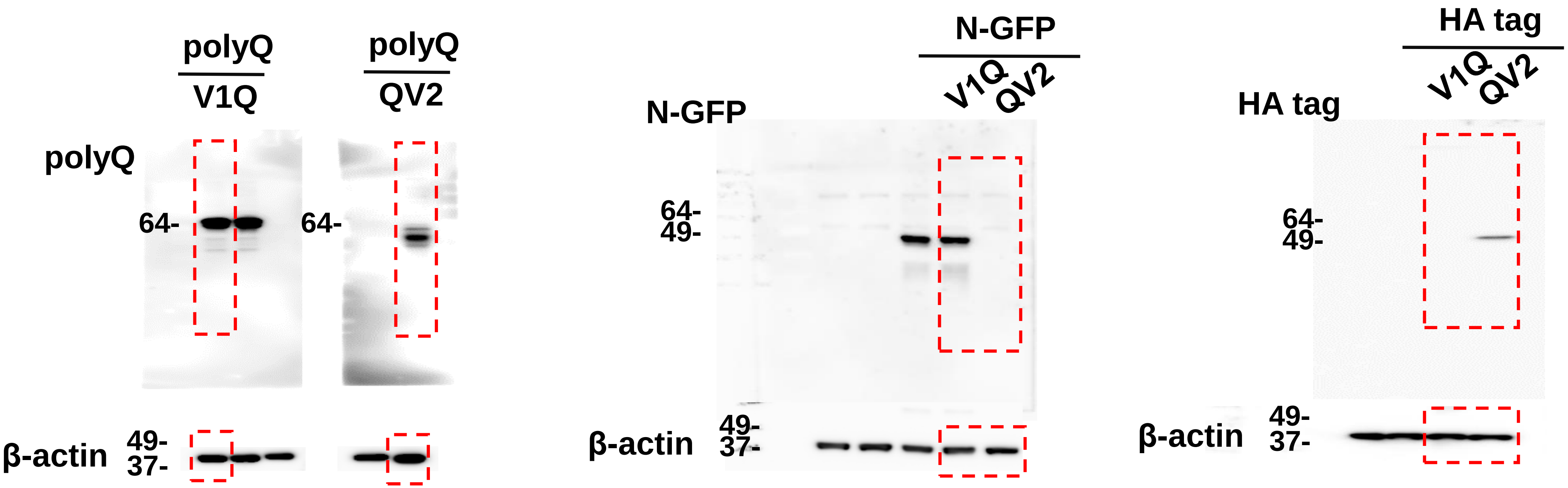

Supplementary Figure 3

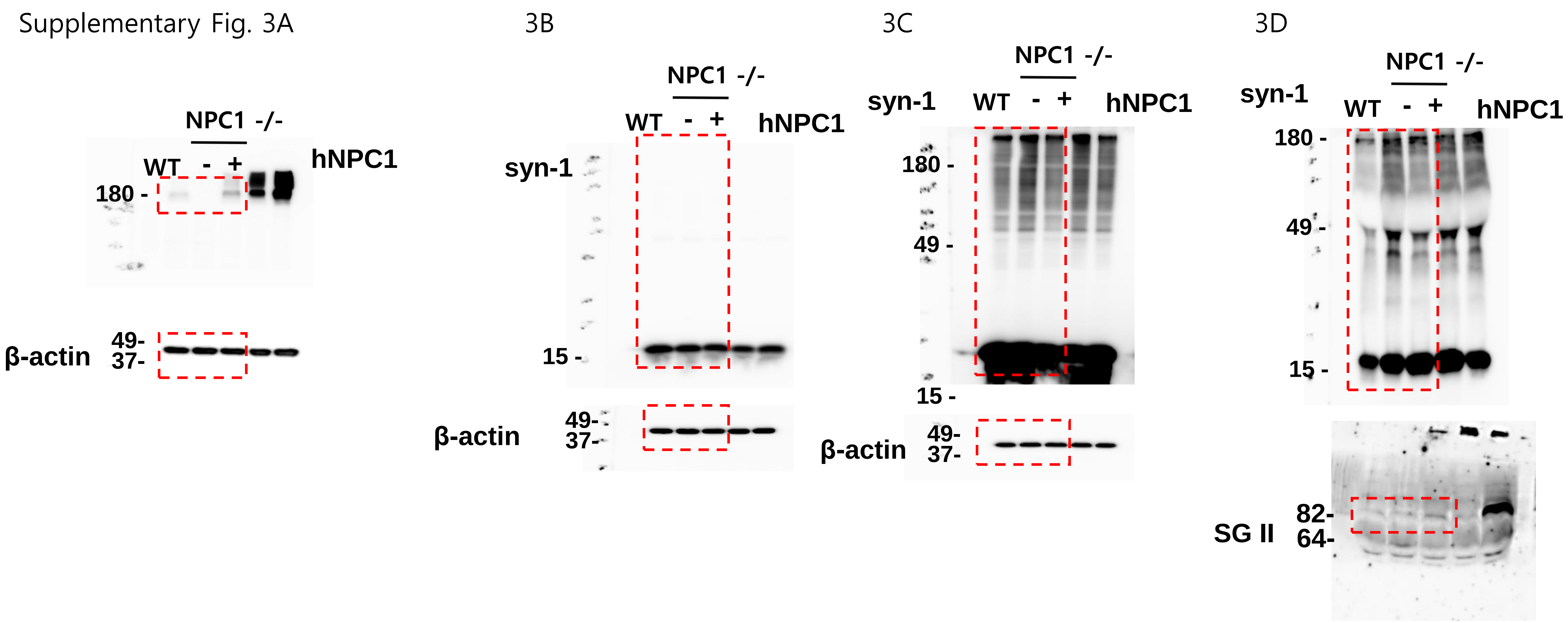

Supplement: Supplementary file 8 — Original Data File [file 41419_2023_5977_MOESM8_ESM.pdf]
